# Comprehensive Analysis of the 16p11.2 Deletion and Null *Cntnap2* Mouse Models of Autism Spectrum Disorder

**DOI:** 10.1371/journal.pone.0134572

**Published:** 2015-08-14

**Authors:** Daniela Brunner, Patricia Kabitzke, Dansha He, Kimberly Cox, Lucinda Thiede, Taleen Hanania, Emily Sabath, Vadim Alexandrov, Michael Saxe, Elior Peles, Alea Mills, Will Spooren, Anirvan Ghosh, Pamela Feliciano, Marta Benedetti, Alice Luo Clayton, Barbara Biemans

**Affiliations:** 1 PsychoGenics, Inc., Tarrytown, NY, United States of America; 2 Department of Psychiatry, Columbia University, New York, NY, United States of America; 3 Roche, Basel, Switzerland; 4 Department of Molecular Cell Biology, Weizmann Institute of Science, Rehovot, Israel; 5 Cold Spring Harbor Laboratory, Cold Spring Harbor, NY, United States of America; 6 Simons Foundation Autism Research Initiative, New York, NY, United States of America; Université de Bordeaux and Centre National de la Recherche Scientifique, FRANCE

## Abstract

Autism spectrum disorder comprises several neurodevelopmental conditions presenting symptoms in social communication and restricted, repetitive behaviors. A major roadblock for drug development for autism is the lack of robust behavioral signatures predictive of clinical efficacy. To address this issue, we further characterized, in a uniform and rigorous way, mouse models of autism that are of interest because of their construct validity and wide availability to the scientific community. We implemented a broad behavioral battery that included but was not restricted to core autism domains, with the goal of identifying robust, reliable phenotypes amenable for further testing. Here we describe comprehensive findings from two known mouse models of autism, obtained at different developmental stages, using a systematic behavioral test battery combining standard tests as well as novel, quantitative, computer-vision based systems. The first mouse model recapitulates a deletion in human chromosome 16p11.2, found in 1% of individuals with autism. The second mouse model harbors homozygous null mutations in *Cntnap2*, associated with autism and Pitt-Hopkins-like syndrome. Consistent with previous results, 16p11.2 heterozygous null mice, also known as Del(7Slx1b-Sept1)4Aam weighed less than wild type littermates displayed hyperactivity and no social deficits. *Cntnap2* homozygous null mice were also hyperactive, froze less during testing, showed a mild gait phenotype and deficits in the three-chamber social preference test, although less robust than previously published. In the open field test with exposure to urine of an estrous female, however, the *Cntnap2* null mice showed reduced vocalizations. In addition, *Cntnap2* null mice performed slightly better in a cognitive procedural learning test. Although finding and replicating robust behavioral phenotypes in animal models is a challenging task, such functional readouts remain important in the development of therapeutics and we anticipate both our positive and negative findings will be utilized as a resource for the broader scientific community.

## Introduction

The creation of mouse models of complex human diseases poses a formidable challenge, from recapitulating the genetic or environmental insult, to the optimization of endpoint measures chosen to maximize translational power for the development of therapeutics. The challenges, which often lead to great variability of results across labs, include the different technologies available for the generation of murine models, the varying mouse genetic backgrounds, and putative differences in the experimental procedures used for phenotyping [1]. In addition, in disorders of known partial or variable penetrance, environmental factors are thought to play a major role. For example, the phenotype of murine models of Huntington’s disease has yielded somewhat inconsistent results among different laboratories, despite the apparent simplicity of the human genetic cause (a consistent expansion of a CAG repeat in the huntingtin gene). An added difficulty arises in autism spectrum disorder (ASD) due to the enormous variety of genetic factors, with an estimated 230–400 autism susceptibility genes (even more, depending on the method of assessment [2–4]), in addition to environmental risk factors that are hypothesized to contribute to the behavioral phenotypic spectrum.

Although robust mouse models exist for some syndromic forms of ASD, such as Fragile X [5], models for ASD have been lagging behind due to the lack of knowledge about the underlying genetic causes. While models based on pharmacological manipulation [6–8] or phenotypic face validity have been available [9, 10], increased knowledge of the genetic architecture of ASD has created an opportunity for the development of animal models of autism with construct and etiological validity. As these latter models may provide a translational platform for drug development, exploring their robustness is a matter of urgency and we, therefore, designed a project to further study five relatively novel ASD models, two of which are described here.

Genes involved in ASD seem to disrupt synaptic function and lead to an imbalance between excitatory and inhibitory control in brain circuits [11–13]. Synaptic cell adhesion molecules, namely, neurexins, neuroligins and contactins, play a critical role in the formation and function of synapses and are represented in this project by the Cntnap2 model. Scaffolding proteins, also fundamental for synaptic function, are represented by inclusion of two different Shank3 models. Ion channels, such as potassium, sodium, and calcium channels are represented here by inclusion of the Cacnac1c model. Signal transduction and transcription, namely, FMR1, TSC1, PTEN, mTOR and MECP 2 were not included in this project although at least two of these models, FMR1 and MECP2, are routinely used in drug screening projects due to their very robust phenotype [14, 15]. Copy number variation and deletions are represented by the 16p11 deletion model. Other genes coding receptors, enzymes, and transport such as UBE3A, were considered but not included do to unavailability.

Here we provide an extensive behavioral characterization and analysis of two mouse models of autism, the 16p11.2 heterozygous null (16p11.2 *df*/+) and *Cntnap2* homozygous null (*Cntnap2* -/-) mice, chosen because of their strong construct validity and the robust human genetic evidence that implicate these loci in autism [16–21]. In addition, these models are widely used in the scientific community (for example [22–24]) and freely available through The Jackson Laboratories (http://www.jax.org/). Subsequent publications will describe additional models that were included within our broader project.

Microdeletions and microduplications at human chromosome 16p11.2 occur in approximately 1% of idiopathic ASD cases [16–18]. The most common deletion (from 29.5 Mb to approximately 30.1 Mb, which covers approximately 600 kilobases involving 29 annotated genes) has been reproducibly associated with ASD and epilepsy [19]. A sub-deletion of 200 kilobases (from 28.74 Mb to approximately 28.95 Mb) is also associated with developmental delays and obesity [25, 26]. Individuals with a 16p11.2 deletion show wide phenotypic variation ranging from mild to normal intellectual ability and frequently present language delays, cognitive deficits, ASD, seizures, macrocephaly, and obesity [27–32]. This phenotypic variation is assumed to arise from differences in genetic penetrance and genetic heterogeneity at other interacting genetic loci [27, 31].

The 16p11.2 *df*/+ (also known as Del(7Slx1b-Sept1)4Aam) mice, engineered to mimic the 600 kb human deletion, are reported to have normal social behavior and grip strength, have lower body weight than controls as neonates, display hyperactivity in a novel environment, show disturbances in the diurnal rhythm, stereotypic behaviors and partial prenatal lethality [22]. A 16p11.2 deletion model encompassing a slightly different syntenic region was also reported to present hyperactivity, motor deficits and lack of habituation [23]. Germline transmission is reduced in the C57B6/J pure background to the point that normal breeding is not possible (Dr. Cathleen Lux, The Jackson Laboratory, and Dr. Nicholas Katsanis, Duke University; independent personal communication). Therefore, while we initially sought to characterize all models on the same C57B/6J background, the results described in this article for the 16p11.2 deletion model were obtained using a mixed (129/C57) background although the model has been successfully bred onto a C57B6/N background (Taconic Farms, Dr. Alea Mills, personal communication).

The *Cntnap2* -/- mouse model was engineered to have the first exon of *Cntnap2* replaced by a neo gene, resulting in the absence of the CNTNAP2 protein in the brains in homozygous mice [33]. Recessive loss-of-function mutations in CNTNAP2 cause Pitt-Hopkins-like syndrome [20, 34], symptoms of which include severe intellectual disability, lack of speech and seizures. In addition, a homozygous single base pair deletion, resulting in a truncated protein, has been shown to cause Recessive Symptomatic Focal Epilepsy, which presents with autistic symptoms and other characteristics including language regression, cortical dysplasia, epilepsy, macrocephaly, diminished reflexes, hyperactivity, impulsivity, aggression, and intellectual disability [21].

A previous publication reported that *Cntnap2* -/- mice had normal behavior in anxiety-like, a visuo-spatial, startle and sensorimotor gating tests, but show decreased social preference, abnormal neonatal ultrasonic vocalizations, increased grooming, decreased alternations in a T-maze, and seizures. Mice were also hyperactive, showed faster reactions to pain stimulation, and faster completion of an olfactory task. Surprisingly, they showed enhanced motor coordination in a rotarod task [24].

A number of papers investigating the reasons behind the failure in the translation to the clinic of findings from preclinical trials, identified replication of the original findings to test the robustness of a phenotype as an important way to reduce failure rate [35]. In principle, the most robust models of autism should display replicable behavioral deficits in the core autism domains, namely social behavior, communication, and behavioral flexibility [36], even when tested by different laboratories, even when using slightly different experimental protocols [37].

We carried out a large phenotypic project to assess, replicate and extend the published phenotypes with the ultimate goal of setting up a drug screening battery for candidate therapeutics. As such, we sought to confirm and extend published data in order to ensure a strong experimental foundation of these important mouse models. We chose tests that measure social behavior (the three-chamber and reciprocal social interaction), behavioral flexibility and repetitive behavior (T-maze discrimination reversal and marble burying), developmental milestones (neonatal tests), vocalization (two tests measuring USV) and motor-gating (prepulse inhibition of startle). In addition, we utilized two novel high-throughput technologies, NeuroCube and SmartCube, which offer a comprehensive, unbiased, quantitative assessment of several domains of relevance for ASD, and facilitate the discovery of novel aspects of the models’ phenotypes that can be exploited for future drug development and screening [1, 38, 39]. Our aim was to identify robust, reliable behavioral signatures for possible future studies by taking a broader approach that included tests of developmental milestones and sensorimotor function, because early or ‘low-level’ differences may have downstream effects that impact interpretation of results obtained from ‘higher level’ cognitive tests.

All mice were obtained from The Jackson Laboratory after being generously donated by the principal investigators and their respective institutions that generated them. Breeder and experimental animals were housed under enriched conditions (see Materials and Methods). We present here an assessment of the 16p11.2 *df*/+ and *Cntnap2* -/- murine models of autism and compare results against those obtained in different labs.

## Results

### SmartCube

SmartCube is a high-throughput automated behavioral platform for rapid and comprehensive phenotyping of mutant models and detection of behavior patterns influenced by drugs, mutations, or any other manipulation. SmartCube employs computer vision and mechanical actuators to detect spontaneous behavior and reactions to particular challenges such as motor, startling and aversive stimuli. It was designed to capture readouts such as locomotion, rearing, trajectory complexity, body posture and shape, simple behaviors, and behavioral sequences, among others [38, 40–42]. In a phenotyping project, the difference between mutant and wild type (WT) mice is assessed using supervised machine learning algorithms to comb the set of more than 1400 features collected (see S4 Methods). To visualize and quantify results, data are plotted in the 2D feature space that best separates the two groups, and the overlap between the two datasets is used as discrimination index. To assess the robustness of the separation, the machine learning algorithm is trained and tested many times with different data subsets using correct and randomized labels. The overlap between the resulting distributions of discrimination indexes (training with either correct or randomized labels) is used to estimate the probability that the results could be due to chance.

**Fig 1:**
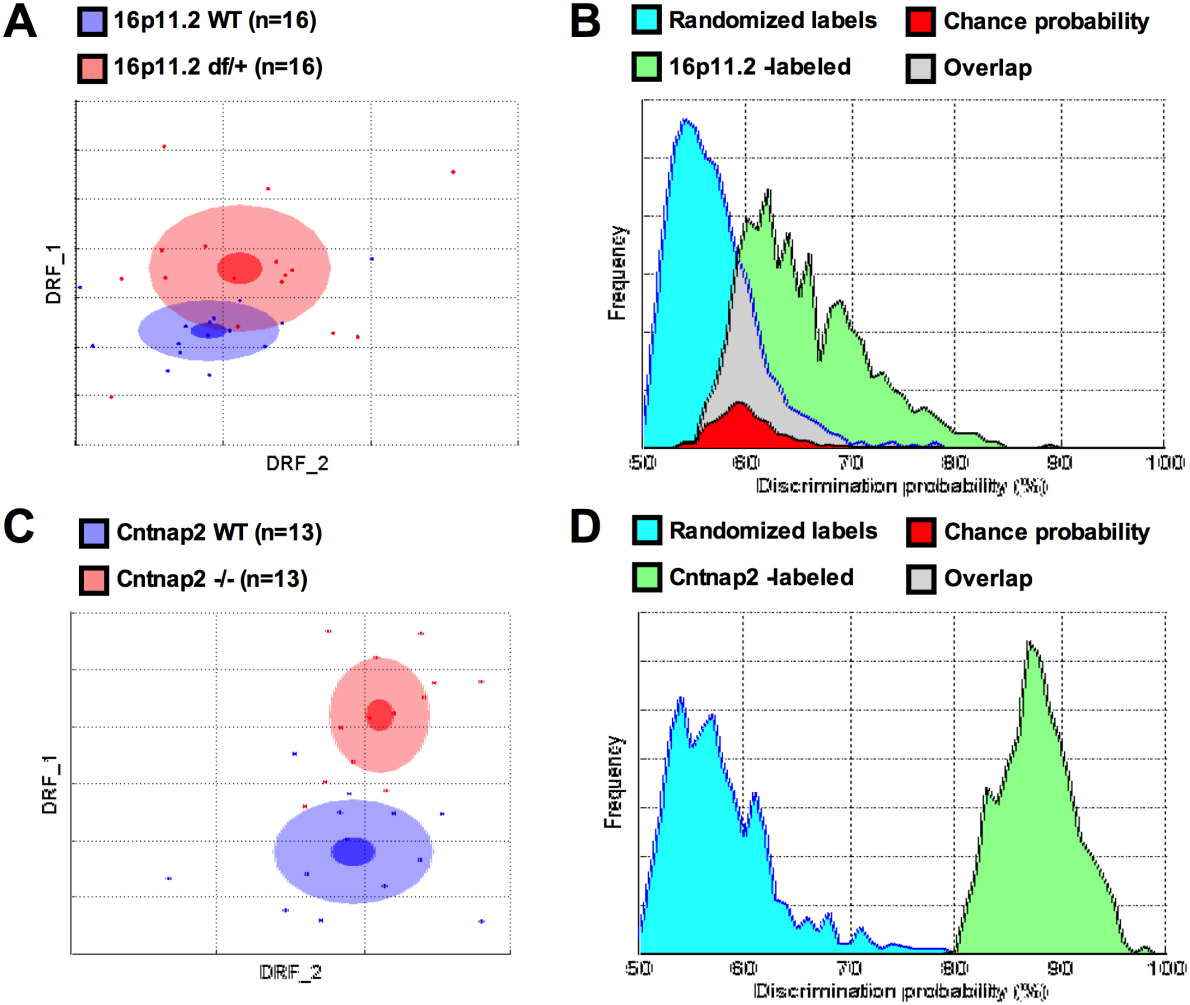
SmartCube found different degrees of separation between 16p11.2 df/+ and Cntnap2 -/- as compared to their control littermates. A & C: To build a 2D representation of the multidimensional space in which the two groups are best separated, we first find statistically independent combinations of the original features, pick the two new composite features (drf 1 and 2) that best discriminate between the two groups, and used them as x- and y-axes (see S4 Methods). As in Principal Component Analyses, these two axes represent the combinations of uncorrelated feature transforms that account for most of the variance. Each dot represents either a WT (blue) or a mutant (red) mouse. The center, small and large ellipses are the mean, standard error and standard deviation of the composite features for each group. The overlap between the groups is used to calculate the discrimination index, which measures how reliably a classifier can be trained to discriminate between the two groups (the more overlap, the worse the discrimination). B & D: To estimate how likely it is that such separation is simply due to chance, the obtained classifier is challenged many times with correctly labeled samples (“WT” and “mutant”; see the green distribution) or with randomized labels (blue distribution). The overlap between these two distributions (in red) represents the probability of obtaining the observed discrimination by chance. A: The 16p11.2 df/+ standard deviation ellipse overlaps considerably with the WT control ellipse. Note the spread and position variability of the individual mice; B: A 65% discrimination between 16p11.2 df/+ and WT mice could be found by chance with p < .12; C: The Cntnap2 -/- model separates well from the WT group; D: A 88% discrimination index for the Cntnap2 -/- versus WT comparison can be found by chance with p < .0002.

**Fig 2:**
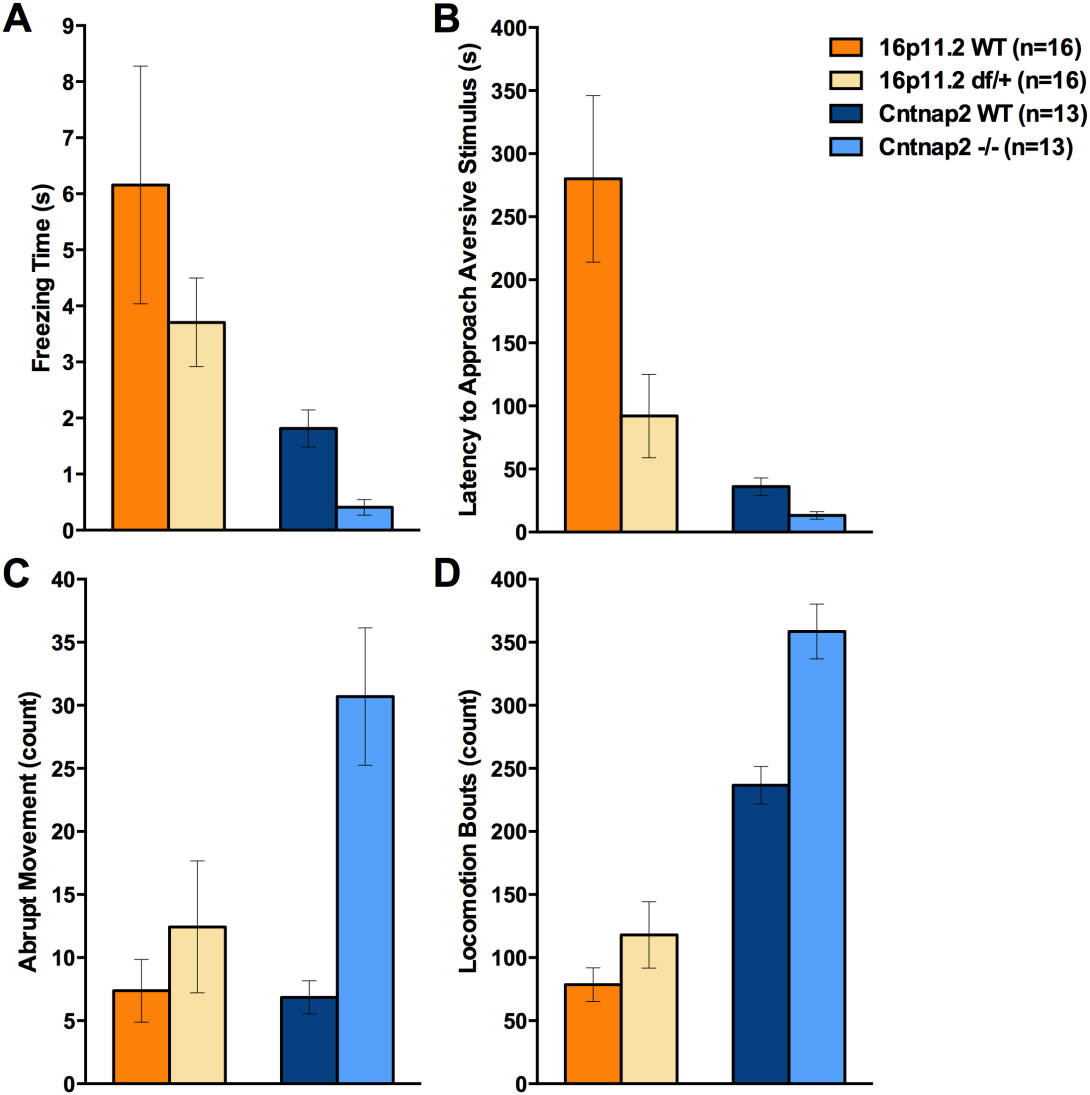
SmartCube found the behavioral features that showed larger contributions to an overall discrimination. A: Both 16p11.2 df/+ mice and Cntnap2 -/- tended to freeze less than their corresponding WT mice; B: Both 16p11.2 df/+ and Cntnap2 -/- mice tended to approach a stimulus faster than their corresponding WT mice; C: Cntnap2 -/- mice showed considerably more abrupt movements than WT mice; D: Cntnap2 -/- showed more locomotion bouts as compared to the WT mice.

The discrimination between the 16p11.2 WT and *df*/+ mice only reached 65% (Fig 1A and 1B; *p* < 0.11). The SmartCube measurements that showed comparatively larger differences between 16p11.2 WT and *df*/+ mice, contributing to the small non-significant overall difference, were higher complexity of the locomotor trajectory and shorter latencies to approach a visual stimulus in the *df*/+ compared to WT mice. Collectively, these characteristics are consistent with a hyperactive phenotype in the *df*/+ mice (Fig 2; S5 Table).

In contrast, the discrimination values between the *Cntnap2* WT and *Cntnap2* -/- mice reached 88% (Fig 1C and 1D; *p* < .0001). The behavioral features that contributed to these values included higher complexity of the trajectory, more and faster locomotion, more short steps, and increased frequency of abrupt movements. Collectively, these characteristics again suggest a hyperactive phenotype in the mutant mice. In addition, the SmartCube analysis suggested reduced motor competence, as seen by increased frequency of abrupt movements in the *Cntnap2* -/- mice (Fig 2C and 2D; S6 Table). Interestingly, all four top features showed similar trends in the two models (Fig 2).

### NeuroCube

Although motor abnormalities are not part of the core symptomatology of autism, it has been reported that up to 79% of children with autism may have movement impairments [43, 44] and there is evidence of underlying abnormal connectivity within the motor cortex [45]. Impairments are also observed in infant siblings of children with autism that are at high risk for autism themselves [46], and are more common in concordant siblings with autism compared to discordant sibling pairs [47]. As such, we used Neurocube to assess this highly quantifiable behavior. NeuroCube is a gait analysis system that captures locomotion, gait geometry and dynamics, and body motion, among other features [38]. The analysis of the NeuroCube features proceeds in the same manner as that described for SmartCube (see Materials and Methods and S4 Methods) although, for this technology, different domains can be analyzed separately (Table 1).

**Table 1:** NeuroCube Results at P30 and P60.

	16p11.2		Cntnap2	
Domain	P30	P60	P30	P60
**All features**	89%*	83%*	84%*	83%*
**Average speed**	56%	70%	78%*	78%*
**Gait**	67%	73%*	88%*	92%*
**Imaging**	81%*	74%*	63%	71%
**Rhythm**	63%	61%	57%	68%
**Body position**	58%	53%	54%	56%
**Paw position**	90%*	72%*	63%	54%

Analysis of NeuroCube data showed that the 16p11.2 df/+ mice were significantly different from the WT control mice overall, and, in particular, for paw imaging and position features. Gait was different only at P60. The Cntnap2 -/- mice were also different overall and, in particular, in their speed and gait features. The numbers shown are the maximal discrimination found between the two groups.

*Indicates that the associated *p*-value was less than .05.

**Fig 3:**
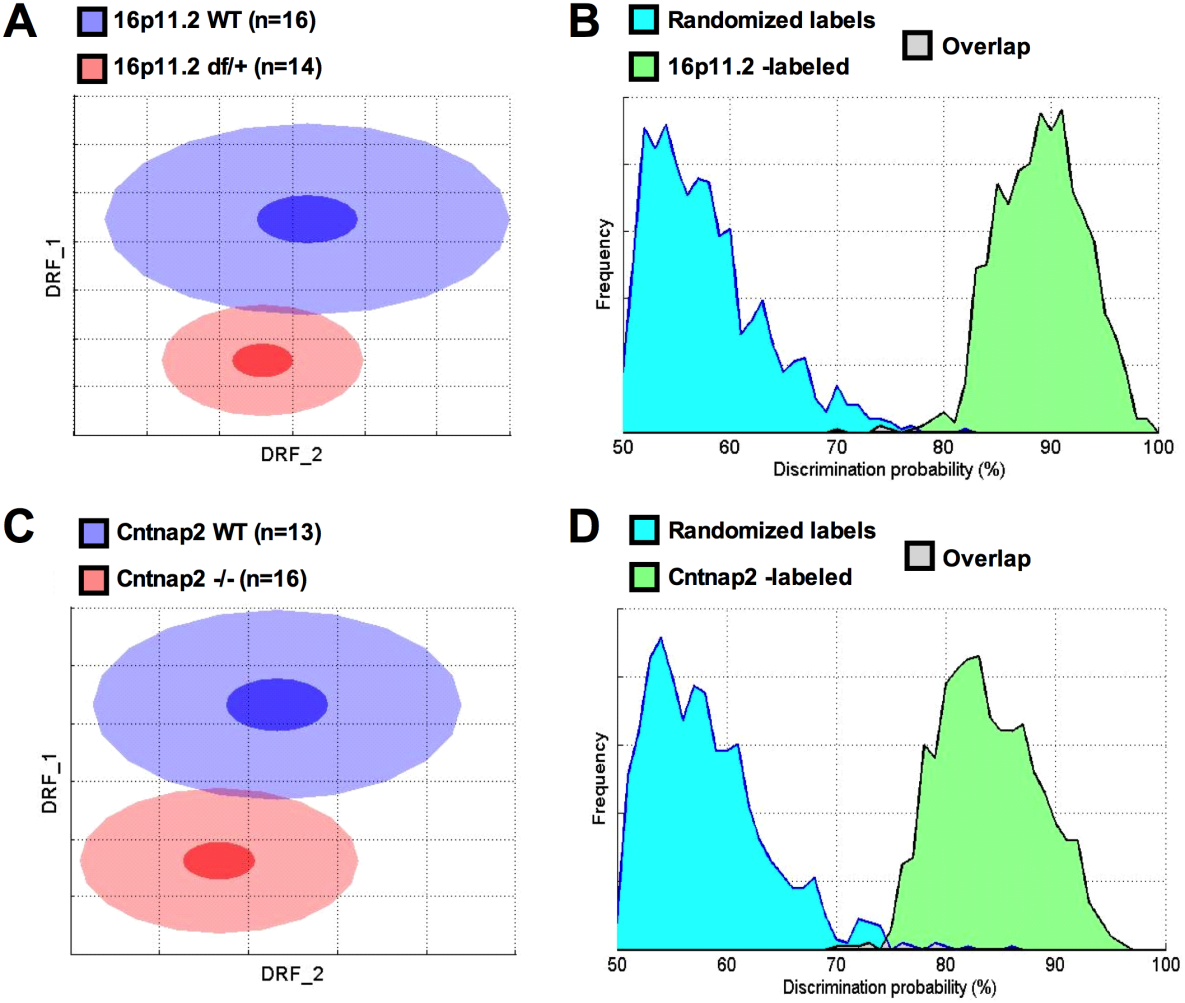
NeuroCube found similar degrees of separation between 16p11.2 df/+ and Cntnap2 -/- as compared to their corresponding WT control littermates. A & C: To build a 2D representation of the multidimensional space in which the two groups are best separated, we first find statistically independent combinations of the original features, pick the two new composite features (drf 1 and 2) that best discriminate between the two groups, and used them as x- and y-axes (see S4 Methods). As in Principal Component Analyses, these two axes represent the uncorrelated feature transformations that account for most of the variance. Each dot represents either a WT (blue) or a mutant (red) mouse. The center, small and large ellipses are the mean, standard error and standard deviation of the composite features for each group. The overlap between the groups is used to calculate the discrimination index, which indicates how reliably a classifier can be trained to discriminate between the two groups (the more overlap, the worse the discrimination). B & D: To estimate how likely it is that such separation is simply due to chance, the obtained classifier is challenged many times with correctly labeled samples (“WT” and “mutant”; see the green distribution) or with randomized labels (blue distribution). The overlap between these two distributions (in red) represents the probability of obtaining the observed discrimination by chance. A: At P30 the 16p11.2 df/+ standard deviation ellipse overlapped to a small extent with the WT control ellipse; B: An 89% discrimination between 16p11.2 df/+ and WT mice could be found by chance with p< .0003; C: At P30 the Cntnap2 -/- model separated well from the WT group; D: An 84% discrimination index can be found by chance with p< .003.

**Fig 4:**
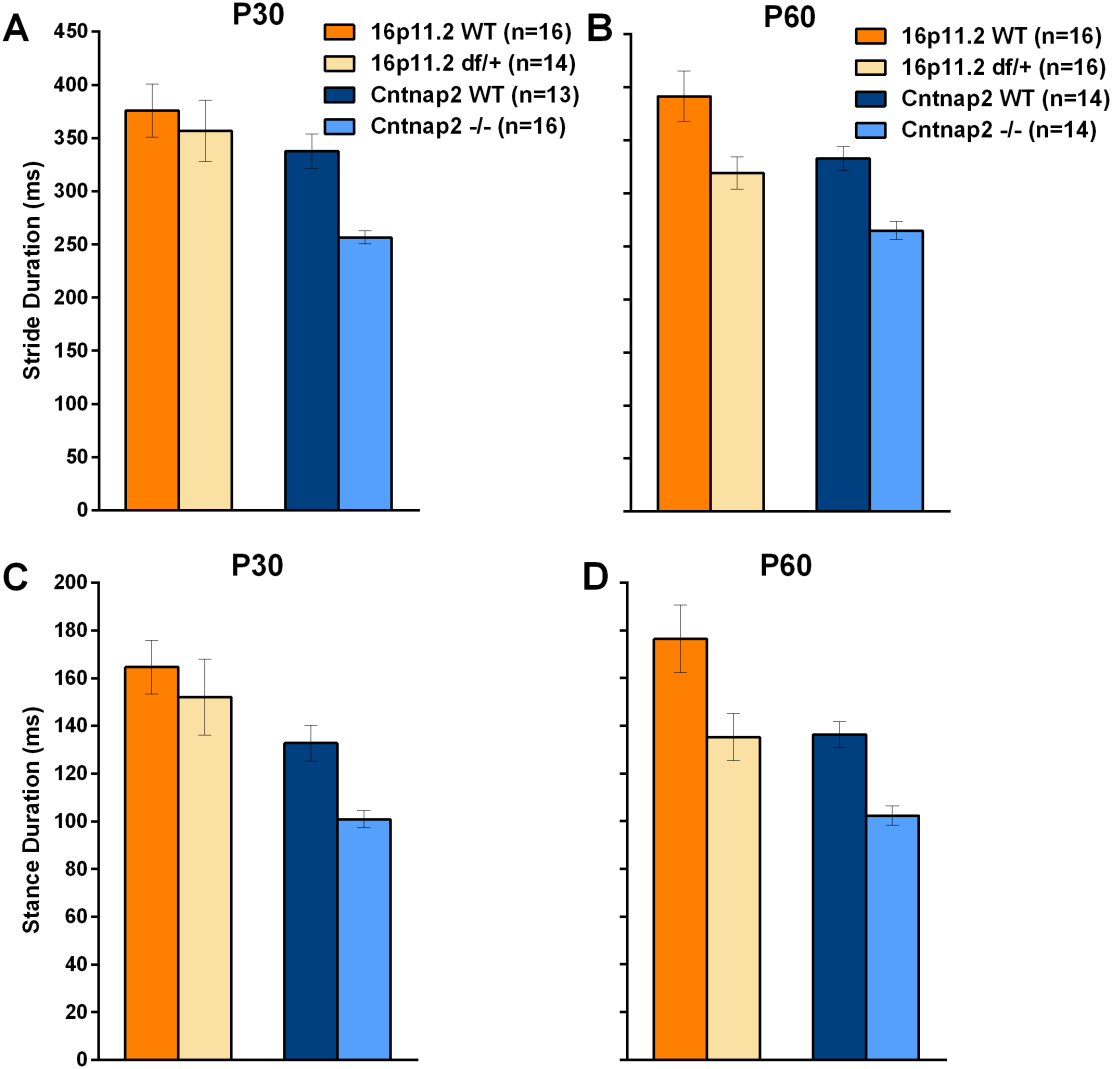
A: Stride duration was shorter for the Cntnap2 -/- mice than WT controls at the two ages studied. In the 16p11.2 df/+ mice this was true only at P60; B: Stance duration showed the same pattern. Data shown are means ± SEM.

In NeuroCube 16p11.2 *df*/+ mice showed significant differences overall (Fig 3A and 3B; Table 1), particularly in gait, paw image, and paw position. Investigation of gait features revealed that the length of the stride was not different, but the durations of the stride and stance were slightly reduced at P60 (Fig 4; Table 1). Hind base seemed wider for the 16p11.2 *df*/+ than for the WT mice. The percent of time corresponding to the swing phase was not different between the two groups. The paw area was apparently smaller in the *df*/+ mice although the paw image intensity was not particularly different (apart from an apparent reduction in the hind limb paw intensity at P30; S7 Table).

**Fig 5:**
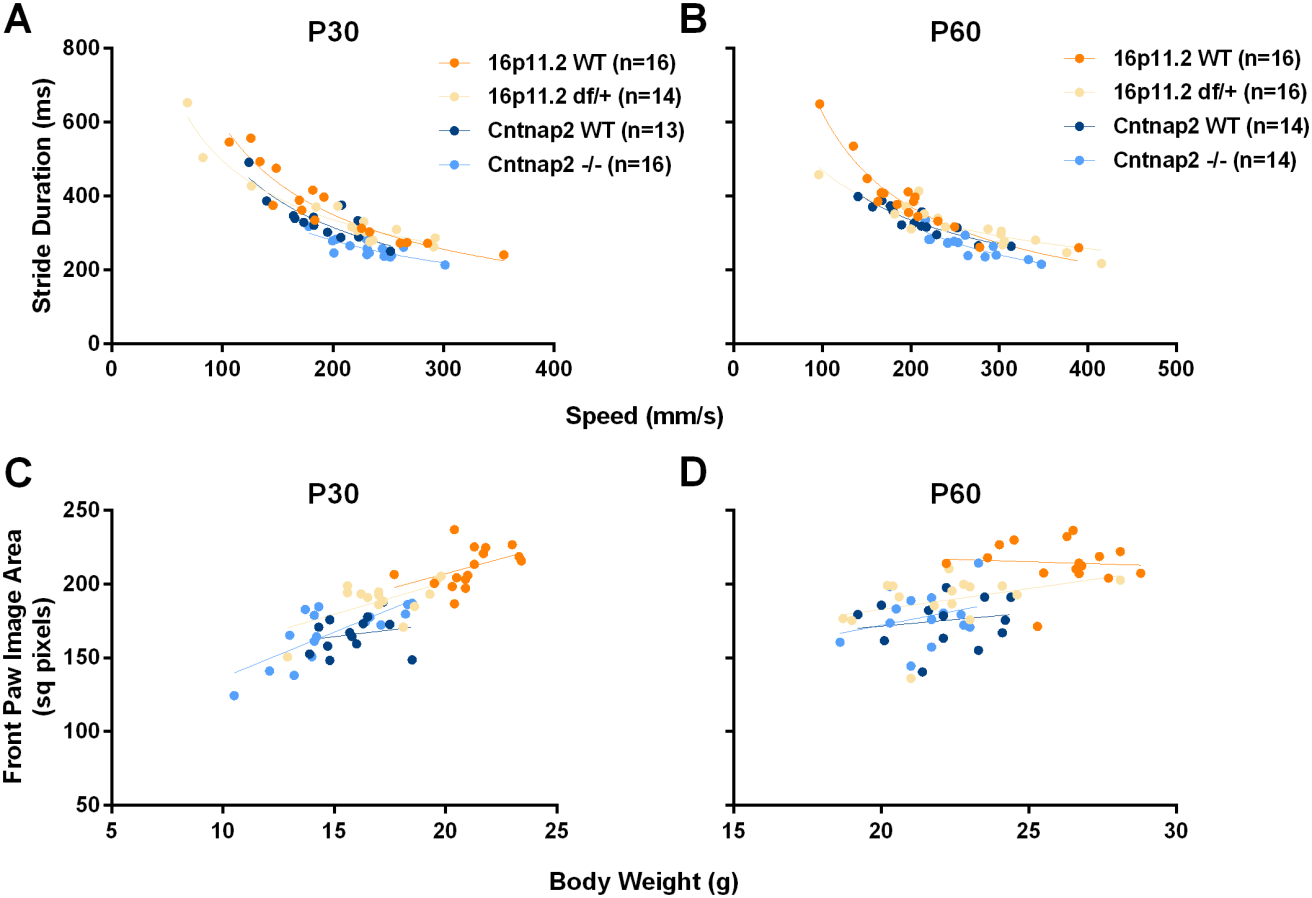
Correlation analysis of gait features as a function of speed or body weight. A & B: Stride duration as measured in NeuroCube showed a non-linear correlation with locomotor speed, i.e., the faster a mouse runs the shorter the stride duration. The lines show power regressions, which provided the best fit; C & D: The surface of the front paw image was linearly related to the body weight of the mouse suggesting that differences in this top feature could be secondary to differences in body weight, in particular for the 16p11.2 df/+ mice as they weigh significantly less than their controls (Fig 6B).

To investigate if the apparent differences in gait were due to increased speed, we plotted and analyzed stride duration as a function of speed (Fig 5A and 5B). Linear regressions after log-log transformations (to ensure linearity) were robust and there were significant differences in stride duration between for the WT and mutant mouse data. The differences, however, were driven mainly by extreme values (in particular, the two or three slower *df*/+ mice). This suggests that faster speed lead to a reduction in stride duration within each group and also that *df*/+ mice had slightly reduced stride length over and above speed effects. Similarly, we investigated if the differences in paw image were an indication of some underlying neurological deficit or secondary to a reduced body weight (as lower body weight translates onto less pressure exerted on the apparatus surface and reduced intensity and image). The scattergram of the front paw image area as a function of body weight shows that 16p11.2 *df*/+ mice are smaller and their paws produce a proportionally smaller image (Fig 5C and 5D). Regressions within each group were robust but there were no significant differences between the regression lines of the two groups showing that paw image differences reflect mainly body weight differences.

*Cntnap2* -/- mice showed significant differences overall, particularly in locomotor speed and gait (Fig 3C and 3D; Table 1). Investigation of gait features revealed that the length of the stride was not different, but the durations of the stride and stance were reduced as compared to the WT mice (Fig 4). Hind base width also appeared reduced (see S8 Table for statistics). The swing phase of a step was slightly longer at P60. The surface covered by the paws was not different, but the intensity of the paw image was very slightly reduced in the *Cntnap2* -/- mice. This pattern of results suggests that the overall discrimination relied on increased locomotor speed in the *Cntnap2* -/- mice and slightly less time in contact with the floor at P60. Indeed, a scattergram of the stride duration as a function of speed supported such a view: *Cntnap2* -/- mice were faster and their stride duration was proportionally shorter (Fig 5).

As before, to investigate if the apparent differences in gait were due to increased speed, we plotted and analyzed stride duration as a function of speed (Fig 5A and 5B). Linear regressions were robust and there were significant differences between for the WT and mutant mouse data especially at P60 (whereas, at P30, regressions depended on inclusion of the extreme slower and faster mice). Thus faster speed lead to a reduction in stride duration within each group, as in the deletion model. *Cntnap2* -/- mice had reduced stride length over and above speed effects at P60. Similarly, we investigate if the differences in paw image were secondary to reduced body weight (Fig 5C and 5D). Regressions within each group were again robust but there were no significant differences between the regression lines of the two groups showing again that paw image differences reflect body weight differences.

### Development

We describe in this section important milestones of development and health with associated behavioral measures. Before P7, behaviors and physiological measures cluster around three independent axes: motor (locomotion, rolling on a side, rearing, and jumping), thermoregulation and associated measures (baseline, post-test temperature and body weight), and anxiety-like responses (i.e., USVs and pivoting) [48]. Around P9, grooming appears as a fourth independent axis and, around P15, the thermoregulation and anxiety-like clusters fuse into one, coinciding with the maturation of the adrenal-pituitary axis and the appearance of a cold-stress endocrine response [49]. Given that the 16p11.2 model background is hybrid while the *Cntnap2* is pure inbred, we were not surprised to find that the above mentioned domains and their developmental time-course differed between genetic background strains. In addition to the behavioral battery described here, research assistants and animal care staff personnel handling the experimental subjects and breeders did not report any noteworthy behaviors through development and adulthood, such as seizures.

#### Body weight

**Fig 6:**
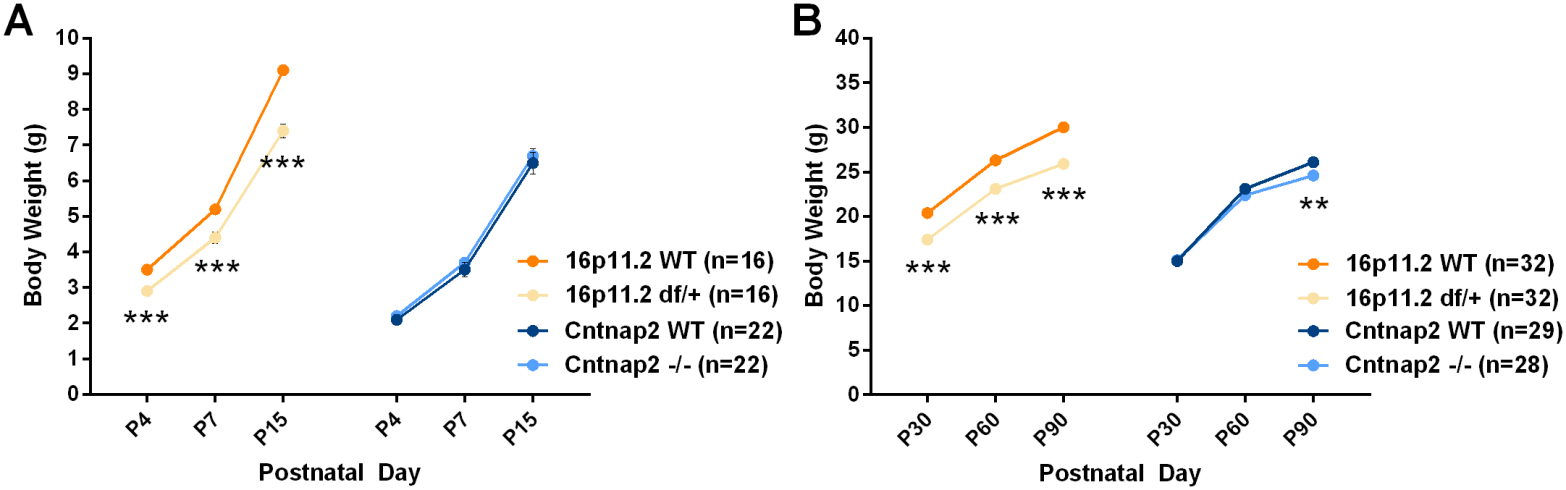
Body weight during neonatal (A) and postnatal (B) periods. Simple main effect analysis following a significant Genotype x Age interaction showed significant reductions as compared to corresponding WT controls. Note errors bars are hidden by the graphing symbols. Data shown are means ± SEM. (**p < .01, ***p < .001).

16p11.2 *df*/+ mice weighed less than corresponding WT mice and this difference became more pronounced with age (Fig 6 and S9 Table), and is consistent with other published reports [22, 23]. Adult but not neonate *Cntnap2* -/- mice gained weight at a slightly lower rate than WT mice, and were significantly lighter than WT mice at P90 (Fig 6 and S10 Table). The mixed 129/C57 background groups were overall heavier than the C57 groups.

#### Milk content

At an early age, milk content is a good indicator of the ability of the pups to nurse and therefore of their short-term health. Fewer 16p11.2 *df*/+ pups were seen with clear indication of milk content at P4, as compared to corresponding WT mice; however, these differences were gone by P7. For *Cntnap2* -/- mice, milk was visible at P4 and P7 and was not different from corresponding WT mice (S9 and S10 Tables).

#### Eye opening

16p11.2 WT and *df*/+ pups had both eyes opened at P13, regardless of genotype. In contrast, very few *Cntnap2* -/- mice had eyes open at this age, suggesting the C57 background is associated with slightly delayed development as compared to a mixed 129/C57 background (S9 and S10 Tables).

#### Ultrasonic vocalizations

**Fig 7:**
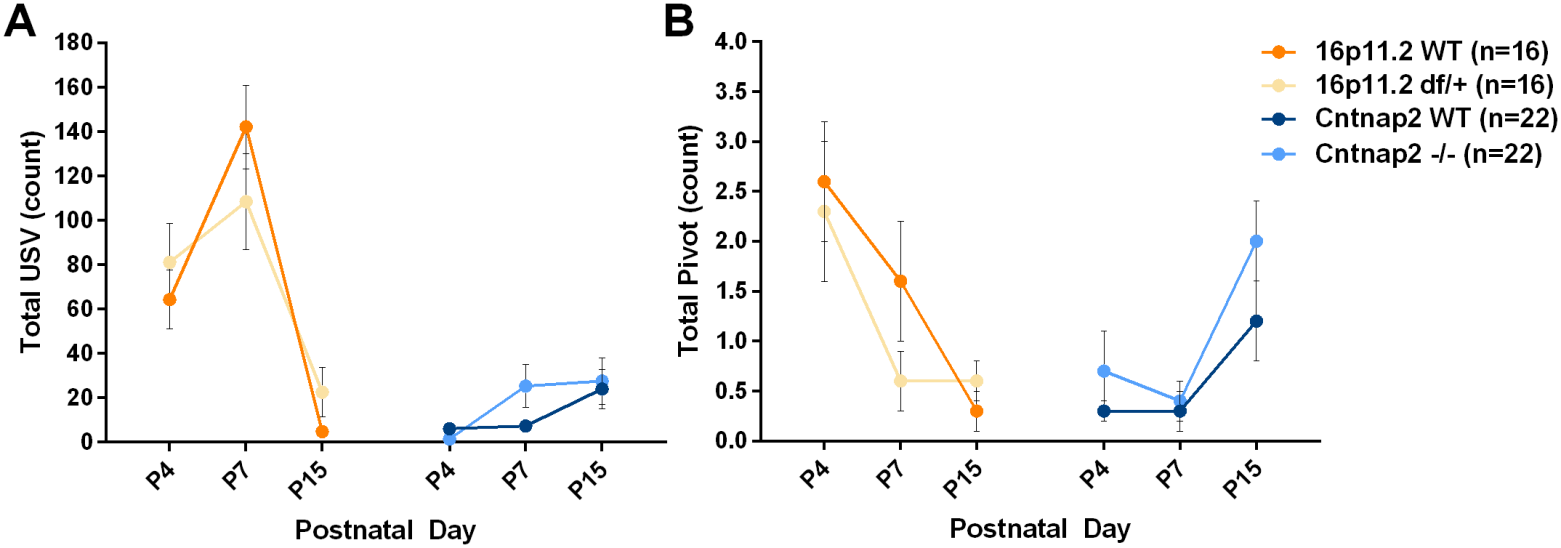
Isolation-induced vocalization and behavior. During the isolation test mice were separated from the dams and individually tested in a clean cage. A: Neonatal ultrasonic vocalizations (USVs) were more frequent in the 16p11.2 WT and df/+ mice than in the Cntnap2 WT and-/- mice, although there were no genotypic differences for either model; B: Pivoting, normally associated with the production of USVs, did not show genotypic differences either. Data shown are means ± SEM.

Pups’ ultrasonic vocalizations (USVs) likely serve a functional purpose and reflect negative affect during maternal isolation, and therefore are of interest for the assessment of normative (social) development [48]. Although it was initially proposed that the calls were part of a thermogenic strategy [50], there is no doubt that there is a strong social and emotional component [51–53]. There were very large differences between the two genetic backgrounds of the two different mouse models, although there were no genotypic differences (Fig 7A; S11 and S12 Tables). Mice in the mixed C57/129 background (16p11.2 *df*/+ and WT) showed an expected increase in vocalizations up to P7, and then a decrease [48], whereas the C57 background strain (*Cntnap2* -/- and WT) was associated with very low call frequency at all ages, although it increased from P4 to P15.

#### Activity

Locomotion significantly increased with age in both models, modulated by genetic background but not by genotype (S5 Fig; S11 and S12 Tables). Pivoting is associated with the number of ultrasonic calls, as it may serve to help to propagate the sound in different directions [48]. Despite the close association of USVs with pivoting, the time courses in 16p11.2 *df*/+ and WT mice differed: pivoting was maximal at P4 and decreased with age, whereas the number of ultrasonic vocalizations peaked at P7. *Cntnap2* -/- and WT mice showed increased pivoting as a function of age (Fig 7B and S12 Table). Rearing is used as a measure of general activity and, sometimes, exploration. Rearing and grooming were not observed until P15, when pups are more mobile and have better motor coordination. Neither measure showed any genotype effect.

In addition to activity, we measured different aspects of motor coordination, since deficits in this domain may be early signs of autism [54] and have been used in the characterization of related animal models [55].

The geotaxis (or gravitaxis) test measures motor coordination and the geotactic reflex in response to gravitational pull and occurs only during the first week of development. Young pups attempt to turn to an upward position when placed downward on a slope while older pups develop more mature behaviors such as walking sideways or downwards, or jumping. 16p11.2 *df*/+ mice completed marginally fewer turns than their WT control mice at P7 (*p* < .051), because they tended to show no behavioral reaction, but not because they fell more frequently (Table 2 and S13 Table). At P15, *Cntnap2* -/- mice walked down more than WT mice suggesting a mature response and maybe higher activity levels (Table 2 and S14 Table).

**Table 2:** The Geotaxis Test for the Two Models at the Three Ages Studied.

	16p11.2 WT			16p11.2 *df*/+			*Cntnap2* WT			*Cntanp2* -/-		
Age	P4	P7	P15	P4	P7	P15	P4	P7	P15	P4	P7	P15
**Percent falls**	12.5	0.0	12.5	18.8	3.1	6.3	25.0	11.4	4.5	30.4	21.7	0.0
**Percent turns**	43.8	56.3	18.8	25.0	21.9	18.8	6.8	9.1	72.7	15.2	10.9	56.8
**Percent walk down**	0.0	0.0	68.8	0.0	0.0	71.9	0.0	0.0	13.6	0.0	2.2	43.2
**Percent no reaction**	43.8	43.8	0.0	56.3	75.0	3.1	68.2	79.5	9.1	54.3	65.2	0.0

Mice were placed on an inclined plane and their behavior was observed. A fall indicates lack of coordination whereas a turn indicates a geotactic reflex response. After P7 this reflex is lost and other behaviors appear, such as walking down the plane or sideways.

**Fig 8:**
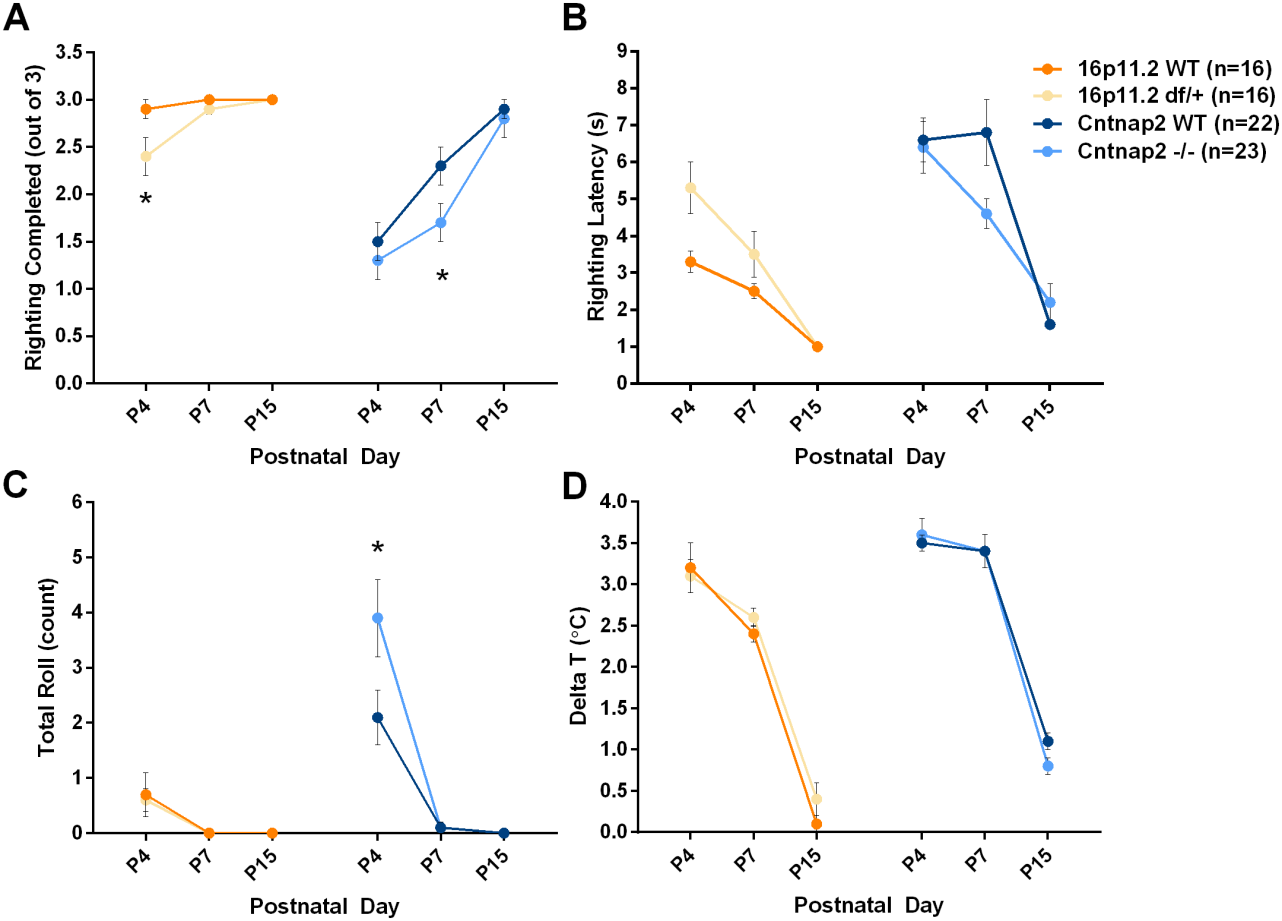
Motor coordination and thermoregulation during the neonatal test. In the righting test mice are placed upside down three times and allowed to right until they are on all four limbs. During the isolation test the number of times mice fall on their sides or roll is used as another measure of motor coordination. Thermoregulation is measured by taking axillary temperature before and after the isolation test. A: Number of righting successes; B: righting latency; C: Number of rolls on a side; D: Difference between the axillary temperature measurements before and after the isolation test. Data shown are means ± SEM. (*p < .05).

Righting is a measure of motor coordination and a response to proprioception. There were notable differences between the two genetic backgrounds, with the mixed genetic background showing a more mature response (more frequent and faster righting) than the C57 strain (Fig 8A and 8B). 16p11.2 *df*/+ mice righted themselves significantly fewer times than the WT mice at P4 and took longer to right than WT mice at P4 and P7. *Cntnap2* -/- mice righted themselves significantly fewer times but, when they did so, were faster than WT mice at P7. During the isolation test, as pups walk around, they occasionally roll onto their side, a behavior possibly indicative of weak motor coordination, although it could be secondary to higher activity levels [48]. Here we only observed rolling at P4 (S13 and S14 Tables). There were no significant differences between 16p11.2 *df*/+ and WT mice. *Cntnap2* -/- pups rolled significantly more than WT control pups (Fig 8C).

#### Thermoregulation

This is a measure of homeostatic regulation, which is weak during the first two postnatal weeks until animals become homoeothermic around P14 [49]. Pups lose temperature rapidly during the 3–4 minute period in which they are handled and isolated for behavioral observations. There were no significant differences between genotypes for either model (Fig 8D and S5 Fig; S9 and S10 Tables). The pure C57 mice lost more heat than the mixed background pups.

### Tests of Social Behavior

#### Three-chamber test

**Fig 9:**
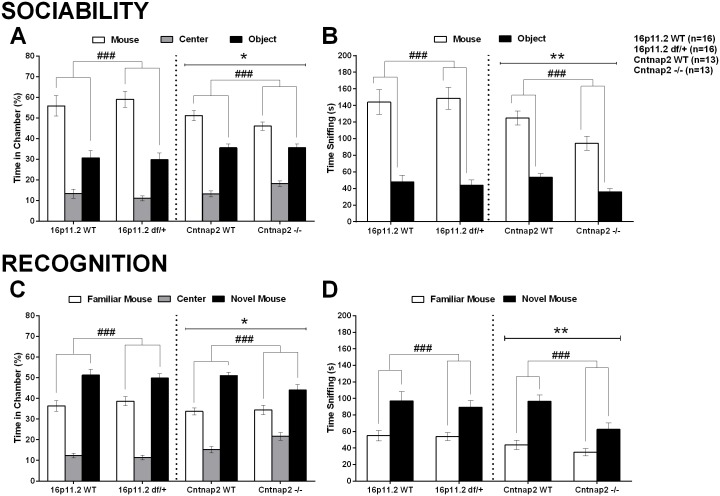
Mouse preference for a social stimulus versus a non-social stimulus (sociability) and recognition of a social stimulus in the three-chamber test. A: During the sociability phase the percent time spent in each of the three chambers showed that all groups spent more time in the social chamber as shown by a significant Chamber Type main effect (in a 2-way mixed model Genotype x Chamber Type ANOVA). The Cntnap2 -/- mice spent less time in the side chambers compared to the WT control mice as shown by a significant Genotype main effect (with no significant Genotype x Chamber Type interaction). B: During the sociability phase all groups sniffed more the social container as shown by a significant Stimulus Type main effect (in a 2-way mixed model Genotype x Stimulus Type ANOVA). The Cntnap2 -/- mice spent overall less time sniffing than the WT control mice as shown by a significant Genotype main effect (with no significant Genotype x Stimulus Type interaction). C: During the recognition phase the percent time spent in each of the three chambers showed that all groups spent more time in the novel mouse chamber than in the familiar mouse chamber. The Cntnap2 -/- mice again spent less time in the side chambers compared to the WT control mice (analysis as in A). D. During the recognition phase all groups sniffed more the novel social container. The Cntnap2 -/- mice again spent overall less time sniffing than the WT control mice (analysis as in B). Time in each chamber was recorded automatically, whereas sniffing time was scored manually Data shown are means ± SEM. (Chamber or Stimulus Type main effect: ^###^p < .001; Genotype main effect: *p < .05, **p < .01).

**Fig 10:**
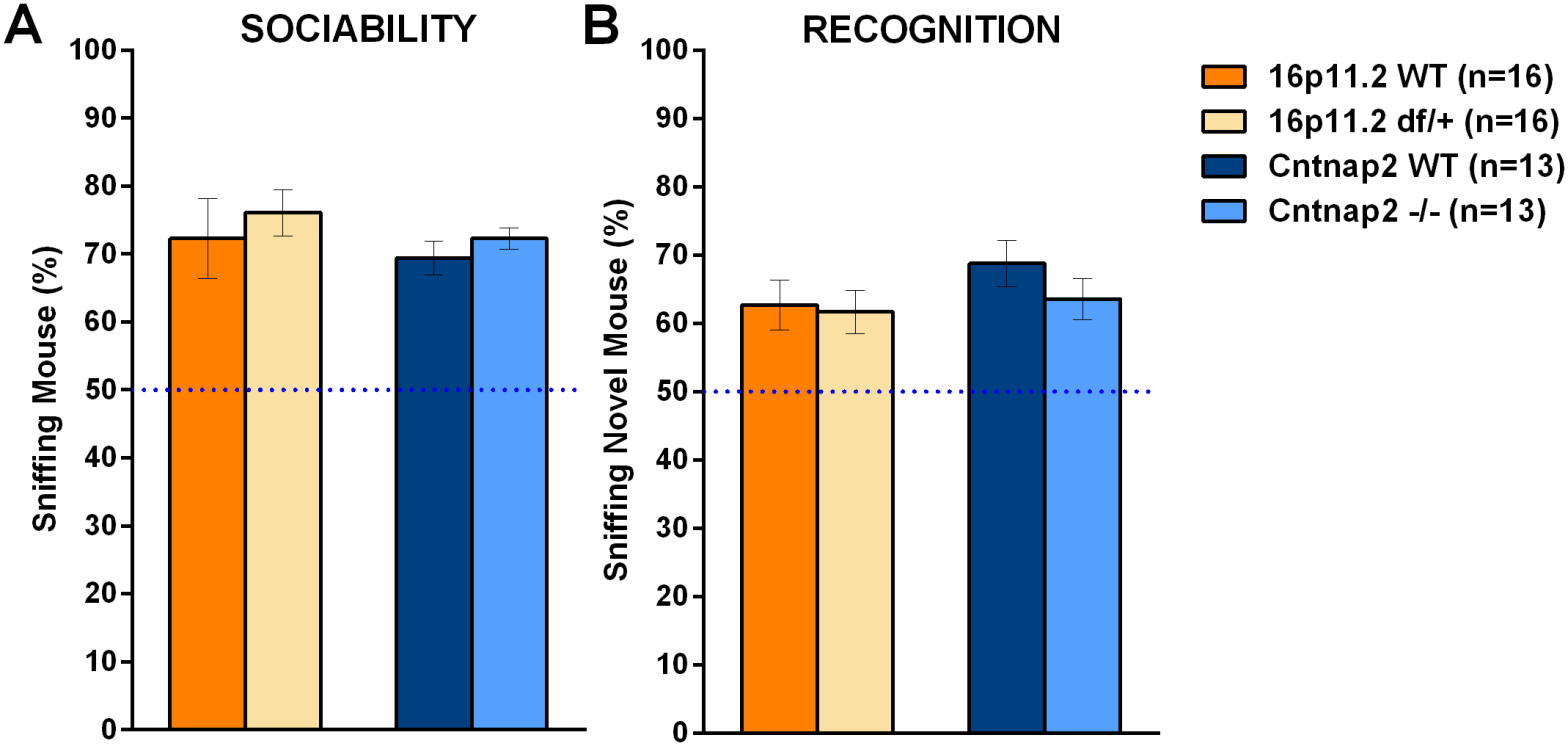
Proportion of total time sniffing a stimulus in the three-chamber social test. In the three-chamber social test the proportion of the total time sniffing the novel mouse or an alternative (social or not) can be used as a measure of preference, with a dashed line at 50% indicating a lack of preference. A: All groups showed a strong preference for the social stimulus without any genotype effects; B: All groups showed a strong preference for the novel social stimulus, with no genotype effects. Sniffing was scored manually. Data shown are means ± SEM.

The three-chamber test is a widely-used behavioral test to assess sociability and social recognition [56]. Overall, in the present experiments, neither the 16p11.2 *df*/+ nor the *Cntnap2* -/- mice showed clear social preference deficits in either phases of the test. While the 16p11.2 *df*/+ results are consistent with published reports [22], our results with the *Cntnap2* -/- mice differ from published reports [24] as they rather point to differences in the response to novel stimuli, which are not necessarily or exclusively social. Experimental differences can account for or contribute to these discrepancies. During the initial habituation testing phase, both the 16p11.2 *df*/+ and their WT controls explored one of the side chambers slightly more than the other (S6 Fig; see S15 Table for all statistics), but to a similar extent in both genotypes. During the sociability testing phase (presenting a choice between a mouse versus an object), the 16p11.2 *df*/+ mice did not show social deficits. That is, 16p11.2 *df*/+ and WT mice spend more time in the social chamber than in the object chamber and sniffed the cup with an unfamiliar mouse longer than the object cup to a equivalent degree (Figs 9A and 9B, 10A, and S6 Fig). Entries into the side chambers did not differ across stimuli or genotypes (S6 Fig). During the 3-chamber social recognition phase (presenting a choice between a familiar and a novel mouse) the 16p11.2 *df*/+ mice did not show deficits either. 16p11.2 *df*/+ and WT mice spent more time in the novel mouse chamber than in the familiar mouse chamber and sniffed the novel mouse longer than the familiar mouse to an equivalent extent (Figs 9C and 9D, 10B, and S6 Fig). Entries into the side chambers did not differ across stimuli or genotypes (S6 Fig).

Although *Cntnap2* -/- mice displayed normal social behavior, they showed a clear difference in the pattern of exploration of the apparatus both during the non-social and social phases of the test. During the habituation phase, *Cntnap2* -/- mice explored the side chambers less than the WT mice (S6 Fig; see S16 Table for all statistics). During the sociability phase, *Cntnap2* -/- mice again spent less time than the WT mice in both side chambers, and sniffed the two stimulus cups less. However, both *Cntnap2* -/- and WT mice spent relatively more time in the social chamber than in the object chamber and sniffed the mouse container longer than the object container, with proportionally equivalent social preference (Figs 9A and 9B, 10A, and S6 Fig). Entries into the side chambers did not differ across genotypes, although both groups crossed more towards the mouse chamber, suggesting mutant mice did not stay longer in the center simply due to inactivity (S6 Fig). In the social recognition phase *Cntnap2* -/- mice again spent less time than the WT mice in the two side chambers. Both genotypes preferred to spend more time in the chamber with the novel mouse, and to sniff its container. The *Cntnap2* -/- showed a non significant slight reduction in preference but none of the Genotype x Stimulus interactions reached significance (Figs 9C and 9D, 10B and S6 Fig). Entries into the side chambers did not differ across genotypes either, although both groups crossed more towards the novel mouse chamber, suggesting mutant mice did not stay longer in the center due to inactivity (S6 Fig). The genotype differences seen in this test are better described as variations in the exploration and activity patterns, with secondary effects of social behavior, as the exploration pattern of the *Cntnap2* -/- in the 3-chamber apparatus was already significantly different during the habituation phase, when no social stimuli were present. Hyper- or hypo-activity did not seem to be a confounding factor as there were no significant genotypic differences in chamber crossings for either model (S6 Fig).

#### Reciprocal social interaction

**Fig 11:**
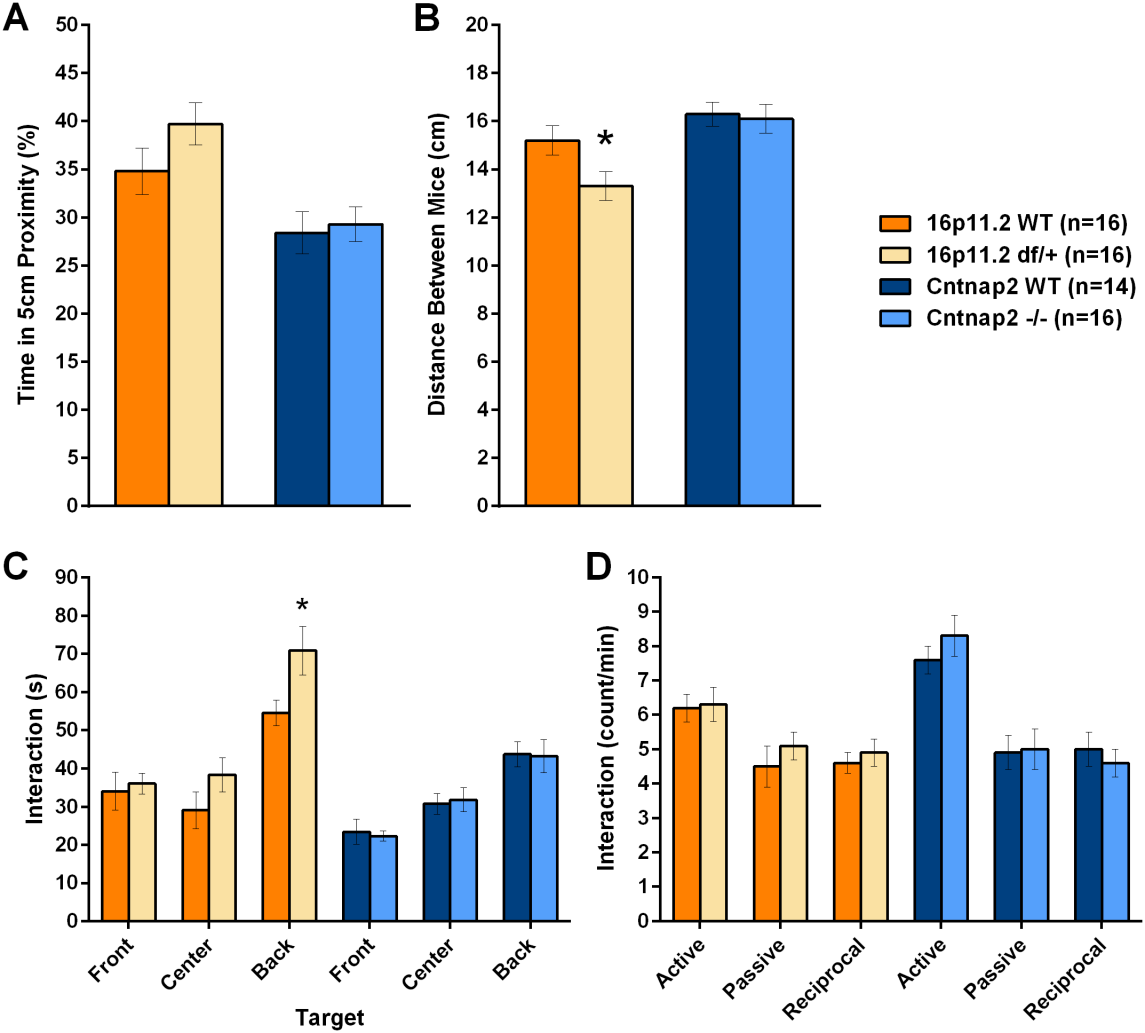
The reciprocal social interaction test did not show deficits in social behavior in either model. A: The time in which the mice were closer than 5 cm was similar for the two mutants as compared to their respective WT controls. All behaviors with the exception of the active, passive and reciprocal interactions were recorded using an automated system. B: During social interactions, 16p11.2 df/+ pairs were closer to each other as compared to the WT control pairs, whereas Cntnap2 -/- pairs did not show a difference compared to their corresponding WT control pairs; C: Number of interactions of the nose of a mouse towards the front, side or back of the paired mouse. 16p11.2 df/+ mice showed a slight increase in the number of interactions, but only for the interactions towards the back. Cntnap2 -/- mice were similar to their control WT mice; D: Active, passive and reciprocal interactions were similar between mutants and their respective controls. Data shown are means ± SEM. (*p < .05).

In the reciprocal social interaction assay, in which pairs of genotype- and age-matched mice are allowed to freely interact, neither 16p11.2 *df*/+ nor *Cntnap2* -/- mice showed social interaction deficits. 16p11.2 *df*/+ pairs maintained a shorter distance between each other than WT-WT pairs (Fig 11A; see all statistical results in S17 Table). 16p11.2 *df*/+ pairs were in close proximity and engaged in active, passive and reciprocal social behaviors for as long as the WT-WT pairs but remained in nose-to-back interactions for longer than WT pairs (Fig 11B and 11D). *Cntnap2* -/- pairs maintained a distance between each other similar to that of WT-WT pairs (Fig 11A; see all statistical results in S19 Table). The durations and rate of all types of interactions showed no effect of Genotype (Fig 11B and 11D).

Ultrasonic vocalizations were very rare during testing and did not differ by genetic background or genotype. An alternative design, in which mutant mice of both models were paired an unfamiliar WT mouse from each colony rather than with another mutant, was also used, and similarly did not uncover social interaction deficits (see S7 and S8 Figs; S18 and S20 Tables).

#### Urine-exposure open field

We used the open field test to measure scent marking and ultrasonic vocalizations of male mice in response to the scent of urine from an estrous female. Scent-marking is a response of males to attract females, used to demarcate territories and requires no social experience [57, 58]. Ultrasonic vocalizations, on the other hand, are particularly sensitive to the social context as they are elicited by the combination of social experience with females and female scent exposure. This test and associated responses are, therefore, a valuable tool to investigate the social behavior of murine models of disease [59] and expand the characterization of the 16p11.2 *df*/+ and *Cntnap2* -/- models. Overall, 16p11.2 *df*/+ mice responded to the urine exposure similarly to WT control mice; whereas *Cntnap2* -/- mice showed reduced vocalizations in response to the female urine stimulus.

**Fig 12:**
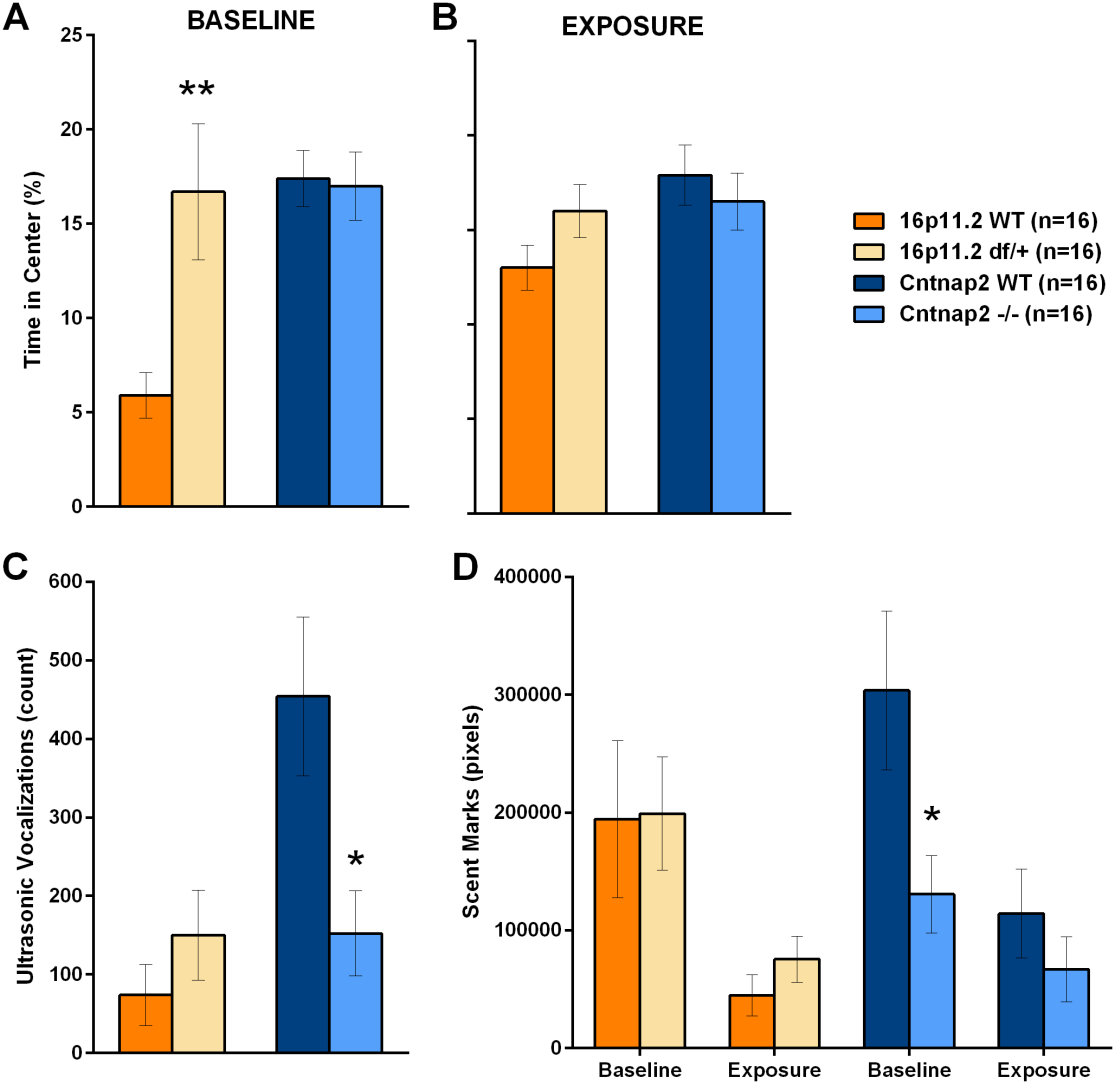
Activity, vocalizations and marking in the urine-exposure open field. Female-experienced male mice are first habituated to an arena and then presented with a small quantity of urine from a female in estrous. A: During the baseline session 16p11.2 df/+ mice explored the center of the arena more than the WT mice, whereas Cntnap2 -/- showed no differences from its WT control; B: during the exposure session all groups explored the center similarly; C: Ultrasonic vocalizations emitted during the exposure session showed deficits in the Cntnap2 -/- mice but not in the 16p11.2 df/+ as compared to their WT controls; D: Similarly, Cntnap2 -/-, but not 16p11.2 df/+ mice, showed deficits in scent marking during both baseline and urine exposure. Data shown are means ± SEM. (*p < .05; **p < .01).

In particular, during the baseline session, 16p11.2 *df*/+ mice did not differ from their WT controls in total distance travelled, either in the whole arena or in the center, although they spent more time in the center of the chamber (Fig 12A; see all statistics in S21 Table). During the exposure session in which urine from an estrus female is placed in the center of the chamber, 16p11.2 *df*/+ mice covered more distance overall, but did not seem to prefer the center (Fig 12B). 16p11.2 *df*/+ and WT mice vocalized and scent-marked to the same extent (Fig 12C and 12D).

During the baseline session, *Cntnap2* -/- mice did not differ from their WT controls in total distance travelled or time spent in the center of the chamber (Fig 12A; see all statistics in S22 Table). During the exposure session, however, they were hyperactive, although they spent the same time in the center as WT mice (Fig 12B). *Cntnap2* -/- mice showed significantly less scent-marking during the baseline phase and vocalized less than WT mice during the exposure phase (Fig 12C and 12D), suggestive of deficits in response to an olfactory social stimulus, to pre-exposure to a female, or a decreased territorial response.

### Cognition

#### Procedural T-maze

**Fig 13:**
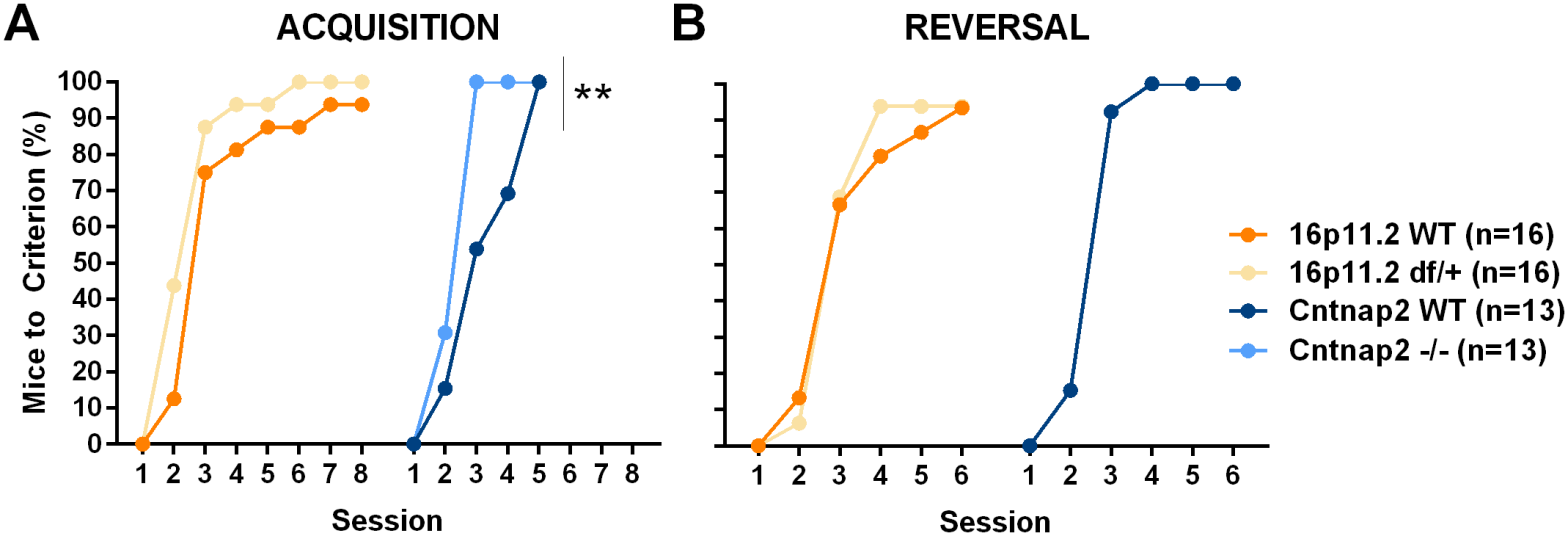
Acquisition and reversal in the procedural T-maze. In the procedural T-maze mice are trained to reach a platform on one side of the maze. After they perform correctly 6 out of 8 trials in two consecutive days, the platform position is reversed and mice are trained under the new rule. A: The proportion of mice that reached criteria during the acquisition (maximum 8 days) shows that there were no deficits in the 16p11.2 df/+ mice but Cntnap2 -/- mice learned the task faster; B: during reversal (6 days maximum) there were no deficits either. Note the reversal data for Cntnap2 WT mice are identical to that of the Cntnap2 -/- mice. Data shown are proportions of mice that started the task (Genotype Factor, Survival Analysis, Chi Square: **p < .01).

The procedural T-maze measures the ability of an experimental subject to learn a rule focusing on “what needs to be done” rather than “where one should go”. The reversal phase assesses behavioral flexibility, as animals need to inhibit a preponderant response to accurately perform under a new rule. Versions of this task have been used to explore perseveration and the role of cortico-striatal and hippocampal circuits [60, 61]. 16p11.2 *df*/+ and their corresponding WT mice learned the procedural rule with the same speed during the initial acquisition and reversal, and showed therefore no deficit, whereas *Cntnap2* -/- mice acquired the tasks significantly faster than the WT control mice although performed normally during reversal (Fig 13 and S9 Fig; see S23 and S24 Tables for all statistics).

### Sensory-Motor Gating

#### Prepulse inhibition of startle

**Fig 14:**
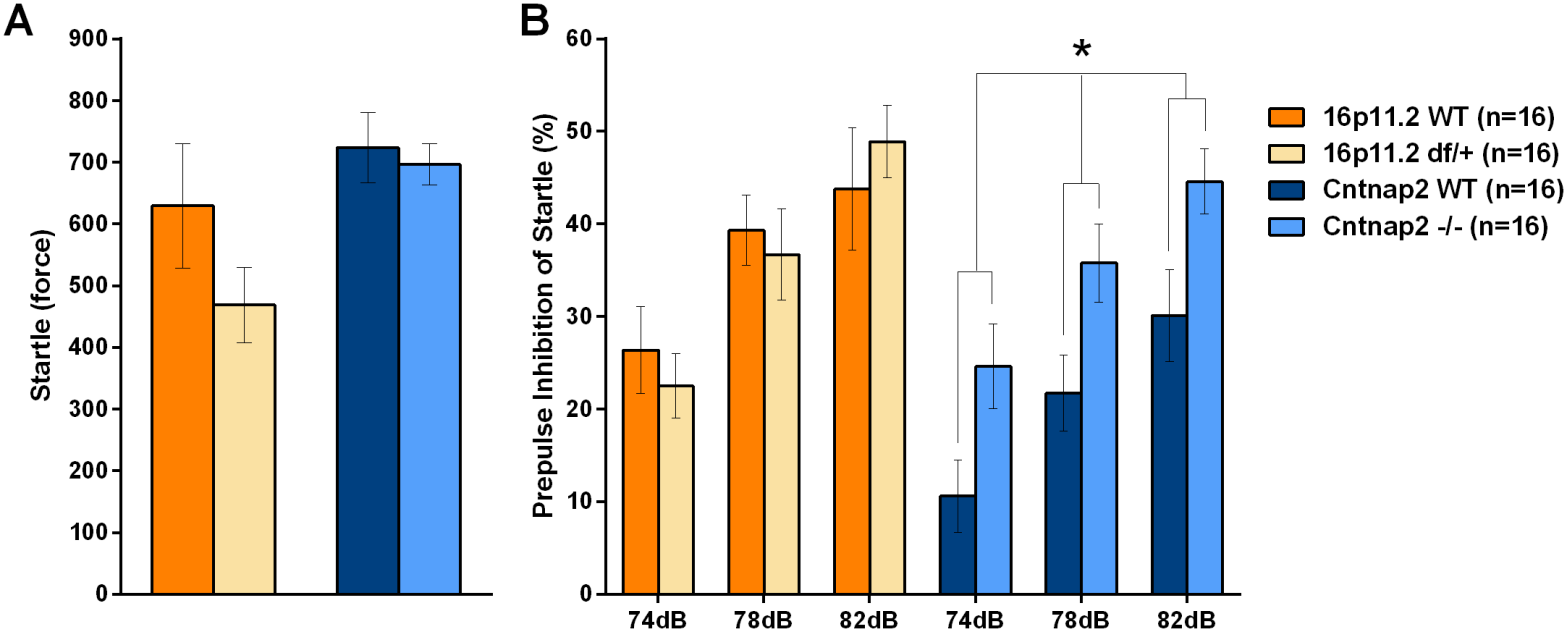
Prepulse inhibition of startle in the two models. PPI reflects the % decrease in startle to a sound when preceded by a quieter pre-pulse sound. A: Both mutant groups showed startle responses (arbitrary unit) similar to those of their WT control mice; B: Cntnap2 -/- but not 16p11.2 mice showed stronger prepulse inhibition of startle than the corresponding WT control mice. Data shown are means ± SEM. (*p < .05).

The startle response is a basic behavioral reaction to a strong auditory or tactile stimulus. The prepulse inhibition of startle (PPI) describes a phenomenon by which a non-startling sound preceding the startling sound by a few milliseconds reduces the amplitude of the startle response. This test has been used to study pre-attentive processes, a hallmark of schizophrenia [62–64]. 16p11.2 *df*/+ mice did not show differences from their WT controls during the startle test or PPI. *Cntnap2* -/- mice did not differ in their startle response; however, they showed significantly higher PPI than their WT controls (Fig 14; S25 and S26 Tables).

### Repetitive/Anxiety-like Behavior

#### Marble burying

**Fig 15:**
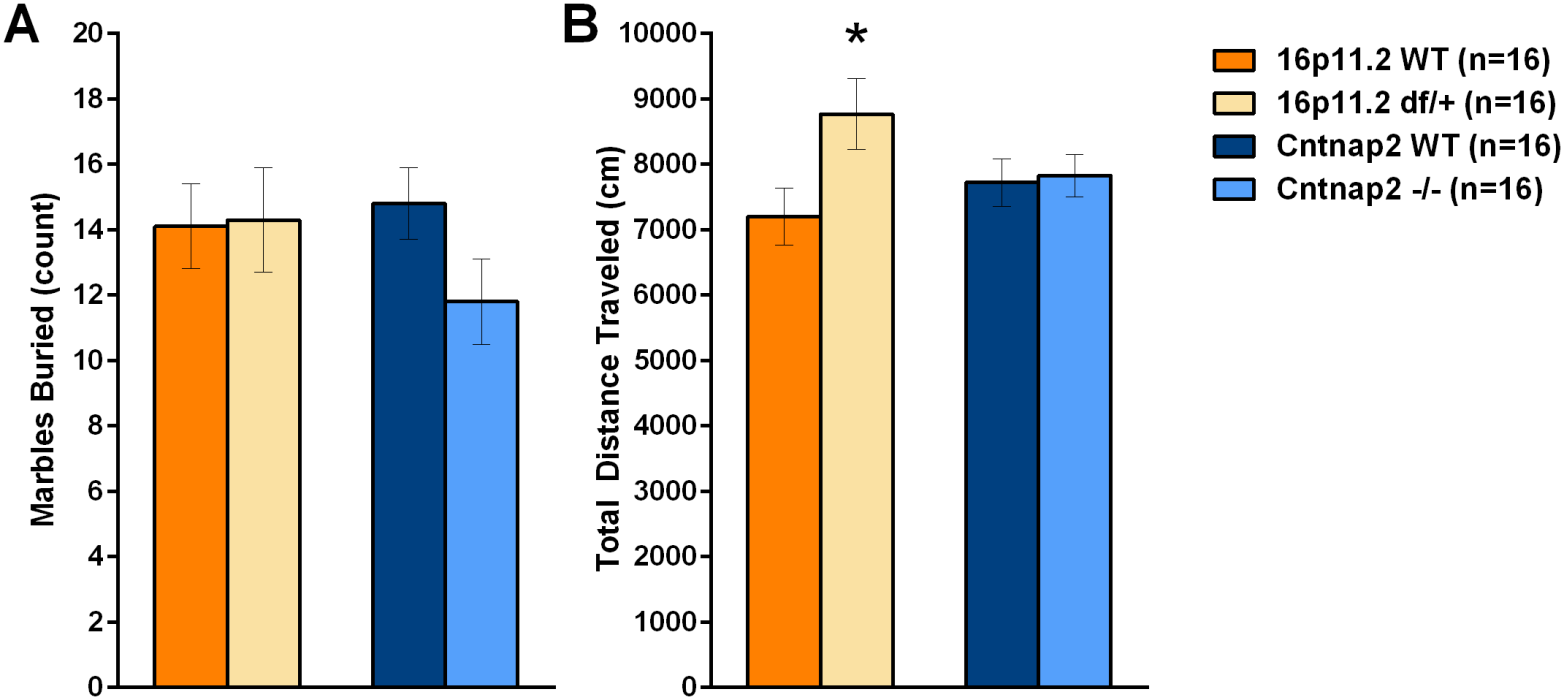
Number of marbles and activity in the marble burying test. In the marble burying test mice are presented with a novel diggable medium. Marbles are placed on the surface to help quantify the amount of digging shown by the mice. A: Number of marbles buried was not different between the mutants and their respective WT controls; B: Locomotor activity in 16p11.2 df/+ mice was greater than that WT control mice, whereas the Cntnap2 -/- mice were as active as the corresponding WT mice. Data shown are means ± SEM. (*p < .05).

The robust response of rodents to a novel “diggable” medium is sensitive to pharmacological profile, making it a common assay for the assessment of anxiety, stereotypic and/or obsessive-compulsive-like behavior [65, 66]. The test has been frequently used to characterize mouse models of autism [67]. 16p11.2 *df*/+ mice buried an equivalent number of marbles as compared to WT controls and were hyperactive, whereas *Cntnap2* -/- mice did not differ from its corresponding control in either measure (Fig 15; S27 and S28 Tables).

### Inter-Individual Variability

Because individuals with a 16p11.2 deletion show wide phenotypic variation [28–31], we assessed if there was more variable behavioral expression in the 16p11.2 *df*/+ (or in the *Cntnap2* -/-) mice than in their corresponding WT mice. We first rescaled all data for proper comparison across tests taking z-scores for WT and mutant mice together, separately for each model. Thus, for each mouse, we calculated z-scores for each of the many behavioral endpoint measures or features. We hypothesized that mice that have extreme traits will tend to be outliers for many features across many tests. The average z-scores for each subject will then reflect their extreme traits. Also, to explore heterogeneity due to incomplete penetrance, we proposed that, if within each group there are mice which present with almost normal traits and others with extreme traits, then the mutant group z-score variability will be larger. We therefore took the average z-score for each mouse (ignoring its sign) across features and explored genotype effects (see Materials and Methods). The two mouse mutant groups showed z-scores group averages and standard deviations almost identical to those of the corresponding WT control groups (S10 Fig), showing no signs of increased variability in behavioral traits in any group.

## Summary and Discussion

As part of a larger project encompassing the study of several mutant models of autism, we have compared two models using a comprehensive battery covering autism-related behavioral domains and health. Although inter- and intra-laboratory replicability issues need to be seriously considered in any behavioral project, we did not set up to replicate the exact procedures used in previous publications, but rather to establish a comparative database in which similarities and commonalities among models can be explored. We sought to address inter-laboratory replicability by having all models phenotyped by the same lab and will address intra-lab reproducibility by replicating the most robust findings from the five models. Analysis of all our findings will perhaps identify behavioral commonalities across etiologies.

Overall, we found that the 16p11.2 *df*/+ model had a mild phenotype that included decreased anxiety-like features and increased activity without changes in social behavior. Interestingly, *Cntnap2* -/- mice also showed hyperactivity and possibly reduced anxiety. They also showed some deficits in response to a social stimulus (urine) although we did not replicate published social deficits in the 3-chamber or the social interaction tests. It should be noted that our standard practice is to provide enrichment to all mice, which could have beneficial effects on its own. Although we could not fully compare husbandry conditions with the originating labs, mice in our study were housed in a more enriched environment, a variable that should be further investigated in follow up studies. It is possible that our enriched housing explains why we observed milder phenotypes than those reported elsewhere. Cage enrichment is an important experimental factor that is well known to influence higher order behaviors such as social cognition and potentially ameliorate deficits in animal models of disease [68, 69]. Enrichment, however, has not been shown to reduce the reproducibility of experimental results [70].

In our assessments of health and development, 16p11.2 *df*/+ mice showed reduced body weight compared to the WT mice as neonates, a difference that grew larger with age. We did not find significant differences in milk content contrary to Horev et al.’s findings in a different mixed strain (50% C57BL/6N and 50% 129Sv) [22]. We used ultrasonic vocalizations as a way to assess the pups' sensitivity to social isolation and thermal challenge. We did not replicate the published abnormal neonatal ultrasonic vocalizations, although we did not pursue a spectral analysis. USVs do modulate the dam’s retrieval response in some situations but they do not consistently do so [71, 72] and therefore caution is needed when interpreting phenotypic differences in USVs in terms of communication intent, success or failure. We observed a robust number of calls in the 16p11.2 mixed background strain demonstrating that our experimental protocol was suitable for a comparison between the 16p11.2 *df*/+ mice and its control. However, the same protocol elicited few calls in the C57 background suggesting that there was not sufficient dynamic range to look for reduced vocalizations in the *Cntnap2* -/- mice. In addition to genotype differences we observed large genetic background effects. In general, the mixed 129/C57 background mice seem to be more mature than the C57, with earlier eye opening, better motor coordination, and heavier body weights. This may be related to hybrid vigor.

16p11.2 *df*/+ mice showed minor signs of motor incoordination as neonates, but otherwise were similar to their WT controls in attaining developmental milestones. In the NeuroCube test of gait and locomotion, 16p11.2 *df*/+ adults showed significant differences in several features, some possibly due to decreased body weight. *Cntnap2* -/- neonates tended to roll on their sides more often and righted themselves less often. They, however, performed faster in a geotaxis test. This improved performance in the latter test resembles published results showing better rotarod performance in the adults, faster reactions to pain stimulation, and faster completion of an olfactory task [24]. During NeuroCube testing as adults, *Cntnap2* -/- mice showed significant differences in speed and gait, as compared to the WT mice. The features showing a genotype effect seem correlated to the increased velocity of the mutants, although velocity may not account for all the changes in gait features.

SmartCube showed that 16p11.2 *df*/+ mice had a signature likely driven by their higher activity levels. The *Cntnap2* -/- mice were also more active and showed possibly reduced motor competence, although it remains to be tested if this is related to the heightened locomotor activity. We did not find evidence of an anxious phenotype as both models also showed reduced latencies to approach an aversive stimulus and reduced freezing. These results were corroborated by data from the marble-burying test. 16p11.2 *df*/+ mice were no different from WT controls in the number of marbles they buried although heterozygous mutants were hyperactive. For the *Cntnap2* -/- mice, there were no observed changes in marble burying or activity.

*Cntnap2* -/- mice did not display increased grooming during the SmartCube behavioral test or in the vivarium (i.e., lack of noticeable behavior or barbering patterns). Similar to the 16p11.2 *df*/+ deletion model, and in agreement with the published phenotype, the *Cntnap2* mutants were hyperactive in several tests. As reported, we did not find evidence of heightened anxiety-like behavior, and we did not observe seizures during behavioral testing. Although we observed social deficits in one test (urine-open field), we failed to find differences in the 3-chamber and reciprocal social interaction test. These results are somehow at odds with the published literature [24], and could be (partially) explained by procedural differences, such as housing in an enriched environment, among labs. In fact, in another reciprocal interaction study in which *Cntnap2* -/- mice were tested during their subjective night, we found no differences in interaction durations or bouts, although mutants were more active and, perhaps as a consequence, followed the paired mouse more than control mice (not shown). Whereas there were no differences in the three-chamber test, 16p11.2 *df*/+ were closer to and interacted more with a partner in the reciprocal social interaction test. In the urine-open field test 16p11.2 mutants spent more time in the center, during the baseline session, but vocalized and scent-marked normally. Based on three social tests, the three-chamber social interaction and two versions of the reciprocal social interaction test, we failed to find clear social deficits in either mutant model. This is in contrast with published results showing decreased interaction time and beneficial effects of oxytocin in the *Cntnap2* model [73, 74].

On the other hand, we observed a decrease in the number of ultrasonic vocalizations in the *Cntnap2* -/- mice during the urine-exposure open field test, consistent with the view that this model exhibits social deficits. This result should be interpreted with some caution, however, as the effect size for this highly variable measure was low and, when these animals were tested during their subjective night in a different study, this deficit was no longer present (not shown). In the 3-chamber test, *Cntnap2* -/- mutant mice showed a different pattern of exploration during the non-social habituation session and later on, during the social choice phase. Indeed, *Cntnap2* -/- mice explored the side chambers of the 3-chambered apparatus less, despite the different stimuli present. Thus, while they explored the sides less when two mice had been placed there (social recognition phase), suggesting a social deficit, they also explored less the sides when they were empty (habituation phase). Activity did not seem to confound these results as entries into the side chambers were not significantly different across genotypes. In the urine-exposure open field test, 16p11.2 *df*/+ mice, instead, did not show differences as compared to the WT control mice. This is somehow at odds with published literature showing that this model, in a different genetic background, has a significantly decreased response to female mice [75]. However, this paper also showed that such 16p11 *df*/+ mice have robust sensory deficits, exemplified by the lack of startle response. This in contrast with the normal startle response presented here, suggesting major differences between the two models in these different genetic backgrounds [76].

In a test of cognition and behavioral flexibility, 16p11.2 *df*/+ showed normal acquisition and reversal in a procedural T-maze. Although cognitive deficits in a fear conditioning task, reversible by a mGluR5 antagonist, have been described [77], it is possible that the differing neural circuitry that drive the two tasks are responsible for the discrepancy. *Cntnap2* -/- mice, instead, showed faster acquisition with normal reversal. Their improved performance resembles the reported improved performance in the rotarod, and suggests that tests of arousal may be of interest. Although it has been reported that this model has a deficit in the reversal of a spatial learning and memory task [24] our results do not support a general “resistance to change” phenotype.

In short, the 16p11.2 *df*/+ mice showed increased activity and decreased body weight, confirming published reports in this same model and in a different 16p11.2 deletion model, established on a different genetic background [23]. One of the core domains of autism, social behavior, was not affected, in agreement with previous reports despite the use of different background strains [22]. We did not observe increased repetitive behavior in the tests included in our battery. Another result consistent with previous reports is a marginally significant birth ratio biased against the mutant mice in this mixed background (with an average 50% C57BL/6J and 50% 129S1/SvImJ), which implies some variability in the penetrance of the genetic insult, during either pre- or postnatal development. A deficiency in the sperm ability to fertilize the egg in vivo and a progressive loss of transmission with each generation of backcrossing to C57B6/J may explain this bias. However, the success of in vitro fertilization using C57B6/J eggs argues against sperm-specific deficits such as motility (Cathleen Lutz, Jackson Laboratories, personal communication). The dependency of these deficits on the background strain is further shown by Horev et al.’s findings showing some postnatal lethality in a C57B6/N x 129S7/SvEvBrd-Hprt1b-m2 F1 background [22] but successful transmission without significant lethality on a C57B6/N pure background (Mills, personal communication).

We found no evidence of increased variability in behavioral traits in any group. The lack of increase variability is consistent with the bulk of the data collected, where the mutant groups did not seem to be particularly more variable than the control groups. It possible that genetic backgrounds with little genetic variability, such as those used here, may not be the most appropriate to analyze incomplete penetrance predictions for these mutations.

The goal of this study was to conduct a thorough behavioral characterization of mouse models of autism with construct validity in order to find a robust phenotype. We took a broad approach in hopes of identifying quantitative, replicable behaviors that could be useful for drug development. Whether a model exhibits a strong or weak behavioral phenotype may affect its usefulness, but not its construct validity.

We believe that studying mouse models with robust construct validity is imperative, as they carry mutations that are found in humans with autism, even if these mice do not necessarily phenocopy human autism symptoms. Indeed, we did not expect the mouse models to recapitulate all of the autism domain deficits (to the extent that that is possible in a lower species); rather, we hoped to discover robust and consistent behaviors that may be useful for distinguishing the effects of treatment in future studies. Our study shows that these behavioral phenotypes are quite complex has although it identified hyperactivity as a common phenotype in both models.

We would also like to stress that we hope our comprehensive battery provides the scientific community with informative data beyond just its major conclusions. Negative results can sometimes be as informative as positive results. For example, while we found only subtle developmental differences in these models, this information might be very useful for other researchers in interpreting results from their own labs when using these mice.

## Materials and Methods

### Ethics Statement

PsychoGenics is an AAALAC accredited facility (Unit Number– 001213) and work is performed under PHS OLAW Assurance #A4471-01. This study was carried out in strict accordance with the recommendations in the Guide for the Care and Use of Laboratory Animals of the National Institutes of Health. The protocol was approved by the Committee on the Ethics of Animal Experiments of PsychoGenics. All efforts were made to minimize suffering and maximize animal welfare.

### Subjects

#### 16p11.2 line

Breeders: A cohort of 40 WT female B6129SF1/J (catalog #101043) and 20 *df*/+ male B6129S-Del(7Slx1b-Sept1)4Aam/J mice (catalog #013128) were provided by The Jackson Laboratory at 5.1–6.1 weeks of age. B6129SF1/J mice are F1 offspring of C57BL/6J females and 129SvImJ males. B6129S-Del(7Slx1b-Sept1)4Aam/J mice are generated from frozen sperm of *df*/+ mice mated to C57BL/6J mice for a minimum of two generations and used to fertilize B6129SF1/J oocytes. This line is maintained at Jackson Laboratory, where resultant *df*/+ mice are bred with B6129SF1/J mice.

#### *Cntnap2* line

Breeders: A cohort of 40 female and 20 male *Cntnap2* -/- (B6.129(Cg)-*Cntnap2* ^*tm1Pele*^/J) mice (catalog #017482), backcrossed to C57BL/6J for more than 10 generations, was provided by The Jackson Laboratory at 4.1–9.4 weeks of age. Breeders for the last experimental cohort 3 of the *Cntnap2* line were unrelated heterozygous offspring of the original breeders.

#### PsychoGenics breeding scheme

Mice were set in trios (2 females:1 male) and left together for three days. Breeding was done three times to generate experimental animals. Breeders were 8.0–9.0, 15.9–17.0, and 26.0–27.0 weeks of age when bred for the 16p11.2 line cohorts 1–3, and 8.0–11.6, 15.0–19.6, and 6.1–7.1 weeks of age when bred for the *Cntnap2* line cohorts 1–3. See S1 Results and S4 Table for breeding efficacy, gender, and genotype ratios. All testing was done in male mice unless otherwise noted. Mutants and their wild type controls were littermates. As specified by the original developer of the test [78], two-month old 129SVE male mice (Taconic Farms) were used as stimulus mice in the three-chamber test. Age-matched male (~P45) unfamiliar littermates were used as stimulus mice for the homogenous social interaction design.

### General Procedures

Mice were housed in OptiMice cages (Animal Care Systems, Inc.) on a 12/12hr light/dark cycle where 20–23°C room temperature and a relative humidity of 50% was maintained. Chow and water was provided ad libitum for the duration of the study and mice were checked twice daily for general health, food, and water. Husbandry included enrichment, namely, shredded paper (Enviro-Dri; W.F. Fisher & Son Inc., NJ; Product 08ENV3000) and a nylabone (Bio-Serv, NJ; Product K3200). Breeders also received an amber-colored polycarbonate igloo for extra enrichment (Bio-Serv, NJ; Product K3328). On P0, pups were tattooed using non-toxic ink applied under the skin of their toe and a tail snip sample was taken for genotyping (see S1 and S2 Methods; S1 and S2 Figs, S1 and S2 Tables for genotyping protocol). Once the genotype results were available (around P2), the litter size was culled down to N = 8 pups, removing mainly females via decapitation. Thus, litter size range was (3–8) after culling. Animals were weaned in 2:2 mixed-genotype, same sex groups of four with shredded paper, one nylabone, and one polycarbonate amber-colored tunnel (3 7/8” long x 2” inside diameter) (Bio-Serv, NJ; Product K3323) per cage. Testing occurred between 10:00 and 17:30 in separate experimental rooms. Tests were conducted blind to genotype. Tables 3 and 4 show the series of tests completed longitudinally by the same animals per cohort. A small third cohort was bred to increase the number of neonates tested as the first round of testing produced slightly less than the 16 males required. As the first round of breeding did not produce enough mice to reach the goal of 16 mice, we included 6 females in the *Cntnap2* -/- and associated WT group and later added males form the third cohort to complete 16 males for the neonatal test. As inclusion of females did not change the statistical results we present the results of all pups (16 males and 6 females per group). Females were not tested in any of the adult assays. Euthanasia is required at first signs of illness, severe dehydration and/or emaciation defined as a loss of greater than 20% body weight with failure to regain weight while on a free feeding regimen, lack of righting reflex, catalepsy, morbidity, increased repetitive convulsions, respiratory distress, or hemorrhage. Although no mice were euthanized for any of these reasons, mice were sacrificed at the end of the study using methods consistent with recommendations of the 2013 American Veterinary Medical Association (AVMA) Guidelines on Euthanasia. Carbon dioxide gas was used and euthanasia was verified by observation of breathing and color of the animal, and by palpation of the heart in addition to loss of reflexes. Required further verification of death was accomplished via cervical dislocation.

**Table 3:** Time-Course of Tests for Cohort 1.

Observations in Order	P30	P60	P80	P90
**1 Body weight**	√	√	√	√
**2 3-chamber test**		√		
**3 T-maze**		√		
**4 SmartCube**				√

**Table 4:** Time-Course of Tests for Cohort 2.

Observations in Order	P30	P45-52	P60	P90
**1 Body weight**	√		√	√
**2 NeuroCube**	√		√	
**3 Reciprocal interaction (same genotype pairs)**		√		
**4 Reciprocal interaction (WT mouse stimulus)**		√		
**5 Marble burying**			√	
**6 Urine open field**			√	
**7 Startle-PPI**			√	

### Neonatal Assessment

Neonatal phenotyping assessments including body temperature and isolation tests were conducted at P4, P7 and P15 (see also S3 Methods and S3 Table) [48, 79]. The following were measured during the isolation tests: frequencies of square crossing, pivot, stretch attend, grooming, rearing, sniffing, jumping, rolling, and ultrasonic vocalization, geotaxis, and righting reflex. Immediately after the animals’ second temperature was recorded, animals were tested in the geotaxis and righting reflex tests. Body weight was measured last.

The cage with the mother and the litter was taken to the testing suite with a room temperature of about 24°C. The mothers were taken out of the cages and placed in new cages with water and food available and left in an anteroom until her pups had all been tested. Pups remained in the home cage with familiar bedding and nest material and were placed inside an incubator at ~34°C for a 30 min adaptation period in the interior testing room. The time that the pups were placed in the incubator was noted and incubator temperature was monitored and controlled at all times.

At the start of testing, the time was noted along with the temperature of the room and incubator. One pup at a time was removed from the incubator and the axial body temperature was immediately taken. The pup was then placed in a clean Plexiglas chamber atop a laminated grid-paper for a 2 minute observation. One person scored square crossing, pivot, stretch attempt, rolling, sniffing, rearing, jumping, and grooming frequency while another person counted ultrasonic vocalization (USV) calls using a Peterson bat detector (model D100). The isolation USV response was also measured by means of an ultrasound microphone placed about 6 cm above the pup. USV were recorded with Med Associates microphones and software (ANL-940-1 microphone and amplifier; RECORDER software; Med Associates Inc., St Albans, VT). As the sensitivity of this system proved low for possible accurate future frequency analysis, a second group of pups was used to record USVs through microphones with Avisoft software (Avisoft CM16/CMPA microphone with UltraSoundGate 416Hb; RECORDER USGH software). At the end of 2 minutes, the axial body temperature (T2) was taken and tests for geotaxis and righting reflex were performed.

#### Geotaxis

Tested the ability of the animal to orient itself when placed face down on an inclined platform (approximately 35° inclination). This test measured motor coordination and the vestibular system. Animals had a maximum of 60 seconds to complete the test. A second attempt was given if the animal fell. When the animal was able to complete the task in less than 60 seconds, that time was noted. Otherwise, the level of completion at the end of 60 seconds was indicated.

#### Righting reflex (RR)

The ability of one animal to right after being placed on its back. A righting reflex assessment scored the time (in seconds) it took the animal to right itself. The animal was given 3 consecutive righting reflex trials and a maximum of 30 s to complete each trial.

#### Milk score

Milk was scored as present or not; in few cases at P7 it was difficult to decide as the skin becomes relatively less transparent and very fine fur starts to grow. Once testing for each animal was completed, the pup was clearly marked with a Sharpie to indicate that the animal had been tested and was returned to the home cage inside of the incubator. After the litter had been tested, the cage with the pups was taken back to the anteroom and the mother was placed in the cage with the pups and the familiar bedding. The family was then brought to the colony room and left without further disturbance.

#### Eye opening

Eyes were scored at P13 as closed, half open and open (scores of 0, 0.5 and 1, respectively). A combined score for both eyes was obtained by summing the two scores and used for analysis.

### Three-Chamber Test

Procedures and apparatus were based on those described in the literature [78]. The apparatus was a clear Plexiglas chamber (L: 83.82cm x W: 40.64cm x H: 22.86cm), divided in three equally sized compartments separated by 2 sliding doors (L: 5cm x H: 8cm). The choice test had four 10-minute phases: (1) Habituation 1 –the test mouse was placed in the middle chamber and allowed to explore the middle chamber with the doorways into the two side chambers closed; (2) Habituation 2 –the doorways into the two side chambers were opened and the mouse was allowed to explore all three chambers of the apparatus; (3) Sociability–the test mouse was allowed to explore the three chambers with an unfamiliar stimulus mouse enclosed in a wire cage (D: 10cm, H: 10.5cm) in one side chamber and an empty wire cage (novel object) in the other; (4) Preference for Social Novelty—the test mouse was allowed to explore the three chambers with an unfamiliar stimulus mouse in the wire cage that was previously empty and the first stimulus mouse (now familiar) remained on the other. The location of the first stimulus mouse alternated between the left and the right sides of the social test box across subjects. Measures were taken of the amount of time spent in each chamber by the Limelight tracking software (ActiMetrics). In addition, a human observer scored the time spent sniffing each wire cage. The nose was required to be within 1 cm of the wire cage for this behavior to be scored. Stimulus mice used in the experiment were 2 month-old 129SVE male mice (Taconic Farms) that had been habituated to the wire cages over three 10-min sessions.

### T-Maze

Mice were brought into the experimental room 30 min prior to testing. Each mouse underwent 8 trials per day and each trial lasted a maximum of 60 s. The water was maintained at 25±1°C, and the platform was located 0.5–1 cm below the surface of the water. The water was opaque, and the maze was constructed of black Plexiglas. During procedural acquisition, mice were trained to go to one side of the maze, utilizing an egocentric strategy (that is, to always turn left or turn right- counterbalanced across subjects- after reaching the end of the stem), to locate the platform and escape the water. Criterion for acquisition was 6 correct choices for two consecutive days. During reversal, for 6 consecutive days mice were required to go to the opposite side in order to reach the platform. Incorrect arm choices were resulted in closure of the incorrect arm door for 10 s. After 10 s, the door was lifted and mice were then allowed to reach the platform in the opposite arm for trial completion. Mice remained on the platform for 10 s. Measures included proportion of mice that reached the platform during acquisition and reversal, days required to reach criterion for mice that succeeded, and percent correct during reversal.

### SmartCube

To enable phenotyping of mouse models of human disorders and testing of compounds for behavioral effects relevant to psychiatric disease, PsychoGenics developed SmartCube, an automated system in which mouse behavior is captured by digital video using novel, proprietary hardware that presents multiple challenges in a test session and is analyzed with computer algorithms. Digital videos of the subjects are processed with computer vision algorithms to extract more than 1400 dependent measures including frequency and duration of behavioral states such as grooming, rearing, etc., and many other features obtained during the test session. Using machine learning techniques chosen to best separate pharmacological effects of reference compounds, the behavioral signatures of the mutant mice are then assessed quantitatively [38, 40–42].

Mice were taken in their home cage to the SmartCube suite of experimental rooms where they remained until they were placed in the apparatus. A standard SmartCube protocol runs for a single session (~45 min). After the session mice were group-housed again and brought back to the colony room. Any abnormal behavior was noted.

### NeuroCube

The NeuroCube (NRC) system is a platform that employs computer vision to detect changes in gait geometry and gait dynamics in rodents [38]. Mice were allowed to acclimate in the experimental room for 1 h prior to test. Following acclimation to the test room mice were placed in the NeuroCube system and allowed to walk in the apparatus for a 5-min session. Digital videos of the subjects were processed through computer segmentation algorithms. The resulting fitted parameters were then analyzed to extract clips of locomotor behavior. Those clips were further analyzed to extract information about splay, gait, base, paw position, paw image intensity, limb coordination and body movement, among other features. The data obtained in this way were used to define a phenotypic signature. The most dominant features that define the disease phenotype (symptom descriptors) were identified and ranked. Complex bioinformatics’ algorithms were employed to calculate the discrimination probability between the mutant and corresponding WT mice.

NeuroCube features include features describing the geometric aspects of gait such as stride length, step length and base width. Dynamic features include stride duration, stand duration and swing duration. In addition to speed, so-called “paw features” include the area, intensity, perimeter, and minimal and maximal diameter of the paw image. Other measures include body motion (amount of change and variability of its dimension). NeuroCube also measures the coordination between limbs and the position of the paws (relative to body center and the angles defined by every possible three-paw positions).

### Reciprocal Interaction

#### Same genotype pairs

Subject animals were isolated for 2 days before testing. The day before testing, subject animals were individually habituated to the testing apparatus for 10 min and same-genotype stimulus animals (that were not of the same litter, were unfamiliar, and age-matched) were separately habituated to the apparatus in pairs. The day of testing, subject animals were placed in the testing apparatus for 5 min before a genotype- age- and weight-matched stimulus animal was placed into the chamber. Behavior and USVs were recorded for the pair for a total of 10 minutes.

#### WT stimulus

Subject animals were isolated for 2 days before testing. The day of testing, subject animals were placed in the testing apparatus for 5 min before an age- and weight-matched C57 wild type stimulus mouse (that were from the same colony, not of the same litter, were unfamiliar, and age-matched) was placed into the chamber. Behavior and ultrasonic vocalizations were recorded for the pair for a total of 10 min. As results with the wild type stimulus were identical to those with the same genotype stimulus, we present here the latter experiment but include the former in S7 and S8 Figs

#### Behavioral measures

Ethovision XT (Noldus Information Technology, Wageningen, Netherlands) was used to measure distance, proximity, and interaction between animals. We defined close proximity as the center of the bodies being between 1 and 5 cm apart, and interactions when the distance from the nose to the other mouse body (nose, center, and tail) was less than 1.5 cm. Interactions of the mice that were active (one mouse sniffing any part of the other mouse body), passive (recipient of the other subject’s investigation) and reciprocal (both mice actively sniffing each other) were scored manually for the first 5 minutes of the test period.

### Marble Burying

Upon individual placement in a cage containing glass marbles, mice have been shown to bury the marbles [80]. On test day, mice were brought to the experimental room for at least 1 hr acclimation to the experimental room conditions prior to testing. The mice were placed individually in clean mouse cages containing approximately 6 cm of hard wood bedding and twenty black marbles placed in spaced rows of 5 for a 30 min test session. Distance traveled during the test was captured by cameras overhead and quantified using VideoTracker Software (Viewpoint Life Sciences Software, France). At the end of the test mice were removed from the cages and the number of unburied marbles was counted. A marble was considered buried if it was covered at least two thirds with bedding.

### Urine-Open Field

Procedures were based on those described in the literature [81]. One week before the test, males were exposed to same-strain females for 5 minutes in a novel cage with fresh bedding. The day before test a handful of soiled male bedding was placed in the female cages to induce estrus. Estrus was determined by visual inspection of the vaginal area. The open field was conducted in a dimly lit room. The adult male mice were placed in a clean open field, lined with paper (Strathmore Drawing Paper Premium, recycled, microperforated, 400 series; Strathmore Artist Papers, Neenah, WI, USA) and containing some of its own home cage bedding in a corner of the arena. Open field activity was recorded for 60 min. At the end of the habituation period, the mouse was placed back in a clean polycarbonate cage with fresh bedding. The home cage bedding and any feces deposited by the mouse were removed from the open field. Urinary scent marks deposited on the paper during habituation were visualized under ultraviolet (UV) light and outlined with pencil for subsequent quantification. Fifteen microliters of fresh female urine, pooled from 4–6 estrous females, was then pipetted onto the center of the Strathmore paper, and the mouse was placed back into the open field for 5 min. Open field activity and ultrasonic vocalizations were recorded. The marked sheets of Strathmore paper were treated with Ninhydrin spray (LC-NIN-16; TritechForensics, Inc., Southport, NC, USA) then left to dry for ~12 hours, which allowed the visualization of the urine traces as purple spots.

Once dry, images were scanned and opened in ImageJ (U. S. National Institutes of Health, Bethesda, Maryland, USA). Freehand selections of the circled areas (pre-exposure marking) were removed and copied into a new JPEG image. The pre-exposure and post-exposure images were processed in 8-bit, with background subtracted, and converted to binary. Particles were analyzed at 1000-Infinity (pixels) and 0.00–1.00 (circularity), counted, and their area measured.

### Startle-PPI

The acoustic startle measures an unconditioned reflex response to external auditory stimulation. The prepulse-inhibition of startle (PPI), measures the degree of inhibition of the startle response following the presentation of a weak auditory stimulus or prepulse [64]. Mice were placed in the PPI chambers (Med Associates Inc., St Albans, VT) for a 5 min-session of white noise (70 dB) habituation. After the acclimation period, the test session automatically started with a habituation block of 6 presentations of the startle stimulus alone, followed by 10 PPI blocks of 6 different types of trials. Trial types were: null (no stimuli), startle (120 dB), startle plus prepulse (4, 8, and 12 dB over background noise i.e. 74, 78, or 82 dB) and prepulse alone (82 dB). Trial types were presented at random within each block. Each trial started with a 50 ms null period during which baseline movements were recorded. There was a subsequent 20 ms period during which prepulse stimuli were presented and responses to the prepulse were measured. After further 100 ms the startle stimuli were presented for 40 ms and responses recorded for 100 ms from startle onset. Responses were sampled every millisecond. The inter-trial interval was variable with an average of 15 s (range from 10 to 20 s). In startle alone trials the basic auditory startle was measured and in prepulse plus startle trials the amount of inhibition of the normal startle was determined and expressed as a percentage of the basic startle response (from startle-alone trials), excluding the startle response of the first habituation block.

### Bioinformatics for SmartCube and NeuroCube

The most dominant of the features collected that define the phenotype (symptom descriptors) were identified and ranked using complex proprietary bioinformatics algorithms and an overall discrimination index was calculated for all features combined or for different subsets of features. Graphical representations of the datasets corresponding to the groups compared were derived and a p-value was calculated to assess the statistical significance of the discrimination ratios. Top representative features were graphically presented to aid interpretation of differences (see S4 Methods) [38].

#### Feature analysis: De-correlation and ranking

The outcome of a SmartCube run is a set of ~1400 features (behavioral parameters) that can be used for various analyses. Many of these features are correlated (e.g. rearing counts and supported rearing counts). Therefore, we form statistically independent combinations of the original features (further referred to as de-correlated features) that discriminate between the two groups more effectively. Each de-correlated feature extracts information from the whole cluster of the original features, so the new feature space has lower dimensionality. Next, we apply a proprietary feature ranking algorithm to score each feature discrimination power (ability to separate the two groups, e.g. control and disease). Ranking is an important part of our analyses because it weighs each feature change by its relevance: if there is a significant change in some irrelevant feature measured for a particular phenotype, the low rank of this feature will automatically reduce the effect of such change in our analyses, so we do not have to resort to the conventional "feature selection" approach and discard information buried in the less informative features (S3 Fig). The ranking algorithm can be applied to either original or the new features to gain insight about the key control-disease differences.

#### Feature analysis: Quantitative assessment of disease phenotype

In the new decorrelated feature space, the overlap between the “clouds” (Gaussian distributions approximating the groups of mice in the ranked de-correlated features space) serves as a quantitative measure of separability ("distinguishability") between the two groups (S4 Fig). For visualization purposes, we plot each cloud with its semi-axes equal to the one standard deviation along the corresponding dimensions. Note, however, that while the overlap between any two Gaussian distributions is always non-zero, it may not necessarily be seen at the "1-sigma level". As in over-determined systems the discrimination index sometimes can be artificially high, we calculate its statistical significance by estimating the probability that the result is due to chance.

#### Top features identification

Working back from the discrimination analysis we can identify the features that contribute the most to the separation between two groups. Although statistical significance for differences between groups for the individual top features can be calculated, the alpha value for such statistical exercise cannot be set to the standard *p* = .05, as dozens of features are measured and combed for differences. Instead of over interpreting such top features we present them, in order to understand the mutant signatures, but refrain from performing misleading standard statistical tests.

### Data Handling

For all tests unless noted otherwise, statistical analyses consisted of one- or two-way ANOVAs (StatView for Windows Version 5.0.1, SAS Institute Inc., Cary, NC) with Genotype as a between-subjects factor and, when appropriate, Session, or Stimulus type as within-subject factor. Significant interactions between within and between-subject factors were followed by simple main effects (SPSS, IBM). The level of significance was set at *p* < .05. No outliers were removed. For repeated measures ANOVAs, the data of a subject was removed when data was missing for such subject at a time point. For body weight, the data of cohorts 1 and 2 were combined as no significant main effects of Cohort or interaction with Genotype was found. Categorical data was analyzed with Mann Whitney and frequency data was analyzed with Chi Square or Fisher Exact as noted.

#### Penetrance z-score analysis

To rescale all (*F*) behavioral endpoint measures (features, herein) for comparison across experimental tests we calculated the z-score, z_f,i_ for each feature (*f*) and for each mouse (*i*), for each model separately. To calculate z_f,i_ we first took the mean and standard deviation across the all mice corresponding to one model (*N* WT and *M* mutant mice). We could then characterize the ranking of each mouse *i* within the model group taking the average score *z*_*i*_ across features *f*. To avoid cancelling positive scores with negative scores, we first took the absolute value of *z*_*f*,*i*_. Thus, zi=1F∑f=1F|zf,i|. We expected that, if mutant mice are extreme every time they are tested, their average *z*_*i*_ will be higher than those of WT mice and therefore the mutant (MT) group mean, zMT=1M∑i=1Mzi will also be higher than the WT group mean, *zWT=1N∑i=M+1M+Nzi*. If mice obtained identical *z*_*f*,*i*_ for every feature, their average *z*_*i*_ will be identical to each *z*_*f*,*i*_, ∀*i*, and its ranking for each feature will also be its overall rank.

Assuming that the variability for each feature across mutant mice is σ_*f*,*MT*_ and for WT mice is = σ_*f*,*WT*_ (we simplify for clarity and assume that all features present similar variance), if the performance of a mouse in the F features are perfectly correlated (reflecting a trait), the variability of its overall z_i_ would be *σ*_*MT*_ = σ_*f*,*MT*_. If features are not correlated (perhaps reflecting a state), and a mouse performs unpredictably for each feature, then z_i_ will be closer to the mean across mice, *z*_*MT*_ or *z*_*WT*_, and the standard deviation, *σ*_*MT*_ or *σ*_*WT*,_ will be smaller. Therefore, if in one group of mice (such as the *df*/+ mice) the penetrance of the mutation is incomplete and some individuals are very extreme in all experimental tests, and some are not, then both the group average *z*_*MT*_ and its variability *σ*_*MT*_ will be higher.

## Supporting Information

S1 Fig PCR gel showing a molecular size marker (M) and samples for the WT (1) and df/+ mouse (2). (TIFF)

S2 Fig PCR gel showing a molecular size marker (M) and samples for the WT (1) and-/- mouse (2). (TIFF)

S3 Fig Difference in feature values and feature ranks (green symbols). Relative normalized difference (%) between feature values in two different sets is calculated and plotted in the order corresponding to feature ranks together with their ranks varying from 0 to 100%.
(TIFF)

S4 Fig Visualization of binary discrimination in the ranked de-correlated feature space. The two highest ranked de-correlated features are chosen to form the 2D coordinate plane for visualization purposes. Each dot represents a mouse. Mice from the control group are shown as blue dots and mice from the disease group are plotted in red. The other convenient (from a scale perspective) but equivalent measure derived from the cloud overlap is discrimination probability = 1—overlap which measures how reliably a classifier can be trained to discriminate between groups A and B above the chance level zero corresponding to 100% overlap and no ability to distinguish the two groups above the chance level whereas 100% meaning the error free discrimination.
(TIFF)

S5 Fig Neonatal tests. During the neonatal, at the three ages studied, mutants and their corresponding controls did not show phenotypic differences for A) the number of grid-paper squares crossed and B) baseline temperature. Data shown are means ± SEM.
(TIFF)

S6 Fig The three-chamber test of social behavior showed some differences between mutants and the corresponding WT mice. A: During the baseline habituation session 16p11.2 mice of both genotypes showed a slight preference for the right chamber. Cntnap2 -/- mice showed less exploration of the side chambers than the corresponding WT mice. B-C: A mouse preference sociability (B) and recognition (C) index built combining the time in the side chambers showed no genotypic differences for either model. D: During the sociability test, whereas for the 16p11.2 df/+ model there were no effects of genotype or chamber type, Cntnap2 -/- mice and their control littermates crossed over to the mouse chamber more than to the object chamber. E: Similarly, during the recognition test, whereas for the 16p11.2 df/+ model there were no effects of genotype or chamber type, Cntnap2 -/- mice and their control littermates crossed over to the novel mouse chamber more than to the familiar mouse chamber. Data shown are means ± SEM. Asterisks refer to differences between genotypes. Numerals refer to differences between chamber types (Chamber side main effect: ^#^p < .05; ^##^p < .01; Genotype main effect: *p < .05).
(TIFF)

S7 Fig In the reciprocal interaction test there were few significant differences. A & D: Mutant and WT mice travelled similar distances; B & E: The distance between paired mice was shorter for the 16p11.df/+ mice, although the difference reached significance only with the same genotype stimulus mouse; C & F: The time in close proximity (less than 5 cm) was not significantly different between WT and mutant mice. Data shown are means ± SEM (*p < .05). GT = genotype; WT = wild type.
(TIFF)

S8 Fig In the reciprocal social interaction test there were minor differences between WT and mutant mice. A & D: The percent of the session time that mice spent either actively, passively or reciprocally interacting with each other was not different between mutant and WT mice of either model, with either the same genotype stimulus or the wild type stimulus; B & E: The number of times per minute that mice engaged in active, passive or reciprocal interaction was similar for the WT and mutant mice, in both designs; C & F: Mice spent more time with their nose close to the back of the paired mouse, than to the front or side. With the same genotype stimulus mouse, the 16p11.2 df/+ mice spent more time close to the partner’s back than the WT mice. Data shown are means ± SEM (*p < .05). GT = genotype; WT = wild type.
(TIFF)

S9 Fig In the procedural T-maze mutant and WT mice learned the task at a similar rate as shown by the percent correct choice during reversal. Data shown are means ± SE.
(TIFF)

S10 Fig Averaged z-scores across tests for each mouse. No differences were found comparing mutant and WT mice. Data shown are means ± SE.
(TIFF)

S1 Methods Genotyping of the 16p11.2 by PCR assay. (PDF)

S2 Methods Genotyping of the Cntnap2 Model by PCR assay. (PDF)

S3 Methods Time-Course of Neonatal Tests. (PDF)

S4 Methods Bioinformatics for SmartCube and NeuroCube. (PDF)

S1 Results Breeding Efficacy, Gender, and Genotype Ratio. (PDF)

S1 Table PCR conditions for the phenotyping of the 16p11.2 deletion model. (PDF)

S2 Table PCR conditions for the phenotyping of the Cntnap2 knockout model. (PDF)

S3 Table Postnatal day (P) of neonatal testing for both models. (PDF)

S4 Table Number of litters, pups, gender and genotype ratios, and survival up to weaning day. (PDF)

S5 Table SmartCube results for the 16p11.2 deletion model. (PDF)

S6 Table SmartCube results for the Cntnap2 knockout model. (PDF)

S7 Table NeuroCube results for the 16p11.2 deletion model. (PDF)

S8 Table NeuroCube results for the Cntnap2 knockout model. (PDF)

S9 Table General health data for the 16p11.2 deletion model. (PDF)

S10 Table General health data for the Cntnap2 model. (PDF)

S11 Table Activity and ultrasonic vocalizations in the 16p11.2 deletion model. (PDF)

S12 Table Activity and ultrasonic vocalizations in the Cntnap2 knockout model. (PDF)

S13 Table Motor coordination and reflexes in the 16p11.2 deletion model. (PDF)

S14 Table Motor coordination and reflexes in the Cntnap2 knockout model. (PDF)

S15 Table 3-chamber test for the 16p11.2 deletion model. (PDF)

S16 Table 3-chamber test for the Cntnap2 knockout model. (PDF)

S17 Table Reciprocal social interaction test for the 16p11.2 deletion model, same genotype stimulus. (PDF)

S18 Table Reciprocal social interaction test for the 16p11.2 deletion model, WT stimulus. (PDF)

S19 Table Reciprocal social interaction test for the Cntnap2 knockout model, same genotype stimulus. (PDF)

S20 Table Reciprocal social interaction test for the Cntnap2 knockout model, WT stimulus. (PDF)

S21 Table Urine exposure open field test for the 16p11.2 deletion model. (PDF)

S22 Table Urine exposure open field test for the Cntnap2 knockout model. (PDF)

S23 Table T-maze test for the 16p11.2 deletion model. (PDF)

S24 Table T-maze test for the Cntnap2 knockout model. (PDF)

S25 Table Startle and prepulse inhibition of startle for the 16p11.2 deletion model. (PDF)

S26 Table Startle and prepulse inhibition of startle for the Cntnap2 knockout model. (PDF)

S27 Table Marble burying test for the 16p11.2 deletion model. (PDF)

S28 Table Marble burying test for the Cntnap2 knockout model. (PDF)

S29 Table Neonatal testing raw data. (PDF)

S30 Table 3-chamber test raw data. (PDF)

S31 Table Reciprocal social interaction test raw data. (PDF)

S32 Table Urine exposure open field test raw data. (PDF)

S33 Table T-maze test raw data. (PDF)

S34 Table Startle and prepulse inhibition of startle raw data. (PDF)

S35 Table Marble burying test raw data. (PDF)

## References

[1] Menalled L, El-Khodor BF, Patry M, Suarez-Farinas M, Orenstein SJ, Zahasky B, et al Systematic behavioral evaluation of Huntington's disease transgenic and knock-in mouse models. Neurobiol Dis. 2009;35(3):319–36. Epub 2009/05/26. S0969-9961(09)00107-7 [pii] 10.1016/j.nbd.2009.05.007 19464370 PMC2728344

[2] Sanders SJ, Murtha MT, Gupta AR, Murdoch JD, Raubeson MJ, Willsey AJ, et al De novo mutations revealed by whole-exome sequencing are strongly associated with autism. Nature. 2012;485(7397):237–41. Epub 2012/04/13. nature10945 [pii] 10.1038/nature10945 22495306 PMC3667984

[3] O'Roak BJ, Vives L, Girirajan S, Karakoc E, Krumm N, Coe BP, et al Sporadic autism exomes reveal a highly interconnected protein network of de novo mutations. Nature. 2012;485(7397):246–50. Epub 2012/04/13. nature10989 [pii] 10.1038/nature10989 22495309 PMC3350576

[4] Iossifov I, Ronemus M, Levy D, Wang Z, Hakker I, Rosenbaum J, et al De novo gene disruptions in children on the autistic spectrum. Neuron. 2012;74(2):285–99. Epub 2012/05/01. S0896-6273(12)00340-6 [pii] 10.1016/j.neuron.2012.04.009 22542183 PMC3619976

[5] Consortium TD-BFX. Fmr1 knockout mice: a model to study fragile X mental retardation. Cell. 1994;78(1):23–33. Epub 1994/07/15. 0092-8674(94)90569-X [pii]. . 8033209

[6] Shi L, Fatemi SH, Sidwell RW, Patterson PH. Maternal influenza infection causes marked behavioral and pharmacological changes in the offspring. The Journal of neuroscience: the official journal of the Society for Neuroscience. 2003;23(1):297–302. . 12514227 10.1523/JNEUROSCI.23-01-00297.2003 PMC6742135

[7] Meyer U. Prenatal poly(i:C) exposure and other developmental immune activation models in rodent systems. Biological psychiatry. 2014;75(4):307–15. 10.1016/j.biopsych.2013.07.011 . 23938317

[8] Ingram JL, Peckham SM, Tisdale B, Rodier PM. Prenatal exposure of rats to valproic acid reproduces the cerebellar anomalies associated with autism. Neurotoxicol Teratol. 2000;22(3):319–24. Epub 2000/06/07. S0892-0362(99)00083-5 [pii]. . 10840175 10.1016/s0892-0362(99)00083-5

[9] Bolivar VJ, Walters SR, Phoenix JL. Assessing autism-like behavior in mice: variations in social interactions among inbred strains. Behavioural brain research. 2007;176(1):21–6. 10.1016/j.bbr.2006.09.007 17097158 PMC1831820

[10] Moy SS, Nadler JJ, Young NB, Perez A, Holloway LP, Barbaro RP, et al Mouse behavioral tasks relevant to autism: phenotypes of 10 inbred strains. Behavioural brain research. 2007;176(1):4–20. Epub 2006/09/15. S0166-4328(06)00443-8 [pii] 10.1016/j.bbr.2006.07.030 16971002 PMC1857288

[11] Betancur C, Sakurai T, Buxbaum JD. The emerging role of synaptic cell-adhesion pathways in the pathogenesis of autism spectrum disorders. Trends in neurosciences. 2009;32(7):402–12. 10.1016/j.tins.2009.04.003 . 19541375 PMC10354373

[12] Spooren W, Lindemann L, Ghosh A, Santarelli L. Synapse dysfunction in autism: a molecular medicine approach to drug discovery in neurodevelopmental disorders. Trends in pharmacological sciences. 2012;33(12):669–84. 10.1016/j.tips.2012.09.004 . 23084458

[13] Rubenstein JL, Merzenich MM. Model of autism: increased ratio of excitation/inhibition in key neural systems. Genes, brain, and behavior. 2003;2(5):255–67. . 14606691 10.1034/j.1601-183x.2003.00037.x PMC6748642

[14] Pietropaolo S, Guilleminot A, Martin B, D'Amato FR, Crusio WE. Genetic-background modulation of core and variable autistic-like symptoms in Fmr1 knock-out mice. PLoS One. 2011;6(2):e17073 Epub 2011/03/03. 10.1371/journal.pone.0017073 21364941 PMC3043074

[15] Deogracias R, Yazdani M, Dekkers MP, Guy J, Ionescu MC, Vogt KE, et al Fingolimod, a sphingosine-1 phosphate receptor modulator, increases BDNF levels and improves symptoms of a mouse model of Rett syndrome. Proc Natl Acad Sci U S A. 2012;109(35):14230–5. Epub 2012/08/15. 1206093109 [pii] 10.1073/pnas.1206093109 22891354 PMC3435172

[16] Kumar RA, KaraMohamed S, Sudi J, Conrad DF, Brune C, Badner JA, et al Recurrent 16p11.2 microdeletions in autism. Hum Mol Genet. 2008;17(4):628–38. Epub 2007/12/25. ddm376 [pii] 10.1093/hmg/ddm376 . 18156158

[17] Marshall CR, Noor A, Vincent JB, Lionel AC, Feuk L, Skaug J, et al Structural variation of chromosomes in autism spectrum disorder. Am J Hum Genet. 2008;82(2):477–88. Epub 2008/02/07. S0002-9297(07)00035-3 [pii] 10.1016/j.ajhg.2007.12.009 18252227 PMC2426913

[18] Walsh KM, Bracken MB. Copy number variation in the dosage-sensitive 16p11.2 interval accounts for only a small proportion of autism incidence: a systematic review and meta-analysis. Genet Med. 2011;13(5):377–84. Epub 2011/02/04. 10.1097/GIM.0b013e3182076c0c . 21289514

[19] Weiss LA, Shen Y, Korn JM, Arking DE, Miller DT, Fossdal R, et al Association between microdeletion and microduplication at 16p11.2 and autism. N Engl J Med. 2008;358(7):667–75. Epub 2008/01/11. NEJMoa075974 [pii] 10.1056/NEJMoa075974 . 18184952

[20] Zweier C, de Jong EK, Zweier M, Orrico A, Ousager LB, Collins AL, et al CNTNAP2 and NRXN1 are mutated in autosomal-recessive Pitt-Hopkins-like mental retardation and determine the level of a common synaptic protein in Drosophila. Am J Hum Genet. 2009;85(5):655–66. Epub 2009/11/10. S0002-9297(09)00459-5 [pii] 10.1016/j.ajhg.2009.10.004 19896112 PMC2775834

[21] Strauss KA, Puffenberger EG, Huentelman MJ, Gottlieb S, Dobrin SE, Parod JM, et al Recessive symptomatic focal epilepsy and mutant contactin-associated protein-like 2. N Engl J Med. 2006;354(13):1370–7. Epub 2006/03/31. 354/13/1370 [pii] 10.1056/NEJMoa052773 . 16571880

[22] Horev G, Ellegood J, Lerch JP, Son YE, Muthuswamy L, Vogel H, et al Dosage-dependent phenotypes in models of 16p11.2 lesions found in autism. Proc Natl Acad Sci U S A. 2011;108(41):17076–81. Epub 2011/10/05. 1114042108 [pii] 10.1073/pnas.1114042108 21969575 PMC3193230

[23] Portmann T, Yang M, Mao R, Panagiotakos G, Ellegood J, Dolen G, et al Behavioral abnormalities and circuit defects in the Basal Ganglia of a mouse model of 16p11.2 deletion syndrome. Cell Rep. 2014;7(4):1077–92. Epub 2014/05/06. S2211-1247(14)00214-9 [pii] 10.1016/j.celrep.2014.03.036 . 24794428 PMC4251471

[24] Penagarikano O, Abrahams BS, Herman EI, Winden KD, Gdalyahu A, Dong H, et al Absence of CNTNAP2 leads to epilepsy, neuronal migration abnormalities, and core autism-related deficits. Cell. 2011;147(1):235–46. Epub 2011/10/04. S0092-8674(11)01010-5 [pii] 10.1016/j.cell.2011.08.040 . 21962519 PMC3390029

[25] Bachmann-Gagescu R, Mefford HC, Cowan C, Glew GM, Hing AV, Wallace S, et al Recurrent 200-kb deletions of 16p11.2 that include the SH2B1 gene are associated with developmental delay and obesity. Genet Med. 2010;12(10):641–7. Epub 2010/09/03. 10.1097/GIM.0b013e3181ef4286 . 20808231

[26] Barge-Schaapveld DQ, Maas SM, Polstra A, Knegt LC, Hennekam RC. The atypical 16p11.2 deletion: a not so atypical microdeletion syndrome? Am J Med Genet A. 2011;155A(5):1066–72. Epub 2011/04/06. 10.1002/ajmg.a.33991 . 21465664

[27] Bijlsma EK, Gijsbers AC, Schuurs-Hoeijmakers JH, van Haeringen A, Fransen van de Putte DE, Anderlid BM, et al Extending the phenotype of recurrent rearrangements of 16p11.2: deletions in mentally retarded patients without autism and in normal individuals. Eur J Med Genet. 2009;52(2–3):77–87. Epub 2009/03/25. S1769-7212(09)00021-4 [pii] 10.1016/j.ejmg.2009.03.006 . 19306953

[28] Miller DT, Nasir R, Sobeih MM, Shen Y, Wu BL, Hanson E. 16p11.2 Microdeletion. 1993 Epub 2010/03/20. NBK11167 [bookaccession]. .20301775

[29] Shinawi M, Liu P, Kang SH, Shen J, Belmont JW, Scott DA, et al Recurrent reciprocal 16p11.2 rearrangements associated with global developmental delay, behavioural problems, dysmorphism, epilepsy, and abnormal head size. J Med Genet. 2010;47(5):332–41. Epub 2009/11/17. jmg.2009.073015 [pii] 10.1136/jmg.2009.073015 19914906 PMC3158566

[30] Zufferey F, Sherr EH, Beckmann ND, Hanson E, Maillard AM, Hippolyte L, et al A 600 kb deletion syndrome at 16p11.2 leads to energy imbalance and neuropsychiatric disorders. J Med Genet. 2012;49(10):660–8. Epub 2012/10/12. jmedgenet-2012-101203 [pii] 10.1136/jmedgenet-2012-101203 23054248 PMC3494011

[31] Girirajan S, Eichler EE. Phenotypic variability and genetic susceptibility to genomic disorders. Hum Mol Genet. 2010;19(R2):R176–87. Epub 2010/09/03. ddq366 [pii] 10.1093/hmg/ddq366 20807775 PMC2953748

[32] Sebat J, Lakshmi B, Malhotra D, Troge J, Lese-Martin C, Walsh T, et al Strong association of de novo copy number mutations with autism. Science. 2007;316(5823):445–9. Epub 2007/03/17. 1138659 [pii] 10.1126/science.1138659 17363630 PMC2993504

[33] Poliak S, Salomon D, Elhanany H, Sabanay H, Kiernan B, Pevny L, et al Juxtaparanodal clustering of Shaker-like K+ channels in myelinated axons depends on Caspr2 and TAG-1. J Cell Biol. 2003;162(6):1149–60. Epub 2003/09/10. doi: 10.1083/jcb.200305018 jcb.200305018 [pii]. 12963709 10.1083/jcb.200305018 PMC2172860

[34] Peippo M, Ignatius J. Pitt-Hopkins Syndrome. Mol Syndromol. 2012;2(3–5):171–80. Epub 2012/06/07. 000335287 msy-0002-0171 [pii]. 22670138 10.1159/000335287 PMC3366706

[35] Menalled L, Brunner D. Animal models of Huntington's disease for translation to the clinic: best practices. Mov Disord. 2014;29(11):1375–90. Epub 2014/09/13. 10.1002/mds.26006 . 25216369

[36] Lord C, Rutter M, Goode S, Heemsbergen J, Jordan H, Mawhood L, et al Autism diagnostic observation schedule: a standardized observation of communicative and social behavior. J Autism Dev Disord. 1989;19(2):185–212. Epub 1989/06/01. . 2745388 10.1007/BF02211841

[37] Bailey KR, Rustay NR, Crawley JN. Behavioral phenotyping of transgenic and knockout mice: practical concerns and potential pitfalls. ILAR J. 2006;47(2):124–31. Epub 2006/03/21. . 16547369 10.1093/ilar.47.2.124

[38] Alexandrov V, Brunner D, Hanania T, Leahy E. Highthroughtput analysis of behavior for drug discovery. European Journal of Pharmacology. 2015. 10.1016/j.ejphar.2015.02.037 25744878

[39] Balci F, Oakeshott S, Shamy JL, El-Khodor BF, Filippov I, Mushlin R, et al High-Throughput Automated Phenotyping of Two Genetic Mouse Models of Huntington's Disease. PLoS Curr. 2013;5 Epub 2013/07/19. 10.1371/currents.hd.124aa0d16753f88215776fba102ceb29 23863947 PMC3710674

[40] Brunner D, Nestler E, Leahy E. In need of high-throughput behavioral systems. Drug discovery today. 2002;7(18 Suppl):S107–12. . 12546875 10.1016/s1359-6446(02)02423-6

[41] Houghten RA, Pinilla C, Giulianotti MA, Appel JR, Dooley CT, Nefzi A, et al Strategies for the use of mixture-based synthetic combinatorial libraries: scaffold ranking, direct testing in vivo, and enhanced deconvolution by computational methods. J Comb Chem. 2008;10(1):3–19. Epub 2007/12/11. 10.1021/cc7001205 . 18067268

[42] Roberds SL, Filippov I, Alexandrov V, Hanania T, Brunner D. Rapid, computer vision-enabled murine screening system identifies neuropharmacological potential of two new mechanisms. Front Neurosci. 2011;5:103 Epub 2011/09/20. 10.3389/fnins.2011.00103 21927596 PMC3169783

[43] Green D, Charman T, Pickles A, Chandler S, Loucas T, Simonoff E, et al Impairment in movement skills of children with autistic spectrum disorders. Developmental medicine and child neurology. 2009;51(4):311–6. Epub 2009/02/12. 10.1111/j.1469-8749.2008.03242.x . 19207298

[44] Ament K, Mejia A, Buhlman R, Erklin S, Caffo B, Mostofsky S, et al Evidence for Specificity of Motor Impairments in Catching and Balance in Children with Autism. J Autism Dev Disord. 2014 Epub 2014/09/19. 10.1007/s10803-014-2229-0 . 25231287 PMC4342267

[45] Nebel MB, Joel SE, Muschelli J, Barber AD, Caffo BS, Pekar JJ, et al Disruption of functional organization within the primary motor cortex in children with autism. Human brain mapping. 2014;35(2):567–80. Epub 2012/11/03. 10.1002/hbm.22188 23118015 PMC3864146

[46] LeBarton ES, Iverson JM. Fine motor skill predicts expressive language in infant siblings of children with autism. Developmental science. 2013;16(6):815–27. Epub 2013/10/15. 10.1111/desc.12069 24118709 PMC3808875

[47] Hilton CL, Zhang Y, Whilte MR, Klohr CL, Constantino J. Motor impairment in sibling pairs concordant and discordant for autism spectrum disorders. Autism: the international journal of research and practice. 2012;16(4):430–41. Epub 2011/10/21. 10.1177/1362361311423018 22013131 PMC4222044

[48] Brunner D, Buhot MC, Hen R, Hofer M. Anxiety, motor activation, and maternal-infant interactions in 5HT1B knockout mice. Behav Neurosci. 1999;113(3):587–601. . 10443785 10.1037//0735-7044.113.3.587

[49] Jailer JW. The maturation of the pituitary-adrenal axis in the newborn rat. Endocrinology. 1950;46(5):420–5. Epub 1950/05/01. 10.1210/endo-46-5-420 . 15414816

[50] Blumberg MS, Alberts JR. Ultrasonic vocalizations by rat pups in the cold: an acoustic by-product of laryngeal braking? Behav Neurosci. 1990;104(5):808–17. Epub 1990/10/01. . 2244987 10.1037//0735-7044.104.5.808

[51] Hofer MA, Shair HN. Independence of ultrasonic vocalization and thermogenic responses in infant rats. Behav Neurosci. 1991;105(1):41–8. Epub 1991/02/01. . 2025393 10.1037//0735-7044.105.1.41

[52] Carden SE, Hofer MA. Effect of a social companion on the ultrasonic vocalizations and contact responses of 3-day-old rat pups. Behav Neurosci. 1992;106(2):421–6. Epub 1992/04/01. . 1590959 10.1037//0735-7044.106.2.421

[53] Knutson B, Burgdorf J, Panksepp J. Ultrasonic vocalizations as indices of affective states in rats. Psychological bulletin. 2002;128(6):961–77. . 12405139 10.1037/0033-2909.128.6.961

[54] Trevarthen C, Daniel S. Disorganized rhythm and synchrony: early signs of autism and Rett syndrome. Brain Dev. 2005;27 Suppl 1:S25–S34. Epub 2005/09/27. S0387-7604(05)00128-2 [pii] 10.1016/j.braindev.2005.03.016 . 16182487

[55] Gemelli T, Berton O, Nelson ED, Perrotti LI, Jaenisch R, Monteggia LM. Postnatal loss of methyl-CpG binding protein 2 in the forebrain is sufficient to mediate behavioral aspects of Rett syndrome in mice. Biological psychiatry. 2006;59(5):468–76. Epub 2005/10/04. S0006-3223(05)00914-5 [pii] 10.1016/j.biopsych.2005.07.025 . 16199017

[56] Moy SS, Nadler JJ, Perez A, Barbaro RP, Johns JM, Magnuson TR, et al Sociability and preference for social novelty in five inbred strains: an approach to assess autistic-like behavior in mice. Genes, brain, and behavior. 2004;3(5):287–302. Epub 2004/09/04. 10.1111/j.1601-1848.2004.00076.x GBB076 [pii]. . 15344922

[57] Lehmann ML, Geddes CE, Lee JL, Herkenham M. Urine scent marking (USM): a novel test for depressive-like behavior and a predictor of stress resiliency in mice. PLoS One. 2013;8(7):e69822 Epub 2013/07/23. 10.1371/journal.pone.0069822 PONE-D-12-39888 [pii]. 23875001 PMC3713058

[58] Novotny M, Harvey S, Jemiolo B. Chemistry of male dominance in the house mouse, Mus domesticus. Experientia. 1990;46(1):109–13. Epub 1990/01/15. . 2298278 10.1007/BF01955433

[59] Roullet FI, Wohr M, Crawley JN. Female urine-induced male mice ultrasonic vocalizations, but not scent-marking, is modulated by social experience. Behavioural brain research. 2010;216(1):19–28. Epub 2010/06/15. S0166-4328(10)00432-8 [pii] 10.1016/j.bbr.2010.06.004 20540967 PMC3094925

[60] Tanimura Y, Yang MC, Lewis MH. Procedural learning and cognitive flexibility in a mouse model of restricted, repetitive behaviour. Behavioural brain research. 2008;189(2):250–6. Epub 2008/02/15. S0166-4328(08)00002-8 [pii] 10.1016/j.bbr.2008.01.001 . 18272239

[61] DeCoteau WE, Thorn C, Gibson DJ, Courtemanche R, Mitra P, Kubota Y, et al Learning-related coordination of striatal and hippocampal theta rhythms during acquisition of a procedural maze task. Proc Natl Acad Sci U S A. 2007;104(13):5644–9. Epub 2007/03/21. 0700818104 [pii] 10.1073/pnas.0700818104 17372196 PMC1838454

[62] Frankland PW, Wang Y, Rosner B, Shimizu T, Balleine BW, Dykens EM, et al Sensorimotor gating abnormalities in young males with fragile X syndrome and Fmr1-knockout mice. Mol Psychiatry. 2004;9(4):417–25. Epub 2004/02/26. doi: 10.1038/sj.mp.4001432 4001432 [pii]. . 14981523 10.1038/sj.mp.4001432

[63] McAlonan GM, Daly E, Kumari V, Critchley HD, van Amelsvoort T, Suckling J, et al Brain anatomy and sensorimotor gating in Asperger's syndrome. Brain. 2002;125(Pt 7):1594–606. Epub 2002/06/22. . 12077008 10.1093/brain/awf150

[64] Swerdlow NR, Braff DL, Geyer MA, Koob GF. Central dopamine hyperactivity in rats mimics abnormal acoustic startle response in schizophrenics. Biological psychiatry. 1986;21(1):23–33. Epub 1986/01/01. 0006-3223(86)90005-3 [pii]. . 3080033 10.1016/0006-3223(86)90005-3

[65] Gyertyan I. Analysis of the marble burying response: marbles serve to measure digging rather than evoke burying. Behav Pharmacol. 1995;6(1):24–31. Epub 1995/01/01. . 11224308

[66] Broekkamp CL, Rijk HW, Joly-Gelouin D, Lloyd KL. Major tranquillizers can be distinguished from minor tranquillizers on the basis of effects on marble burying and swim-induced grooming in mice. Eur J Pharmacol. 1986;126(3):223–9. Epub 1986/07/31. . 2875886 10.1016/0014-2999(86)90051-8

[67] Egashira N, Tanoue A, Matsuda T, Koushi E, Harada S, Takano Y, et al Impaired social interaction and reduced anxiety-related behavior in vasopressin V1a receptor knockout mice. Behavioural brain research. 2007;178(1):123–7. Epub 2007/01/18. S0166-4328(06)00702-9 [pii] 10.1016/j.bbr.2006.12.009 . 17227684

[68] Kondo M, Gray LJ, Pelka GJ, Christodoulou J, Tam PP, Hannan AJ. Environmental enrichment ameliorates a motor coordination deficit in a mouse model of Rett syndrome—Mecp2 gene dosage effects and BDNF expression. Eur J Neurosci. 2008;27(12):3342–50. Epub 2008/06/19. EJN6305 [pii] 10.1111/j.1460-9568.2008.06305.x . 18557922

[69] Schneider T, Turczak J, Przewlocki R. Environmental enrichment reverses behavioral alterations in rats prenatally exposed to valproic acid: issues for a therapeutic approach in autism. Neuropsychopharmacology. 2006;31(1):36–46. Epub 2005/05/28. 1300767 [pii] 10.1038/sj.npp.1300767 . 15920505

[70] Wolfer DP, Litvin O, Morf S, Nitsch RM, Lipp HP, Wurbel H. Laboratory animal welfare: cage enrichment and mouse behaviour. Nature. 2004;432(7019):821–2. Epub 2004/12/17. 432821a [pii] 10.1038/432821a . 15602544

[71] Hammerschmidt K, Radyushkin K, Ehrenreich H, Fischer J. Female mice respond to male ultrasonic 'songs' with approach behaviour. Biology letters. 2009;5(5):589–92. 10.1098/rsbl.2009.0317 19515648 PMC2781958

[72] Weller A, Leguisamo AC, Towns L, Ramboz S, Bagiella E, Hofer M, et al Maternal effects in infant and adult phenotypes of 5HT1A and 5HT1B receptor knockout mice. Dev Psychobiol. 2003;42(2):194–205. Epub 2003/01/30. 10.1002/dev.10079 . 12555283

[73] Peñagarikano O, Abrahams BS, Herman EI, Winden KD, Gdalyahu A, Dong H, et al Absence of CNTNAP2 leads to epilepsy, neuronal migration abnormalities, and core autism-related deficits. Cell. 2011;147(1):235–46. Epub 2011/10/04. S0092-8674(11)01010-5 [pii] 10.1016/j.cell.2011.08.040 21962519 PMC3390029

[74] Peñagarikano O, Lazaro MT, Lu XH, Gordon A, Dong H, Lam HA, et al Exogenous and evoked oxytocin restores social behavior in the Cntnap2 mouse model of autism. Sci Transl Med. 2015;7(271):271ra8 Epub 2015/01/23. 7/271/271ra8 [pii] 10.1126/scitranslmed.3010257 . 25609168 PMC4498455

[75] Yang M, Mahrt EJ, Lewis F, Foley G, Portmann T, Dolmetsch RE, et al 16p11.2 Deletion Syndrome Mice Display Sensory and Ultrasonic Vocalization Deficits During Social Interactions. Autism Res. 2015 Epub 2015/02/11. 10.1002/aur.1465 . 25663600 PMC5321681

[76] Gerlai R. Gene-targeting studies of mammalian behavior: is it the mutation or the background genotype? Trends in neurosciences. 1996;19(5):177–81. Epub 1996/05/01. S0166-2236(96)20020-7 [pii]. . 8723200 10.1016/s0166-2236(96)20020-7

[77] Tian D, Stoppel LJ, Heynen AJ, Lindemann L, Jaeschke G, Mills AA, et al Contribution of mGluR5 to pathophysiology in a mouse model of human chromosome 16p11.2 microdeletion. Nat Neurosci. 2015;18(2):182–4. Epub 2015/01/13. nn.3911 [pii] 10.1038/nn.3911 25581360 PMC4323380

[78] Yang M, Silverman JL, Crawley JN. Automated three-chambered social approach task for mice. Current protocols in neuroscience / editorial board, Jacqueline N Crawley [et al]. 2011;Chapter 8:Unit 8 26 Epub 2011/07/07. 10.1002/0471142301.ns0826s56 . 21732314 PMC4904775

[79] El-Khodor BF, Dimmler MH, Amara DA, Hofer M, Hen R, Brunner D. Juvenile 5HT(1B) receptor knockout mice exhibit reduced pharmacological sensitivity to 5HT(1A) receptor activation. Int J Dev Neurosci. 2004;22(5–6):405–13. . 15380839 10.1016/j.ijdevneu.2004.06.001

[80] Njung'e K, Handley SL. Effects of 5-HT uptake inhibitors, agonists and antagonists on the burying of harmless objects by mice; a putative test for anxiolytic agents. Br J Pharmacol. 1991;104(1):105–12. Epub 1991/09/01. 1686200 10.1111/j.1476-5381.1991.tb12392.x PMC1908295

[81] Wohr M, Roullet FI, Crawley JN. Reduced scent marking and ultrasonic vocalizations in the BTBR T+tf/J mouse model of autism. Genes, brain, and behavior. 2010;10(1):35–43. Epub 2010/03/30. GBB582 [pii] 10.1111/j.1601-183X.2010.00582.x 20345893 PMC2903641

