# Supplementary figures and images for: Comprehensive Analysis of the 16p11.2 Deletion and Null Cntnap2 Mouse Models of Autism Spectrum Disorder
Source: PLoS One. 2015 Aug 14;10(8):e0134572. doi: 10.1371/journal.pone.0134572 (PMC4537259; doi:10.1371/journal.pone.0134572)

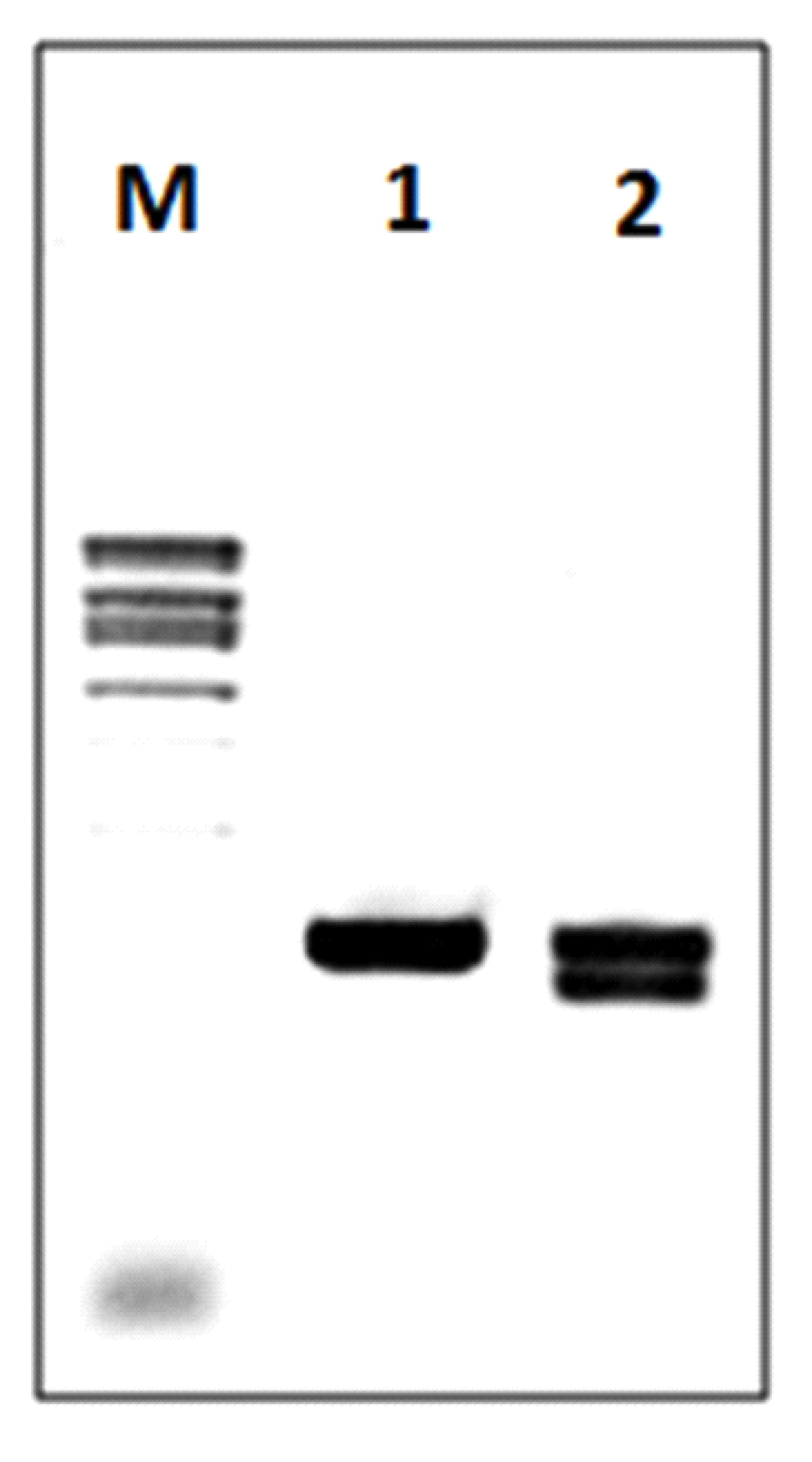

Supplement: S1 Fig — (TIFF) [file pone.0134572.s001.tiff]

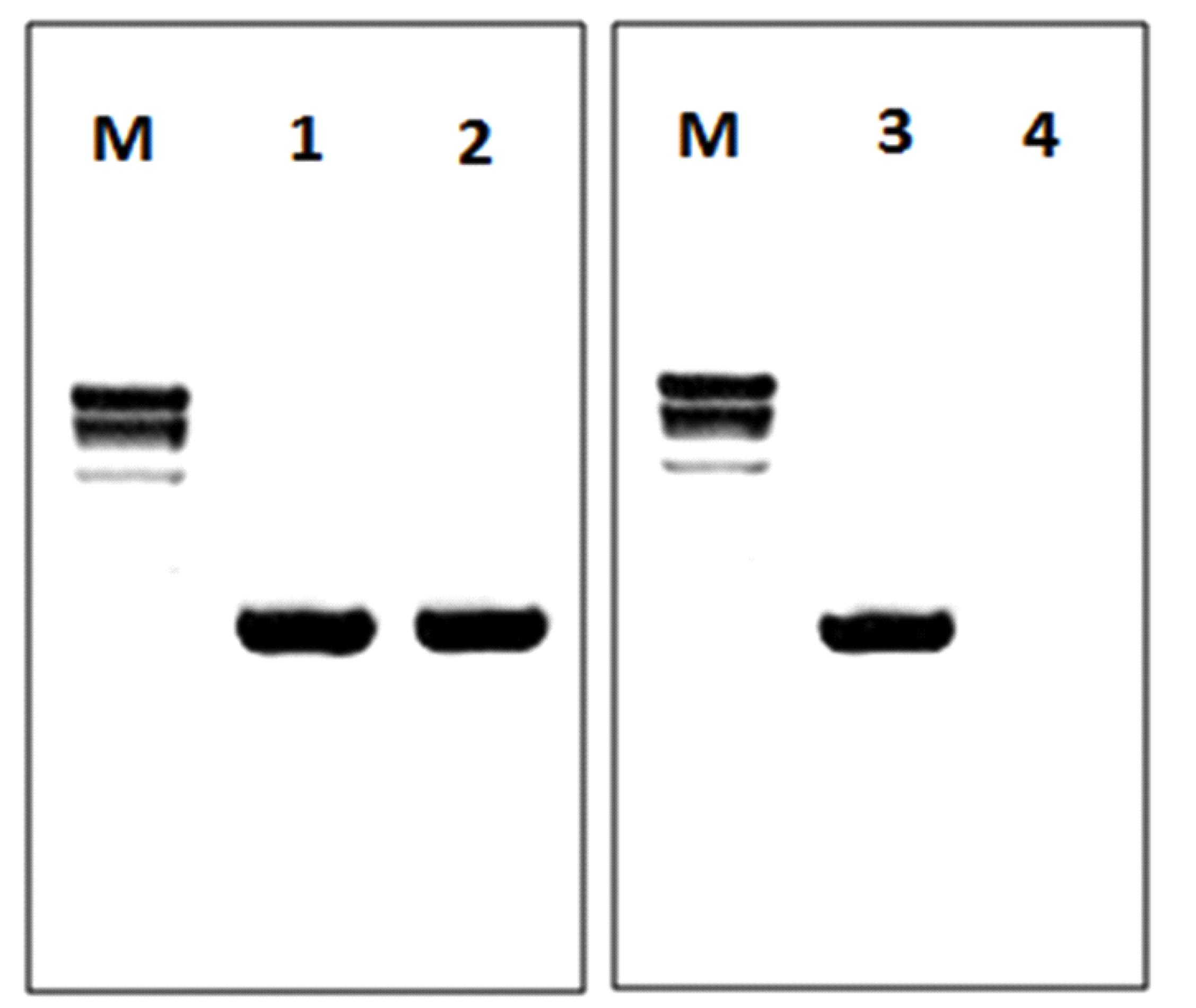

Supplement: S2 Fig — (TIFF) [file pone.0134572.s002.tiff]

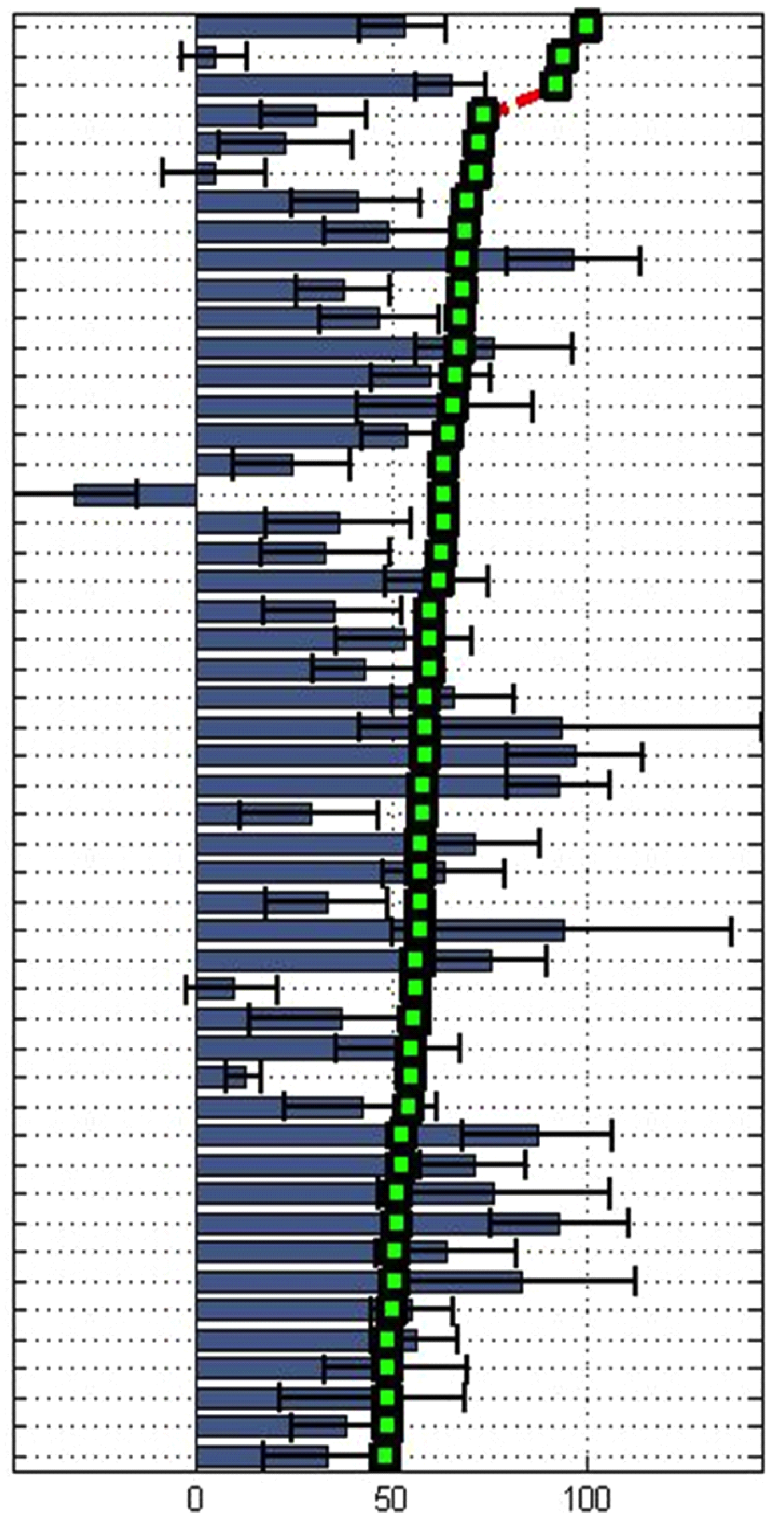

Supplement: S3 Fig — Relative normalized difference (%) between feature values in two different sets is calculated and plotted in the order corresponding to feature ranks together with their ranks varying from 0 to 100%. (TIFF) [file pone.0134572.s003.tiff]

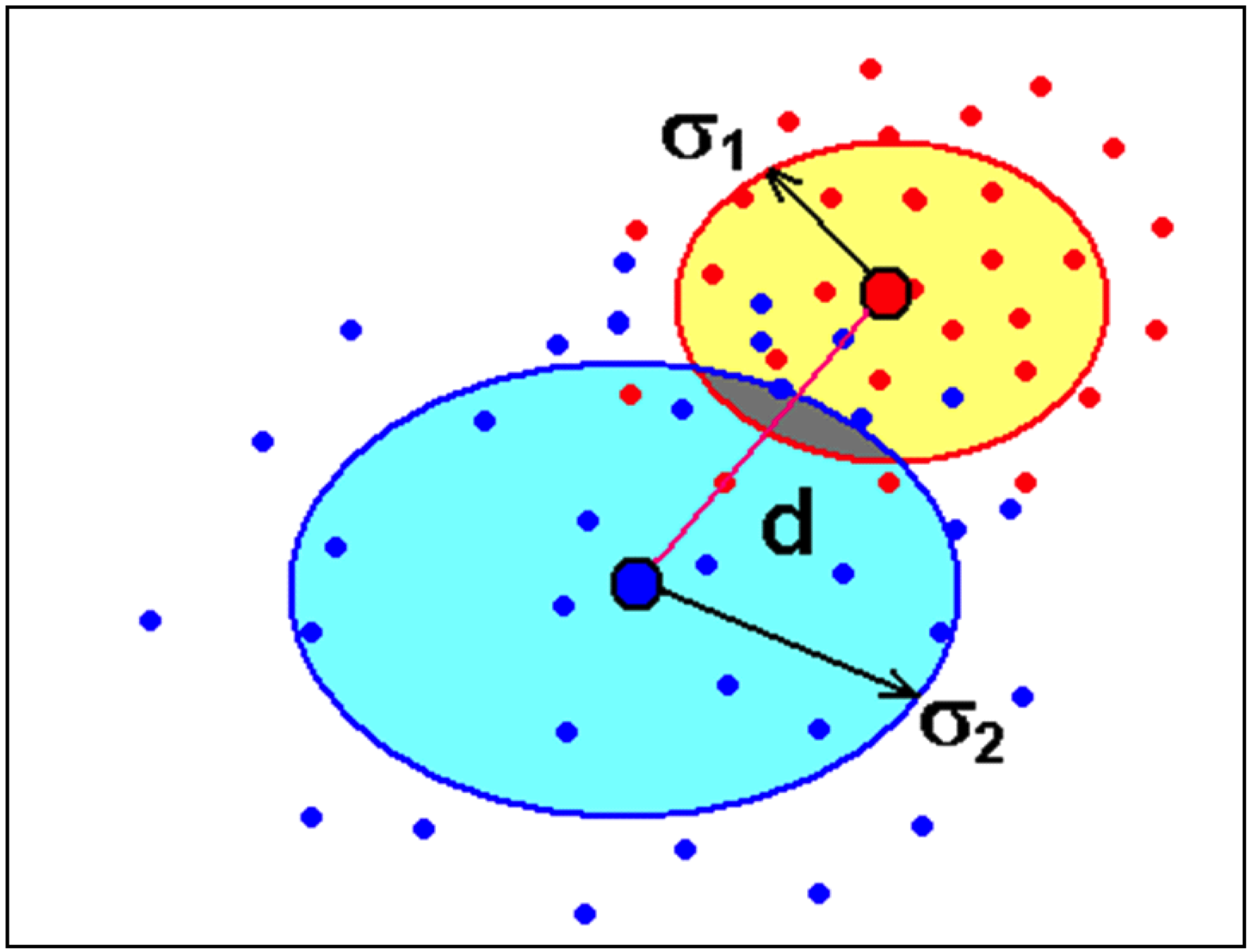

Supplement: S4 Fig — The two highest ranked de-correlated features are chosen to form the 2D coordinate plane for visualization purposes. Each dot represents a mouse. Mice from the control group are shown as blue dots and mice from the disease group are plotted in red. The other convenient (from a scale perspective) but equivalent measure derived from the cloud overlap is discrimination probability = 1—overlap which measures how reliably a classifier can be trained to discriminate between groups A and B above the chance level zero corresponding to 100% overlap and no ability to distinguish the two groups above the chance level whereas 100% meaning the error free discrimination. (TIFF) [file pone.0134572.s004.tiff]

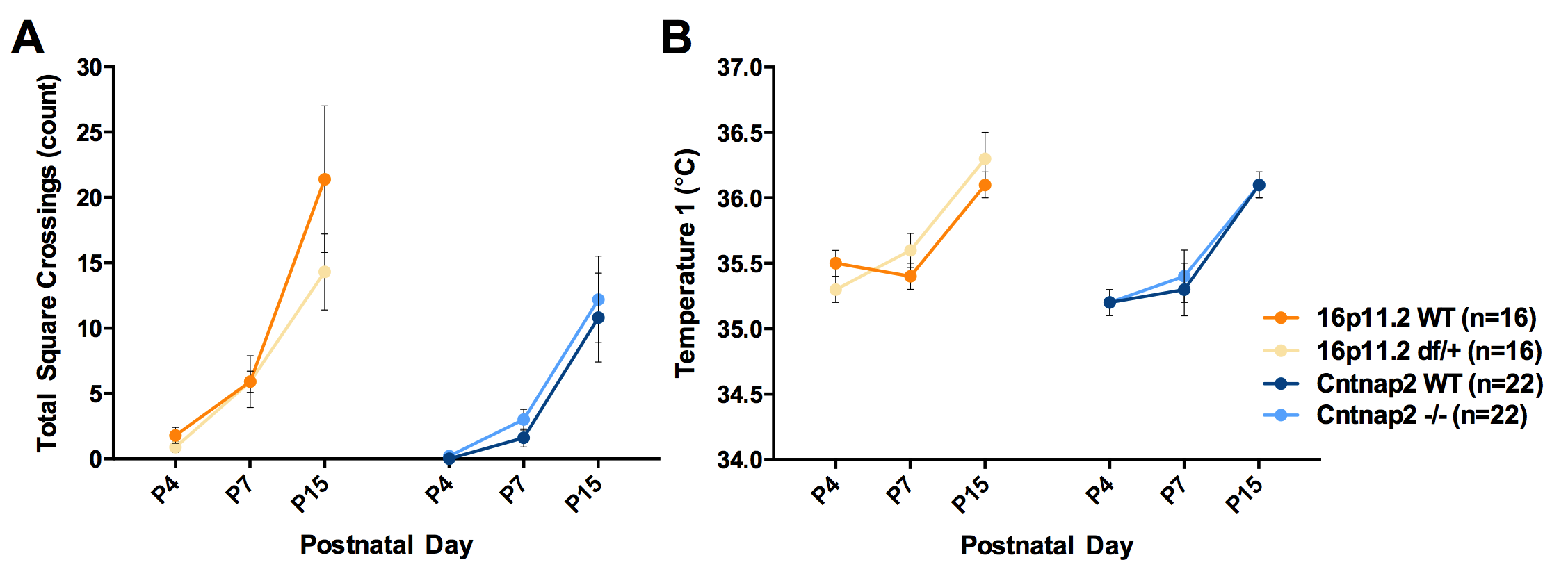

Supplement: S5 Fig — During the neonatal, at the three ages studied, mutants and their corresponding controls did not show phenotypic differences for A) the number of grid-paper squares crossed and B) baseline temperature. Data shown are means ± SEM. (TIFF) [file pone.0134572.s005.tiff]

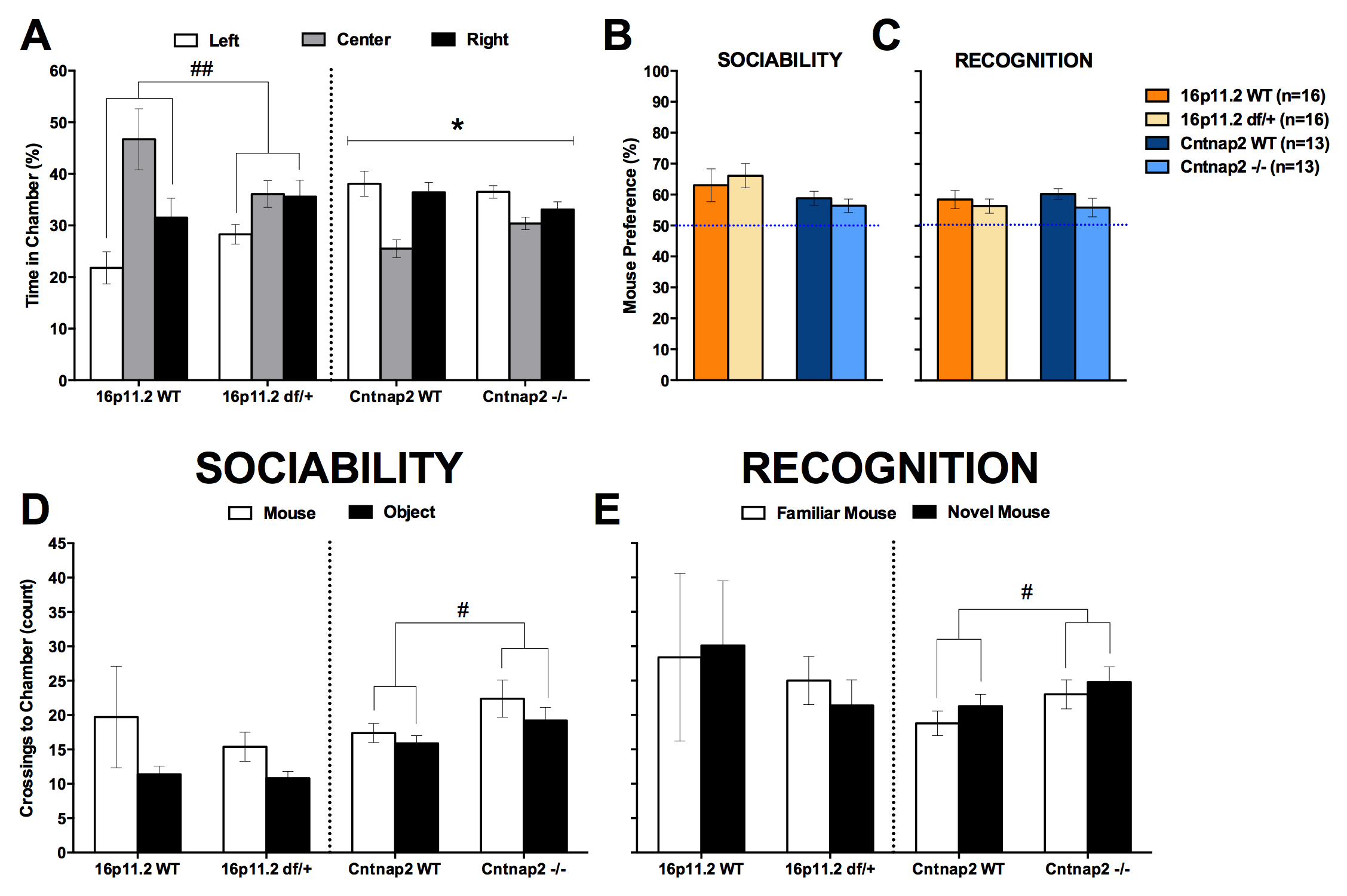

Supplement: S6 Fig — A: During the baseline habituation session 16p11.2 mice of both genotypes showed a slight preference for the right chamber. Cntnap2 -/- mice showed less exploration of the side chambers than the corresponding WT mice. B-C: A mouse preference sociability (B) and recognition (C) index built combining the time in the side chambers showed no genotypic differences for either model. D: During the sociability test, whereas for the 16p11.2 df/+ model there were no effects of genotype or chamber type, Cntnap2 -/- mice and their control littermates crossed over to the mouse chamber more than to the object chamber. E: Similarly, during the recognition test, whereas for the 16p11.2 df/+ model there were no effects of genotype or chamber type, Cntnap2 -/- mice and their control littermates crossed over to the novel mouse chamber more than to the familiar mouse chamber. Data shown are means ± SEM. Asterisks refer to differences between genotypes. Numerals refer to differences between chamber types (Chamber side main effect: #p < .05; ##p < .01; Genotype main effect: *p < .05). (TIFF) [file pone.0134572.s006.tiff]

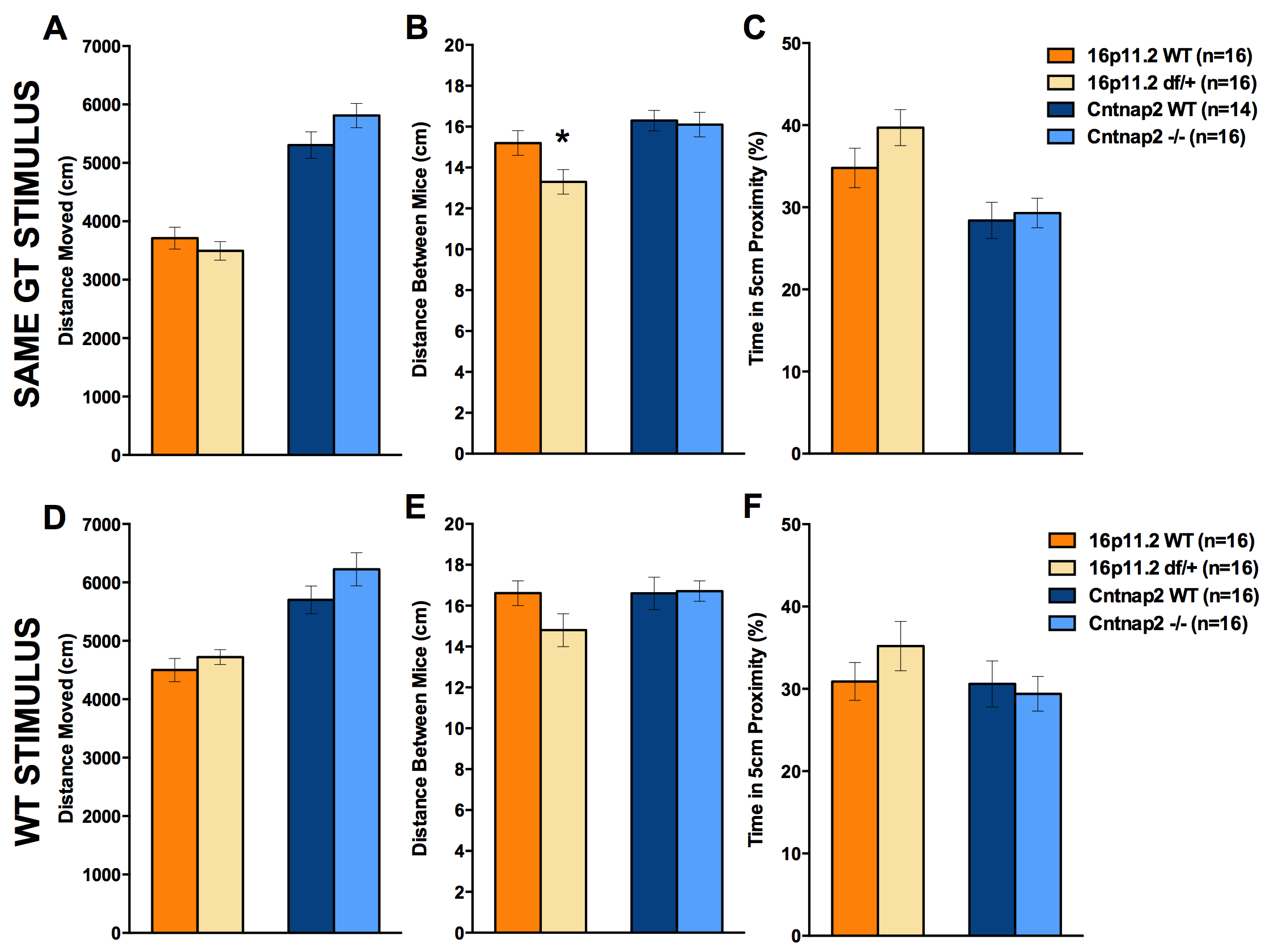

Supplement: S7 Fig — A & D: Mutant and WT mice travelled similar distances; B & E: The distance between paired mice was shorter for the 16p11.df/+ mice, although the difference reached significance only with the same genotype stimulus mouse; C & F: The time in close proximity (less than 5 cm) was not significantly different between WT and mutant mice. Data shown are means ± SEM (*p < .05). GT = genotype; WT = wild type. (TIFF) [file pone.0134572.s007.tiff]

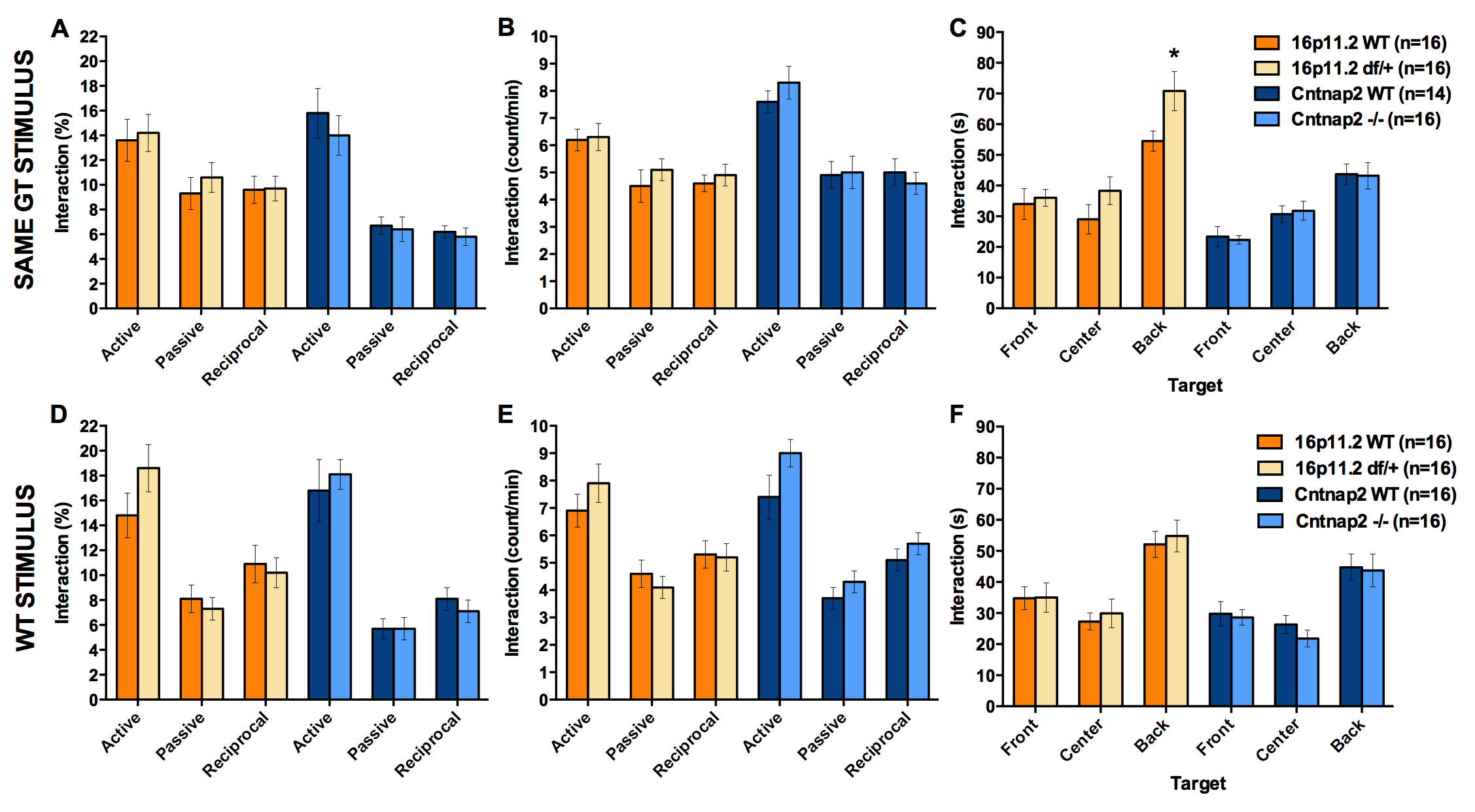

Supplement: S8 Fig — A & D: The percent of the session time that mice spent either actively, passively or reciprocally interacting with each other was not different between mutant and WT mice of either model, with either the same genotype stimulus or the wild type stimulus; B & E: The number of times per minute that mice engaged in active, passive or reciprocal interaction was similar for the WT and mutant mice, in both designs; C & F: Mice spent more time with their nose close to the back of the paired mouse, than to the front or side. With the same genotype stimulus mouse, the 16p11.2 df/+ mice spent more time close to the partner’s back than the WT mice. Data shown are means ± SEM (*p < .05). GT = genotype; WT = wild type. (TIFF) [file pone.0134572.s008.tiff]

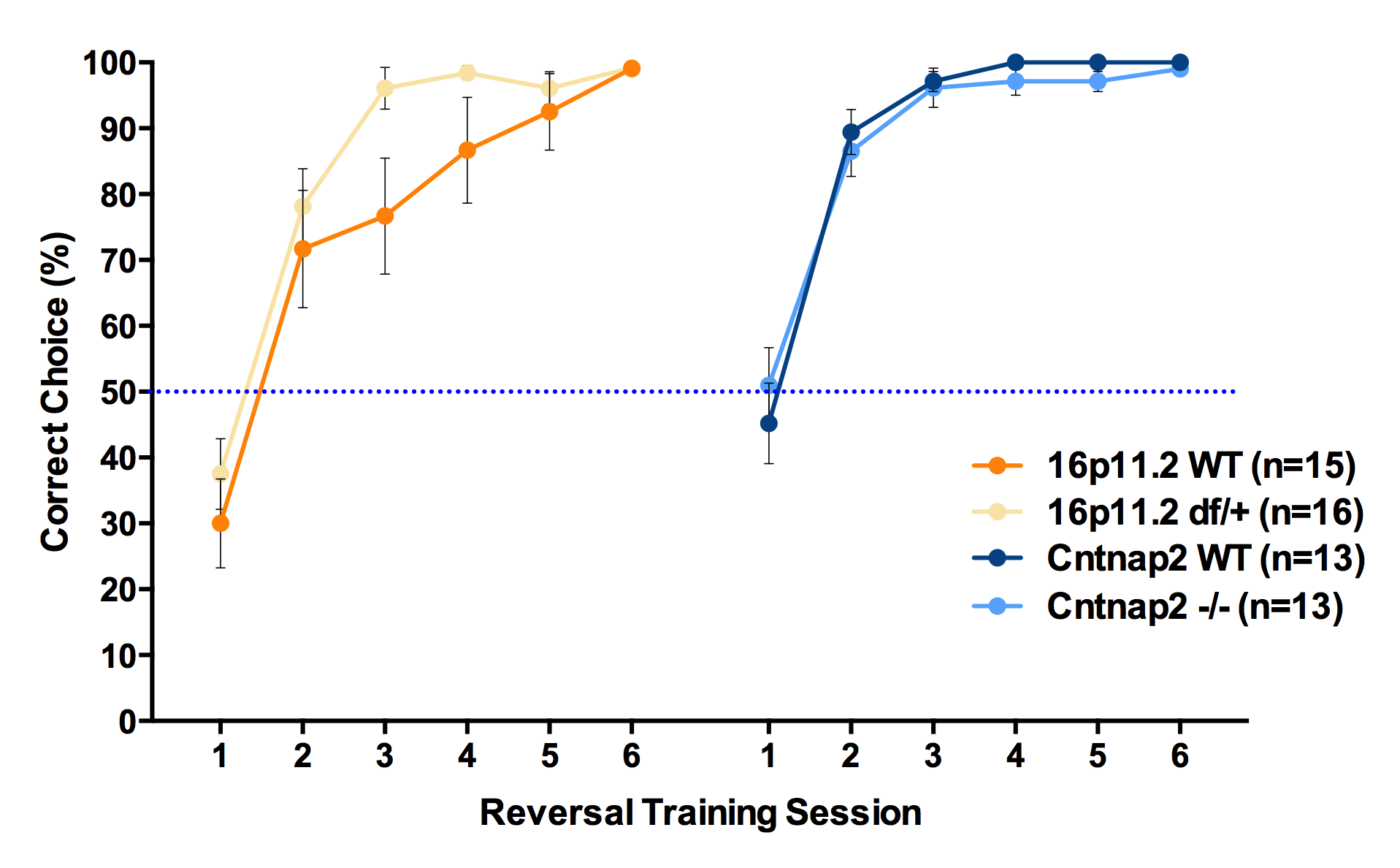

Supplement: S9 Fig — Data shown are means ± SE. (TIFF) [file pone.0134572.s009.tiff]

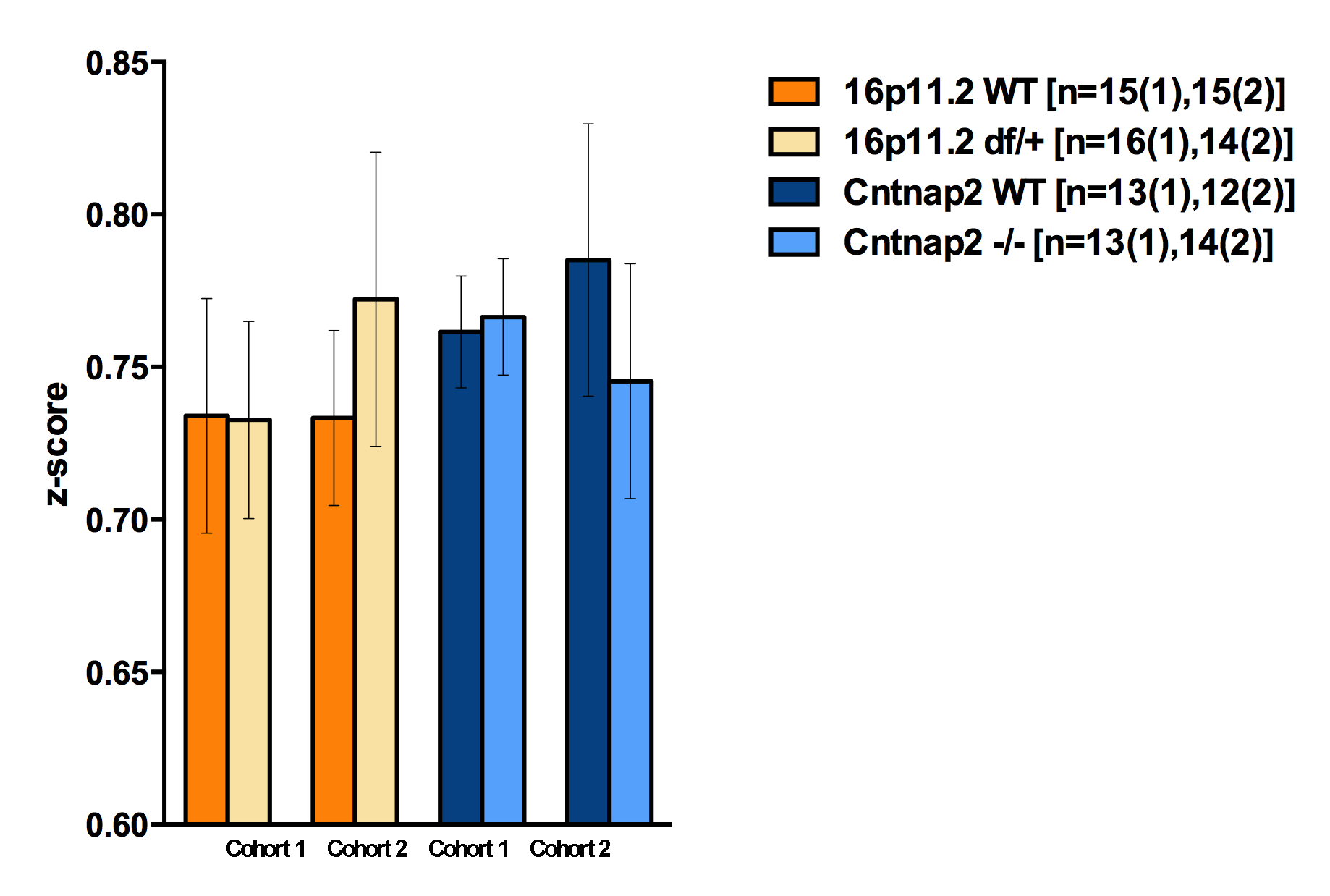

Supplement: S10 Fig — No differences were found comparing mutant and WT mice. Data shown are means ± SE. (TIFF) [file pone.0134572.s010.tiff]
