# Supplementary material for: Comprehensive Analysis of the 16p11.2 Deletion and Null Cntnap2 Mouse Models of Autism Spectrum Disorder
Source: PLoS One. 2015 Aug 14;10(8):e0134572. doi: 10.1371/journal.pone.0134572 (PMC4537259; doi:10.1371/journal.pone.0134572)
Supplement: S1 Methods — (PDF) [file pone.0134572.s011.pdf]

S1 Methods. Genotyping of the 16p11.2 by PCR assay.

Genotyping was done by Mouse Genotype (958 Sea Wind Court Carlsbad, CA 92011), based on protocols provided by the Jackson Lab (Figure_S1, Table_S1).

Sep1 PCR assay:

Sep1 -/- - ~250 bp		Sep1 WT - 306 bp
12634:	CAAGCACTGGCTATGCATGT	
12635:	AAGACAGAATGCTATGCAACCTT	
12636:	GCTATGAAGATGCCCACCAT	
12637:	GTGGTAACTCATGCGGTCCT	
