# Supplementary material for: Comprehensive Analysis of the 16p11.2 Deletion and Null Cntnap2 Mouse Models of Autism Spectrum Disorder
Source: PLoS One. 2015 Aug 14;10(8):e0134572. doi: 10.1371/journal.pone.0134572 (PMC4537259; doi:10.1371/journal.pone.0134572)
Supplement: S2 Methods — (PDF) [file pone.0134572.s012.pdf]

S2 Methods. Genotyping of the Cntnap2 Model by PCR assay.

Genotyping was done by Mouse Genotype (958 Sea Wind Court Carlsbad, CA 92011), based on protocols provided by the Jackson Lab (Figure_S2, Table_S2).

Cntnap2 WT - 351 bp	
13634 (Common):	CTGCCAGCCCAGAACTGG
13635 (WT R):	AGTTGATACCCGAGCGCC
Cntnap2 -/- - ~350 bp	
13634 (Common):	CTGCCAGCCCAGAACTGG
10791 (-/- R):	CGCTTCCTCGTGCTTTACGGTAT
