# Supplementary material for: Comprehensive Analysis of the 16p11.2 Deletion and Null Cntnap2 Mouse Models of Autism Spectrum Disorder
Source: PLoS One. 2015 Aug 14;10(8):e0134572. doi: 10.1371/journal.pone.0134572 (PMC4537259; doi:10.1371/journal.pone.0134572)
Supplement: S3 Methods — (PDF) [file pone.0134572.s013.pdf]

S3 Methods. Time-Course of Neonatal Tests.

Most pups were generated in the first breeding round. As the first breeding did not generate the required 16 pups for the *Cntnap2* model, 3 WT and 3 *Cntnap2*-/- mice from the second cohort were also used for neonatal assessment (Table_S3).
