# Supplementary material for: Comprehensive Analysis of the 16p11.2 Deletion and Null Cntnap2 Mouse Models of Autism Spectrum Disorder
Source: PLoS One. 2015 Aug 14;10(8):e0134572. doi: 10.1371/journal.pone.0134572 (PMC4537259; doi:10.1371/journal.pone.0134572)
Supplement: S4 Methods — (PDF) [file pone.0134572.s014.pdf]

S4 Methods. Bioinformatics for SmartCube and NeuroCube.

Feature analysis: de-correlation and ranking. The outcome of any of “Cube” analysis is a vector of hundreds of features (behavioral parameters) that can be used for various analyses (e.g. one run through SmartCube produces more than 1400 features whereas NeuroCube results in > 50 feature values). Many of these features are correlated (e.g. rearing counts and supported rearing counts). Therefore we form statistically independent combinations of the original features (further referred to as de-correlated features) that discriminate between the two groups more effectively. Each de-correlated feature extracts information from the whole cluster of the original features so the new feature space has lower dimensionality (Figure_S3).

Next we apply a proprietary feature ranking algorithm to score each feature’s discrimination power (ability to separate the two groups e.g. control and disease). Ranking is an important part of our analyses because it weighs each feature change by its relevance: if there is a significant change in some irrelevant feature measured for a particular phenotype the low rank of this feature will automatically reduce the effect of such change in our analyses so we don't have to resort to the conventional feature selection approach and discard information buried in the less informative features. The ranking algorithm can be applied to either the original or the new features to gain insight about the key control-disease differences (Figure_S3).

Feature analysis: quantitative assessment of Disease Phenotype. In the new feature space the overlap between the “clouds” (Gaussian distributions approximating the groups of mice in the ranked de-correlated features space) serves as a quantitative measure of separability (or distinguishability) between the two groups. For visualization purposes we plot each cloud with its semi-axes equal to the one standard deviation along the corresponding dimensions (Figure_ S4). Note however that while the overlap between any two Gaussian distributions is always non-zero it may not necessarily be seen at the 1-sigma level.

Significance. Discrimination significance (generalized p-value) is calculated in the following way. First, each labeled set of candidates is randomly split with 1:3 ratio, where larger groups from each set are used to calculate discrimination probability using methods previously described. This procedure is repeated multiple times with different random splitting for each iteration to build distribution of “true” discrimination probability p_{true} (step 1). Number of iterations is limited by a fraction of the total number of split combinations available. Next, all candidates from both groups are combined together without individual class labels (step 2). Similarly to the previous step, this set is split randomly multiple times. Larger group of candidates for each split is randomly divided into two “classes” and used to calculate discrimination probability. After many iterations, distribution of “random” discrimination probability p_{random} is built (step 2). Both distributions are normalized and their mutual weighted overlap is calculated. The resulting value is a generalized quantity of what is well known as p-value of statistical significance.
