# Supplementary material for: Comprehensive Analysis of the 16p11.2 Deletion and Null Cntnap2 Mouse Models of Autism Spectrum Disorder
Source: PLoS One. 2015 Aug 14;10(8):e0134572. doi: 10.1371/journal.pone.0134572 (PMC4537259; doi:10.1371/journal.pone.0134572)
Supplement: S1 Results — (PDF) [file pone.0134572.s015.pdf]

S1 Results. Breeding Efficacy, Gender, and Genotype Ratio. Table S4 shows the number of litters and breeding efficacy of the two models. Sex and genotype ratios were unbiased in both models. Animals in cohort 1 were weaned at 4 weeks of age due to small body size. The second cohort was weaned at 3 weeks of age in preparation for P30 testing. Breeding success and pup survival was higher in the mixed genetic background (Table_S4).
