# Supplementary material for: Comprehensive Analysis of the 16p11.2 Deletion and Null Cntnap2 Mouse Models of Autism Spectrum Disorder
Source: PLoS One. 2015 Aug 14;10(8):e0134572. doi: 10.1371/journal.pone.0134572 (PMC4537259; doi:10.1371/journal.pone.0134572)
Supplement: S2 Table — (PDF) [file pone.0134572.s017.pdf]

S2 Table. PCR conditions for the phenotyping of the Cntnap2 knockout model.

Step	***Temp***	***Time***	***Note***
1	94°C	3 min	
2	94°C	30 sec	
3	58°C	30 sec	
4	72°C	40 sec	Go to 2, 35 cycles
5	72°C	2 min	
6	4°C	Hold	
