# Supplementary material for: Comprehensive Analysis of the 16p11.2 Deletion and Null Cntnap2 Mouse Models of Autism Spectrum Disorder
Source: PLoS One. 2015 Aug 14;10(8):e0134572. doi: 10.1371/journal.pone.0134572 (PMC4537259; doi:10.1371/journal.pone.0134572)
Supplement: S3 Table — (PDF) [file pone.0134572.s018.pdf]

S3 Table. Postnatal day (P) of neonatal testing for both models.

Tests in Order	P1-P3	P4	P7	P13	P15
Survival	X	X	X	X	X
Body Weight		X	X		X
Eye Opening				X	
Milk Score Test		X	X		X
Body Temperature (T1)		X	X		X
Maternal Isolation		X	X		X
Body Temperature (T2)		X	X		X
Geotaxis		X	X		X
Righting Reflex		X	X		X
