# Supplementary material for: Comprehensive Analysis of the 16p11.2 Deletion and Null Cntnap2 Mouse Models of Autism Spectrum Disorder
Source: PLoS One. 2015 Aug 14;10(8):e0134572. doi: 10.1371/journal.pone.0134572 (PMC4537259; doi:10.1371/journal.pone.0134572)
Supplement: S4 Table — (PDF) [file pone.0134572.s019.pdf]

S4 Table. Number of litters, pups, gender and genotype ratios, and survival up to weaning day.

	**16p11.2**		**Cntnap2**	
**Cohort Number**	1	2	1	2
**Days Mated**	3	5-6	3	5-6
**Number of breeding pairs**	40	40	40	40
**Number of litters**	24	38	21	34
**Number of pups**	183	335	130	251
**Number of males (M)**	87	170	62	119
**Number of females (F)**	96	165	68	132
**Breeding yield (pups/litter)**	8.4		6.9	
**Ratio M:F**	0.98 (NS)		0.91 (NS)	
**Ratio WT:df/+ or WT:-/-**	1:0.8 ($p < 0.09$)		1:2.3:1.3 (NS)	
**Percent deaths**	2.1%		7.9%	

NS, non-significant.
