# Supplementary material for: Comprehensive Analysis of the 16p11.2 Deletion and Null Cntnap2 Mouse Models of Autism Spectrum Disorder
Source: PLoS One. 2015 Aug 14;10(8):e0134572. doi: 10.1371/journal.pone.0134572 (PMC4537259; doi:10.1371/journal.pone.0134572)
Supplement: S5 Table — (PDF) [file pone.0134572.s020.pdf]

S5 Table. SmartCube results for the 16p11.2 deletion model.

16p11.2					
	Measure		Genotype	Mean	*SE*
SmartCube	Repetitive Behavior	Grooming Time	WT	392.6	*54.6*
			HET	350.8	*49.7*
		Digging Time (s)	WT	0.3	*0.03*
			HET	0.2	*0.04*
	Exploration	Latency to Approach Aversive Stimulus	WT	280.5	*65.9*
			HET	92.4	*33.1*
		Sniffing (frequency)	WT	258.3	*23.7*
			HET	278.1	*22.4*
		Unsupported Rearing Time (s)	WT	73.7	*16.1*
			HET	72.1	*16.5*
	Activity	Freezing Time (s)	WT	6.2	*2.1*
			HET	3.7	*0.8*
		Abrupt Movement Frequency	WT	7.4	*2.5*
			HET	12.4	*5.2*
		Velocity	WT	2.4	*0.1*
			HET	2.7	*0.2*
		Locomotion Bursts (frequency)	WT	78.5	*13.4*
			HET	118.0	*26.4*
		Short Steps (frequency)	WT	20.3	*2.2*
			HET	26.6	*2.6*
