# Supplementary material for: Comprehensive Analysis of the 16p11.2 Deletion and Null Cntnap2 Mouse Models of Autism Spectrum Disorder
Source: PLoS One. 2015 Aug 14;10(8):e0134572. doi: 10.1371/journal.pone.0134572 (PMC4537259; doi:10.1371/journal.pone.0134572)
Supplement: S6 Table — (PDF) [file pone.0134572.s021.pdf]

S6 Table. SmartCube results for the Cntnap2 knockout model.

Cntnap2					
		Measure	Genotype	Mean	*SE*
SmartCube	Repetitive Behavior	Grooming Time	WT	193.9	*21.6*
			KO	185.6	*20.1*
		Digging Time (s)	WT	0.3	*0.04*
			KO	0.2	*0.03*
	Exploration	Latency to Approach Aversive Stimulus	WT	36.0	*7.4*
			KO	13.5	*2.8*
		Sniffing (frequency)	WT	446.9	*29.3*
			KO	396.1	*25.4*
		Unsupported Rearing Time (s)	WT	72.5	*7.1*
			KO	65.2	*9.1*
	Activity	Freezing Time (s)	WT	1.8	*0.3*
			KO	0.4	*0.1*
		Abrupt Movement Frequency	WT	6.8	*1.3*
			KO	30.7	*5.4*
		Velocity	WT	3.3	*0.1*
			KO	3.8	*0.1*
		Locomotion Bursts (frequency)	WT	236.6	*14.9*
			KO	358.5	*21.7*
		Short Steps (frequency)	WT	42.4	*3.4*
			KO	62.8	*2.9*
