# Supplementary material for: Comprehensive Analysis of the 16p11.2 Deletion and Null Cntnap2 Mouse Models of Autism Spectrum Disorder
Source: PLoS One. 2015 Aug 14;10(8):e0134572. doi: 10.1371/journal.pone.0134572 (PMC4537259; doi:10.1371/journal.pone.0134572)
Supplement: S7 Table — (PDF) [file pone.0134572.s022.pdf]

S7 Table. NeuroCube results for the 16p11.2 deletion model.

16p11.2		Genotype	P30 Mean	P30 *SE*	P60 Mean	P60 *SE*
Length and Width	Stride Length (mm)	WT	63.7	*1.5*	68.5	*1.3*
		HET	60.3	*2.3*	69.3	*2.3*
	Front Base (mm)	WT	16.3	*0.3*	17.3	*0.4*
		HET	16.0	*0.4*	16.3	*0.3*
	Hind Base (mm)	WT	24.3	*0.3*	23.8	*0.6*
		HET	26.7	*0.6*	25.0	*0.5*
Movement	Speed	WT	199.2	*16.8*	201.6	*16.6*
		HET	205.3	*18.4*	262.5	*20.3*
	Stride Duration	WT	375.8	*24.9*	391.7	*24.0*
		HET	356.7	*28.8*	319.4	*15.2*
	Stance Duration	WT	164.7	*11.2*	176.4	*14.2*
		HET	152.1	*15.9*	135.4	*9.9*
	Swing Percent	WT	55.7	*1.2*	54.9	*1.4*
		HET	57.7	*1.4*	57.7	*1.4*
Paw Measurements	Front Paw Area	WT	211.4	*3.3*	214.4	*3.8*
		HET	188.9	*3.7*	188.8	*4.3*
	Hind Paw Area	WT	150.3	*5.1*	178.6	*5.9*
		HET	140.5	*2.3*	155.7	*7.5*
	Front Paw Intensity	WT	80.9	*2.5*	84.4	*1.9*
		HET	80.3	*2.7*	84.6	*1.7*
	Hind Paw Intensity	WT	100.7	*2.6*	101.7	*2.8*
		HET	87.2	*2.7*	98.6	*2.2*
Weight & Bias	Body Weight	WT	21.1	*0.4*	26.0	*0.4*
		HET	17.2	*0.5*	22.2	*0.6*
	Hind Weight Bias	WT	1.2	*0.02*	1.2	*0.02*
		HET	1.1	*0.03*	1.2	*0.03*
	Left Hind Weight Bias	WT	1.1	*0.03*	1.1	*0.02*
		HET	1.0	*0.02*	1.1	*0.03*
	Left Front Weight Bias	WT	1.0	*0.01*	1.0	*0.01*
		HET	1.0	*0.02*	1.0	*0.01*
