# Supplementary material for: Comprehensive Analysis of the 16p11.2 Deletion and Null Cntnap2 Mouse Models of Autism Spectrum Disorder
Source: PLoS One. 2015 Aug 14;10(8):e0134572. doi: 10.1371/journal.pone.0134572 (PMC4537259; doi:10.1371/journal.pone.0134572)
Supplement: S8 Table — (PDF) [file pone.0134572.s023.pdf]

**S8 Table. NeuroCube results for the Cntnap2 knockout model.**

| Cntnap2 | | Genotype | P30 | | P60 | |
|---|---|---|---|---|---|---|
| | | | Mean | *SE* | Mean | *SE* |
| Length and Width | Stride Length (mm) | WT | 55.4 | *1.0* | 62.9 | *1.4* |
| | | KO | 55.0 | *0.5* | 63.0 | *1.1* |
| | Front Base (mm) | WT | 14.8 | *0.3* | 15.3 | *0.2* |
| | | KO | 14.4 | *0.3* | 15.1 | *0.3* |
| | Hind Base (mm) | WT | 25.0 | *0.4* | 25.6 | *0.4* |
| | | KO | 23.0 | *0.2* | 23.7 | *0.3* |
| Movement | Speed | WT | 187.8 | *9.8* | 209.2 | *12.7* |
| | | KO | 233.3 | *7.5* | 266.1 | *10.8* |
| | Stride Duration | WT | 337.6 | *16.3* | 333.0 | *11.3* |
| | | KO | 256.6 | *6.2* | 264.9 | *8.4* |
| | Stance Duration | WT | 132.8 | *7.6* | 136.4 | *5.4* |
| | | KO | 100.9 | *3.6* | 102.4 | *4.1* |
| | Swing Percent | WT | 60.5 | *1.3* | 58.9 | *0.8* |
| | | KO | 60.7 | *1.1* | 61.2 | *1.0* |
| Paw Measurements | Front Paw Area | WT | 165.8 | *3.3* | 175.6 | *4.3* |
| | | KO | 166.7 | *4.8* | 177.8 | *4.7* |
| | Hind Paw Area | WT | 138.9 | *3.2* | 154.8 | *4.5* |
| | | KO | 143.4 | *2.5* | 152.3 | *3.8* |
| | Front Paw Intensity | WT | 69.6 | *1.0* | 79.0 | *1.1* |
| | | KO | 65.7 | *1.2* | 75.2 | *1.0* |
| | Hind Paw Intensity | WT | 78.3 | *1.5* | 92.0 | *1.4* |
| | | KO | 73.8 | *2.2* | 88.4 | *2.3* |
| Weight & Bias | Body Weight | WT | 15.8 | *0.4* | 22.3 | *0.5* |
| | | KO | 14.9 | *0.6* | 21.5 | *0.3* |
| | Hind Weight Bias | WT | 1.1 | *0.02* | 1.2 | *0.02* |
| | | KO | 1.1 | *0.02* | 1.2 | *0.02* |
| | Left Hind Weight Bias | WT | 1.1 | *0.02* | 1.1 | *0.03* |
| | | KO | 1.1 | *0.04* | 1.1 | *0.04* |
| | Left Front Weight Bias | WT | 1.0 | *0.01* | 1.0 | *0.01* |
| | | KO | 1.0 | *0.01* | 1.0 | *0.01* |
