# Supplementary material for: Comprehensive Analysis of the 16p11.2 Deletion and Null Cntnap2 Mouse Models of Autism Spectrum Disorder
Source: PLoS One. 2015 Aug 14;10(8):e0134572. doi: 10.1371/journal.pone.0134572 (PMC4537259; doi:10.1371/journal.pone.0134572)
Supplement: S9 Table — (PDF) [file pone.0134572.s024.pdf]

S9 Table. General health data for the 16p11.2 deletion model.

16p11.2 — Health/General

Measure	Genotype	Mean	SE	Mean	SE	Mean	SE	n	Factor	Genotype	Age	Genotype x Age
Body Weight		P4		P7		P15						
	WT	3.5	*0.1*	5.2	*0.1*	9.1	*0.1*	16	F[1]	33.0	1009.8	14.9
	HET	2.9	*0.1*	4.4	*0.2*	7.4	*0.2*	16	*p*	0.0001	0.0001	0.0001
		P30		P60		P90						
	WT	20.4	*0.3*	26.3	*0.4*	30.0	*0.5*	32	F[1]	48.7	758.8	3.8
	HET	17.4	*0.3*	23.1	*0.4*	25.9	*0.5*	32	*p*	0.0001	0.0001	0.03
		P4		P7		P15						
Basal Temperature	WT	35.5	*0.1*	35.4	*0.1*	36.1	*0.1*	16	F	0.6	31.5	1.7
	HET	35.3	*0.1*	35.6	*0.1*	36.3	*0.2*	16	*p*	ns	0.0001	ns
Delta Temperature	WT	3.2	*0.3*	2.4	*0.1*	0.1	*0.1*	16	F	1.1	166.5	0.7
	HET	3.1	*0.2*	2.6	*0.1*	0.4	*0.2*	16	*p*	ns	0.0001	ns

Measure	Genotype	P4	P7	P15	P4 (n)	P7 (n)	P15 (n)	Test	P4	P7	P15
Milk	WT	100.0	68.8	-	16	16	16	Chi	4.57	0.0000	-
	HET	75.0	68.8	-	16	16	16	*p*	0.03	-	-

Measure	Genotype	P13 Right eye	P13 Left eye	n
Eye Opened P13 (%)	WT	93.8	93.8	16
	HET	93.8	87.5	16

Notes: [1]Post-hoc comparisons significant for the three ages
