# Supplementary material for: Comprehensive Analysis of the 16p11.2 Deletion and Null Cntnap2 Mouse Models of Autism Spectrum Disorder
Source: PLoS One. 2015 Aug 14;10(8):e0134572. doi: 10.1371/journal.pone.0134572 (PMC4537259; doi:10.1371/journal.pone.0134572)
Supplement: S10 Table — (PDF) [file pone.0134572.s025.pdf]

S10 Table. General health for the Cntnap2 knockout model.

Cntnap2										Factor			
Health/General	Measure	Genotype	Mean	SE	Mean	SE	Mean	SE	n		Genotype	Age	Genotype x Age
	Body Weight		P4		P7		P15						
		WT	2.1	*0.1*	3.5	*0.2*	6.5	*0.3*	22	**F**	0.6	921.2	0.2
		KO	2.2	*0.1*	3.7	*0.1*	6.7	*0.2*	22	***p***	ns	0.0001	ns
			P30		P60		P90						
		WT	15.0	*0.5*	23.1	*0.4*	26.1	*0.4*	29	**F**	1.8	1254.1	7.6
		KO	15.1	*0.4*	22.4	*0.3*	24.6	*0.3*	28	***p***	ns	0.0001	0.001
			P4		P7		P15						
	Basal Temperature	WT	35.2	*0.1*	35.3	*0.2*	36.1	*0.1*	22	**F**	0.1	32.0	0.4
		KO	35.2	*0.1*	35.4	*0.2*	36.1	*0.1*	22	***p***	ns	0.0001	ns
	Delta Temperature	WT	3.5	*0.1*	3.4	*0.2*	1.1	*0.1*	22	**F**	0.1	200.5	1.7
		KO	3.6	*0.2*	3.4	*0.2*	0.8	*0.1*	22	***p***	ns	0.0001	ns
									P4 (n) P7 (n) P15 (n)	Test	P4	P7	P15
	Milk	WT	86.4		72.7		-			Chi	0.003	1.9	-
		KO	87.0		65.2		-			***p*** [1]	ns	ns	-
			P13	Right eye	P13		Left eye		n				
	Eye Opened P13 (%)	WT	15.9				11.4		22				
		KO	19.5				20.5		22				

Notes: [1]Fisher exact p-value
