# Supplementary material for: Comprehensive Analysis of the 16p11.2 Deletion and Null Cntnap2 Mouse Models of Autism Spectrum Disorder
Source: PLoS One. 2015 Aug 14;10(8):e0134572. doi: 10.1371/journal.pone.0134572 (PMC4537259; doi:10.1371/journal.pone.0134572)
Supplement: S11 Table — (PDF) [file pone.0134572.s026.pdf]

S11 Table. Activity and ultrasonic vocalizations in the 16p11.2 deletion model.

16p11.2			P4		P7		P15			Factor			
	Measure	Genotype	Mean	*SE*	Mean	*SE*	Mean	*SE*	n		Genotype	Age	Genotype x Age
Activity	Square Crossing	WT	1.8	*0.6*	5.9	*0.8*	21.4	*5.6*	16	F	1.4	19.6	1.0
		HET	0.9	*0.4*	5.9	*2.0*	14.3	*2.9*	16	*p*	ns	0.0001	ns
	Pivot	WT	2.6	*0.6*	1.6	*0.6*	0.3	*0.2*	16	F	0.7	8.3	0.8
		HET	2.3	*0.7*	0.6	*0.3*	0.6	*0.2*	16	*p*	ns	0.001	ns
	Rear	WT	0.1	*0.1*	0.0	*0.0*	3.2	*0.9*	16	F	0.0001	P15 only	-
		HET	0.1	*0.1*	0.0	*0.0*	3.2	*0.8*	16	*p*	ns	-	-
	Groom	WT	0.0	*0.0*	0.0	*0.0*	1.3	*0.3*	16	F	2.1	P15 only	-
		HET	0.0	*0.0*	0.0	*0.0*	0.8	*0.3*	16	*p*	ns	-	-
Ultrasonic Vocalization		WT	64.3	*13.2*	142.1	*18.8*	4.7	*2.2*	16	F	0.0004	29.9	2.1
		HET	81.0	*17.7*	108.4	*21.5*	22.5	*11.2*	16	*p*	ns	0.0001	ns
