# Supplementary material for: Comprehensive Analysis of the 16p11.2 Deletion and Null Cntnap2 Mouse Models of Autism Spectrum Disorder
Source: PLoS One. 2015 Aug 14;10(8):e0134572. doi: 10.1371/journal.pone.0134572 (PMC4537259; doi:10.1371/journal.pone.0134572)
Supplement: S12 Table — (PDF) [file pone.0134572.s027.pdf]

S12 Table. Activity and ultrasonic vocalizations in the Cntnap2 knockout model.

Cntnap2														
	Measure	Genotype	P4		P7		P15		n	Factor				
			Mean	*SE*	Mean	*SE*	Mean	*SE*			Genotype	Age	Genotype x Age	
Activity	Square Crossing	WT	0.0	*0.0*	1.6	*0.7*	10.8	*3.4*	22	F	0.4	18.6	0.1	
		KO	0.2	*0.1*	2.9	*0.8*	12.2	*3.3*	22	*p*	ns	0.0001	ns	
	Pivot	WT	0.3	*0.1*	0.3	*0.2*	1.2	*0.4*	22	F	2.7	10.1	0.6	
		KO	0.6	*0.4*	0.5	*0.2*	2.0	*0.4*	22	*p*	ns	0.0001	ns	
	Rear	WT	0.0	*0.0*	0.0	*0.0*	0.9	*0.3*	22	F	0.5	P15 only	-	
		KO	0.0	*0.0*	0.0	*0.0*	1.2	*0.3*	22	*p*	ns	-	-	
	Groom	WT	0.0	*0.0*	0.0	*0.0*	1.2	*0.2*	22	F	1.2	P15 only	-	
		KO	0.0	*0.0*	0.0	*0.0*	0.9	*0.2*	22	*p*	ns	-	-	
Ultrasonic Vocalization		WT	6.0	*1.8*	7.3	*2.4*	23.9	*8.8*	22	F	1.1	5.0	1.5	
		KO	1.5	*0.5*	26.3	*10.0*	27.5	*10.5*	22	*p*	ns	0.01	ns	
