# Supplementary material for: Comprehensive Analysis of the 16p11.2 Deletion and Null Cntnap2 Mouse Models of Autism Spectrum Disorder
Source: PLoS One. 2015 Aug 14;10(8):e0134572. doi: 10.1371/journal.pone.0134572 (PMC4537259; doi:10.1371/journal.pone.0134572)
Supplement: S13 Table — (PDF) [file pone.0134572.s028.pdf]

S13 Table. Motor coordination and reflexes in the 16p11.2 deletion model.

16p11.2																
		Measure	Genotype	P4 Mean	P4 *SE*	P7 Mean	P7 *SE*	P15 Mean	P15 *SE*	n			Factor	Genotype	Age	Genotype x Age
Motor Coordination/Reflexes		Roll (Isolation Test)	WT	0.7	*0.4*	0.0	*0.0*	0.0	*0.0*	16			F	0.1	P4 only	-
			HET	0.6	*0.2*	0.0	*0.0*	0.0	*0.0*	16			*p*	ns	-	-
	Righting	Righting Latency	WT	3.3	*0.3*	2.5	*0.2*	1.0	*0.03*	16			F	6.9	39.2	3.6
			HET	5.3	*0.7*	3.5	*0.6*	1.0	*0.04*	16			*p*	0.01	0.0001	0.03
										P4 (n)	P7 (n)	P15 (n)	Test	P4	P7	P15
		Righting (#)	WT	2.9	*0.1*	3.0	*0.0*	3.0	*0.0*	16	16	16	U	78.0	120.0	-
			HET	2.4	*0.2*	2.9	*0.1*	3.0	*0.0*	16	16	16	*p*[1]	0.01	ns	-
	Geotaxis	Geotaxis fall (%)	WT	12.5		0.0		12.5		16	16	16	U	113.0	120.0	120.0
			HET	18.8		3.1		6.3		16	16	16	*p*[1]	ns	ns	ns
		Geotaxis turns (%)	WT	43.8		56.3		18.8		16	16	16	U	101.0	83.5	123.0
			HET	25.0		21.9		21.9		16	16	16	*p*[1]	ns	ns	ns
		Geotaxis walk down (%)	WT	0.0		0.0		68.8		16	16	16	U	-	-	125.0
			HET	0.0		0.0		75.0		16	16	16	*p*[1]	-	-	ns
		Latency to Fall	WT	29.3	*13.9*	-	-	5.7	*0.3*	3	0	2	F	0.1	-	-
			HET	32.5	*4.5*	5.0	-	2.0	-	5	1	1	*p*	ns	-	-
		Latency to Success	WT	34.8	*7.7*	44.0	*5.8*	19.0	*8.2*	8	9	3	F	4.2	0.1	-
			HET	56.0	*4.0*	47.3	*7.4*	9.0	*4.9*	5	4	4	*p*	ns	ns	-

Notes: Statistics were not performed if the observed number of subjects in a group was smaller than 3. [1]Mann-Whitney tied p-value
