# Supplementary material for: Comprehensive Analysis of the 16p11.2 Deletion and Null Cntnap2 Mouse Models of Autism Spectrum Disorder
Source: PLoS One. 2015 Aug 14;10(8):e0134572. doi: 10.1371/journal.pone.0134572 (PMC4537259; doi:10.1371/journal.pone.0134572)
Supplement: S14 Table — (PDF) [file pone.0134572.s029.pdf]

S14 Table. Motor coordination and reflexes in the Cntnap2 knockout model.

Cntnap2																
		**Measure**	**Genotype**	**P4 Mean**	**P4 *SE***	**P7 Mean**	**P7 *SE***	**P15 Mean**	**P15 *SE***	**n**			**Factor**	Genotype	Age	Genotype x Age
**Motor Coordination/Reflexes**		**Roll (Isolation Test)**	**WT**	2.1	*0.5*	0.1	*0.1*	0.0	*0.0*	22			**F**	4.1	P4 only	-
			**KO**	3.9	*0.7*	0.1	*0.1*	0.0	*0.0*	23			***p***	0.0497	-	-
	**Righting**	**Righting Latency**	**WT**	6.6	*0.6*	6.8	*0.9*	1.6	*0.0*	16			**F**	0.8	43.0	3.9
			**KO**	6.4	*0.7*	4.6	*0.4*	2.2	*0.5*	18			***p***	ns	0.0001	0.03
										**P4 (n)**	**P7 (n)**	**P15 (n)**	**Test**	**P4**	**P7**	**P15**
		**Righting (#)**	**WT**	1.4	*0.2*	2.3	*0.2*	2.9	*0.1*	22	22	22	**U**	235.0	162.0	231.0
			**KO**	1.3	*0.2*	1.7	*0.2*	3.0	*0.5*	23	23	22	***p***[1]	ns	0.03	ns
	**Geotaxis**	**Geotaxis fall (%)**	**WT**	25.0		11.4		4.5		22	22	22	**U**	233.0	204.0	231.0
			**KO**	30.4		21.7		0.0		23	23	22	***p***[1]	ns	ns	ns
		**Geotaxis turns (%)**	**WT**	6.8		9.1		72.7		22	22	22	**U**	231.0	244.0	196.0
			**KO**	15.2		10.9		56.8		23	23	22	***p***[1]	ns	ns	ns
		**Geotaxis walk down (%)**	**WT**	0.0		0.0		13.6		22	22	22	**U**	-	-	158.0
			**KO**	0.0		2.2		43.2		23	23	22	***p***[1]	-	-	0.02
		**Latency to Fall**	**WT**	25.8	*6.8*	30.8	*14.0*	53.0	-	9	3	1	**F**	0.01	0.4	-
			**KO**	27.0	*6.7*	22.4	*6.1*	-	-	10	8	0	***p***	ns	ns	-
		**Latency to Success**	**WT**	34.0	*26.0*	60.0	*0.0*	25.6	*4.4*	2	2	16	**F**	-	-	1.2
			**KO**	49.5	*10.5*	37.7	*15.1*	18.4	*5.0*	4	3	14	***p***	-	-	ns

Notes: Statistics were not performed if the observed number of subjects in a group was smaller than 3. [1]Mann-Whitney tied p-value
