# Supplementary material for: Comprehensive Analysis of the 16p11.2 Deletion and Null Cntnap2 Mouse Models of Autism Spectrum Disorder
Source: PLoS One. 2015 Aug 14;10(8):e0134572. doi: 10.1371/journal.pone.0134572 (PMC4537259; doi:10.1371/journal.pone.0134572)
Supplement: S15 Table — (PDF) [file pone.0134572.s030.pdf]

S15 Table. 3-chamber test for the 16p11.2 deletion model.

16p11.2

Test	Category	Measure	Genotype	Mean	*SE*	Mean	*SE*	n		Genotype	Stimulus	Genotype x Stimulus
				Left		Right				Factor		
3-Chamber		Baseline % Time in Chambers	**WT**	21.8	*3.1*	31.5	*3.8*	16	**F**	2.7	8.4	0.2
3-Chamber		Baseline % Time in Chambers	**HET**	28.3	*1.9*	35.6	*3.2*	16	***p***	ns	0.01	ns
				**Mouse**		**Object**						
3-Chamber	Social Preference	% Time in Chamber	**WT**	55.9	*5.0*	30.7	*3.5*	16	**F**	0.8	24.6	0.7
3-Chamber	Social Preference	% Time in Chamber	**HET**	59.1	*3.9*	29.8	*3.3*	16	***p***	ns	0.0001	ns
3-Chamber	Social Preference	% Time Sniffing	**WT**	24.0	*2.5*	8.0	*1.3*	16	**F**	0.001	53.9	0.1
3-Chamber	Social Preference	% Time Sniffing	**HET**	24.8	*2.2*	7.3	*1.0*	16	***p***	ns	0.0001	ns
3-Chamber	Social Preference	Entries to Chamber	**WT**	19.7	*7.4*	11.4	*1.2*	16	**F**	0.4	3.0	0.2
3-Chamber	Social Preference	Entries to Chamber	**HET**	15.4	*2.1*	10.8	*1.0*	16	***p***	ns	ns	ns
				**Mean**	***SE***					**Genotype**		
3-Chamber	Social Preference	Mouse Preference Time (%)	**WT**	63.0	*5.3*			16	**F**	0.3		
3-Chamber	Social Preference	Mouse Preference Time (%)	**HET**	66.1	*3.9*			16	***p***	ns		
3-Chamber	Social Preference	Mouse Sniffing (%)	**WT**	72.3	*5.9*			16	**F**	0.3		
3-Chamber	Social Preference	Mouse Sniffing (%)	**HET**	76.1	*3.4*			16	***p***	ns		
				**Novel**		**Familiar**		**n**		**Genotype**	**Stimulus**	**Genotype x Stimulus**
3-Chamber	Novel Mouse Preference	% Time in Chamber	**WT**	51.3	*2.8*	36.4	*2.6*	16	**F**	0.3	15.3	0.3
3-Chamber	Novel Mouse Preference	% Time in Chamber	**HET**	49.9	*2.1*	38.7	*2.2*	16	***p***	ns	0.001	ns
3-Chamber	Novel Mouse Preference	% Time Sniffing	**WT**	16.2	*1.8*	9.2	*1.0*	16	**F**	0.4	20.9	0.1
3-Chamber	Novel Mouse Preference	% Time Sniffing	**HET**	14.9	*1.3*	9.0	*0.8*	16	***p***	ns	0.0001	ns
3-Chamber	Novel Mouse Preference	Entries into Chamber	**WT**	30.1	*9.4*	28.4	*12.2*	16	**F**	0.3	0.2	1.4
3-Chamber	Novel Mouse Preference	Entries into Chamber	**HET**	21.4	*3.7*	25.0	*3.5*	16	***p***	ns	ns	ns
				**Mean**	***SE***					**Genotype**		
3-Chamber	Novel Mouse Preference	Novel Mouse Preference (%)	**WT**	58.5	*2.9*			16	**F**	0.3		
3-Chamber	Novel Mouse Preference	Novel Mouse Preference (%)	**HET**	56.4	*2.3*			16	***p***	ns		
3-Chamber	Novel Mouse Preference	Novel Mouse Sniffing (%)	**WT**	62.7	*3.7*			16	**F**	0.04		
3-Chamber	Novel Mouse Preference	Novel Mouse Sniffing (%)	**HET**	61.7	*3.2*			16	***p***	ns		
