# Supplementary material for: Comprehensive Analysis of the 16p11.2 Deletion and Null Cntnap2 Mouse Models of Autism Spectrum Disorder
Source: PLoS One. 2015 Aug 14;10(8):e0134572. doi: 10.1371/journal.pone.0134572 (PMC4537259; doi:10.1371/journal.pone.0134572)
Supplement: S16 Table — (PDF) [file pone.0134572.s031.pdf]

S16 Table. 3-chamber test for the Cntnap2 knockout model.

Cntnap2												
		Measure	Genotype	Mean Left	*SE* Left	Mean Right	*SE* Right	n	Factor	Genotype	Stimulus	Genotype x Stimulus
3-Chamber		Baseline % Time in Chambers	WT	38.1	*2.4*	36.4	*1.9*	13	F	5.3	1.2	0.1
			KO	36.5	*1.2*	33.1	*1.5*	13	*p*	0.03	ns	ns
				Mouse		Object						
	Social Preference	% Time in Chamber	WT	51.2	*2.4*	35.6	*1.8*	13	F	7.1	22.9	0.9
			KO	46.1	*2.0*	35.6	*1.9*	13	*p*	0.01	0.0001	ns
		% Time Sniffing	WT	20.8	*1.4*	8.9	*0.7*	13	F	11.7	110.7	1.1
			KO	15.7	*1.4*	6.0	*0.6*	13	*p*	0.002	0.0001	ns
		Entries to Chamber	WT	17.4	*1.4*	15.9	*1.1*	13	F	3.0	4.6	0.6
			KO	22.4	*2.7*	19.2	*1.9*	13	*p*	ns	0.04	ns
				Mean		*SE*				Genotype		
		Mouse Preference Time (%)	WT	58.8		*2.3*		13	F	0.6		
			KO	56.4		*2.2*		13	*p*	ns		
		Mouse Sniffing (%)	WT	69.4		*2.5*		13	F	1.0		
			KO	72.3		*1.6*		13	*p*	ns		
				Novel		Familiar		n		Genotype	Stimulus	Genotype x Stimulus
	Novel Mouse Preference	% Time in Chamber	WT	51.1	*1.6*	33.7	*1.7*	13	F	7.6	23.9	2.0
			KO	44.0	*2.7*	34.4	*2.3*	13	*p*	0.01	0.0001	ns
		% Time Sniffing	WT	16.1	*1.3*	7.3	*1.0*	13	F	10.1	37.5	3.7
			KO	10.4	*1.3*	5.8	*0.7*	13	*p*	0.004	0.0001	ns
		Entries into Chamber	WT	21.3	*1.7*	18.8	*1.8*	13	F	2.3	4.9	0.1
			KO	24.8	*2.2*	23.0	*2.1*	13	*p*	ns	0.04	ns
				Mean		*SE*				Genotype		
		Novel Mouse Preference (%)	WT	60.3		*1.7*		13	F	1.7		
			KO	55.9		*3.0*		13	*p*	ns		
		Novel Mouse Sniffing (%)	WT	68.8		*3.4*		13	F	1.3		
			KO	63.6		*3.0*		13	*p*	ns		
