# Supplementary material for: Comprehensive Analysis of the 16p11.2 Deletion and Null Cntnap2 Mouse Models of Autism Spectrum Disorder
Source: PLoS One. 2015 Aug 14;10(8):e0134572. doi: 10.1371/journal.pone.0134572 (PMC4537259; doi:10.1371/journal.pone.0134572)
Supplement: S17 Table — (PDF) [file pone.0134572.s032.pdf]

S17 Table. Reciprocal social interaction test for the 16p11.2 deletion model, same genotype stimulus.

16p11.2								
		Genotype	Mean	*SE*	*n*		
Reciprocal Social Interaction Test: Homogeneous Design	Distance between subjects (cm)	WT	15.6	*0.7*	16	F	5.8
		HET	13.3	*0.6*	16	*p*	0.02
	Distance moved (both mice, cm)	WT	3564.2	*227.5*	16	F	0.8
		HET	3493.9	*158.4*	16	*p*	ns
	Time in 5 cm Proximity (s)	WT	34.0	*2.4*	16	F	3.0
		HET	39.7	*2.2*	16	*p*	ns
	Time nose-nose interaction (both mice, s)	WT	32.2	*5.1*	16	F	0.4
		HET	36.0	*2.7*	16	*p*	ns
	Time nose-center interaction (both mice, s)	WT	27.6	*4.7*	16	F	2.7
		HET	38.3	*4.5*	16	*p*	ns
	Time nose-tail interaction (both mice, s)	WT	51.6	*4.2*	16	F	6.2
		HET	70.8	*6.4*	16	*p*	0.02
	All nose interactions (both mice, s)	WT	111.4	*10.8*	16	F	4.3
		HET	145.1	*12.1*	16	*p*	0.046
	Follow (s)	WT	57.5	*2.2*	16	F	0.01
		HET	55.4	*2.1*	16	*p*	ns
	Active Social (%)	WT	13.6	*1.7*	16	F	0.1
		HET	14.2	*1.5*	16	*p*	ns
	Passive Social (%)	WT	9.3	*1.3*	16	F	0.5
		HET	10.6	*1.2*	16	*p*	ns
	Reciprocal Social (%)	WT	9.6	*1.1*	16	F	0.003
		HET	9.7	*1.0*	16	*p*	ns
	Total Social (%)	WT	32.6	*2.3*	16	F	0.4
		HET	34.5	*2.1*	16	*p*	ns
	Active Social (#/min)	WT	6.2	*0.4*	16	F	0.003
		HET	6.3	*0.5*	16	*p*	ns
	Passive Social (#/min)	WT	4.5	*0.6*	16	F	0.6
		HET	5.1	*0.4*	16	*p*	ns
	Reciprocal Social (#/min)	WT	4.6	*0.3*	16	F	0.5
		HET	4.9	*0.4*	16	*p*	ns
	Total Social (#/min)	WT	15.3	*0.8*	16	F	0.7
		HET	16.3	*0.8*	16	*p*	ns
	Ultrasonic vocalizations	WT	0.4	*0.8*	16	F[1]	-
		HET	1.3	*1.1*	16	*p*	-

Note. [1]Vocalizations were very infrequent (less than 1.5 in the 10 min test) and thus were not analyzed statistically.
