# Supplementary material for: Comprehensive Analysis of the 16p11.2 Deletion and Null Cntnap2 Mouse Models of Autism Spectrum Disorder
Source: PLoS One. 2015 Aug 14;10(8):e0134572. doi: 10.1371/journal.pone.0134572 (PMC4537259; doi:10.1371/journal.pone.0134572)
Supplement: S18 Table — (PDF) [file pone.0134572.s033.pdf]

S18 Table. Reciprocal social interaction test for the 16p11.2 deletion model, WT stimulus.

	**16p11.2**						
Reciprocal Social Interaction Test: Heterogeneous Design		**Genotype**	**Mean**	***SE***	***n***		
	**Distance between subjects (cm)**	**WT**	16.6	*0.6*	16	**F**	3.1
		**HET**	14.8	*0.8*	16	***p***	ns
	**Distance moved (both mice, cm)**	**WT**	4501.3	*198.6*	16	**F**	0.9
		**HET**	4721.2	*125.8*	16	***p***	ns
	**Time in 5 cm Proximity (s)**	**WT**	30.9	*2.3*	16	**F**	1.3
		**HET**	35.2	*3.0*	16	***p***	ns
	**Time nose-nose interaction (both mice, s)**	**WT**	34.8	*3.7*	16	**F**	0.002
		**HET**	35.0	*4.7*	16	***p***	ns
	**Time nose-center interaction (both mice, s)**	**WT**	27.3	*2.7*	16	**F**	0.2
		**HET**	29.9	*4.6*	16	***p***	ns
	**Time nose-tail interaction (both mice, s)**	**WT**	52.1	*4.2*	16	**F**	0.2
		**HET**	54.8	*5.1*	16	***p***	ns
	**All nose interactions (both mice, s)**	**WT**	114.2	*8.5*	16	**F**	0.1
		**HET**	119.8	*13.2*	16	***p***	ns
	**Follow (s)**	**WT**	68.7	*2.3*	16	**F**	1.0
		**HET**	71.7	*1.7*	16	***p***	ns
	**Active Social (%)**	**WT**	14.8	*1.8*	16	**F**	2.3
		**HET**	18.6	*1.9*	16	***p***	ns
	**Passive Social (%)**	**WT**	8.1	*1.1*	16	**F**	0.3
		**HET**	7.3	*0.9*	16	***p***	ns
	**Reciprocal Social (%)**	**WT**	10.9	*1.5*	16	**F**	0.2
		**HET**	10.2	*1.2*	16	***p***	ns
	**Total Social (%)**	**WT**	33.8	*2.9*	16	**F**	0.3
		**HET**	36.1	*2.6*	16	***p***	ns
	**Active Social (#/min)**	**WT**	6.9	*0.6*	16	**F**	1.2
		**HET**	7.9	*0.7*	16	***p***	ns
	**Passive Social (#/min)**	**WT**	4.6	*0.5*	16	**F**	0.5
		**HET**	4.1	*0.4*	16	***p***	ns
	**Reciprocal Social (#/min)**	**WT**	5.3	*0.5*	16	**F**	0.02
		**HET**	5.2	*0.5*	16	***p***	ns
	**Total Social (#/min)**	**WT**	16.9	*1.1*	16	**F**	0.1
		**HET**	17.3	*12.0*	16	***p***	ns
