# Supplementary material for: Comprehensive Analysis of the 16p11.2 Deletion and Null Cntnap2 Mouse Models of Autism Spectrum Disorder
Source: PLoS One. 2015 Aug 14;10(8):e0134572. doi: 10.1371/journal.pone.0134572 (PMC4537259; doi:10.1371/journal.pone.0134572)
Supplement: S19 Table — (PDF) [file pone.0134572.s034.pdf]

S19 Table. Reciprocal social interaction test for the Cntnap2 knockout model, same genotype stimulus.

Cntnap2							
		**Genotype**	**Mean**	***SE***	***n***		
Reciprocal Social Interaction Test: Homogeneous Design	**Distance between subjects (cm)**	**WT**	16.3	*0.5*	14	**F**	0.03
		**KO**	16.1	*0.6*	16	***p***	ns
	**Distance moved (both mice, cm)**	**WT**	5306.2	*226.9*	14	**F**	2.7
		**KO**	5810.2	*209.0*	16	***p***	ns
	**Time in 5 cm Proximity (s)**	**WT**	28.4	*2.2*	14	**F**	0.1
		**KO**	29.3	*1.8*	16	***p***	ns
	**Time nose-nose interaction (both mice, s)**	**WT**	23.4	*3.3*	14	**F**	0.1
		**KO**	22.3	*1.4*	16	***p***	ns
	**Time nose-center interaction (both mice, s)**	**WT**	30.7	*2.7*	14	**F**	0.1
		**KO**	31.8	*3.1*	16	***p***	ns
	**Time nose-tail interaction (both mice, s)**	**WT**	43.7	*3.3*	14	**F**	0.01
		**KO**	43.2	*4.3*	16	***p***	ns
	**All nose interactions (both mice, s)**	**WT**	97.8	*6.3*	14	**F**	0.003
		**KO**	97.3	*6.6*	16	***p***	ns
	**Follow (s)**	**WT**	75.0	*2.7*	14	**F**	2.3
		**KO**	80.5	*2.4*	16	***p***	ns
	**Active Social (s)**	**WT**	15.8	*2.0*	14	**F**	0.5
		**KO**	14.0	*1.6*	16	***p***	ns
	**Passive Social (s)**	**WT**	6.7	*0.7*	14	**F**	0.1
		**KO**	6.4	*1.0*	16	***p***	ns
	**Reciprocal Social (s)**	**WT**	6.2	*0.5*	14	**F**	0.2
		**KO**	5.8	*0.7*	16	***p***	ns
	**Total Social (s)**	**WT**	28.7	*2.1*	14	**F**	1.0
		**KO**	26.1	*1.5*	16	***p***	ns
	**Active Social (#/min)**	**WT**	7.6	*0.4*	14	**F**	0.9
		**KO**	8.3	*0.6*	16	***p***	ns
	**Passive Social (#/min)**	**WT**	4.9	*0.5*	14	**F**	0.01
		**KO**	5.0	*0.6*	16	***p***	ns
	**Reciprocal Social (#/min)**	**WT**	5.0	*0.5*	14	**F**	0.6
		**KO**	4.6	*0.4*	16	***p***	ns
	**Total Social (#/min)**	**WT**	17.6	*1.0*	14	**F**	0.1
		**KO**	17.9	*0.8*	16	***p***	ns
	**Ultrasonic vocalizations**	**WT**	0.8	*0.4*	14	**F1**	-
		**KO**	1.1	*0.6*	16	***p***	-

Note. [1]Vocalizations were very infrequent (less than 1.5 in the 10 min test) and thus were not analyzed statistically.
