# Supplementary material for: Comprehensive Analysis of the 16p11.2 Deletion and Null Cntnap2 Mouse Models of Autism Spectrum Disorder
Source: PLoS One. 2015 Aug 14;10(8):e0134572. doi: 10.1371/journal.pone.0134572 (PMC4537259; doi:10.1371/journal.pone.0134572)
Supplement: S20 Table — (PDF) [file pone.0134572.s035.pdf]

S20 Table. Reciprocal social interaction test for the Cntnap2 knockout model, WT stimulus.

Cntnap2								
		Genotype	Mean	*SE*	*n*		
Reciprocal Social Interaction Test: Heterogeneous Design	Distance between subjects (cm)	WT	16.6	*0.8*	16	F	0.02
		KO	16.7	*0.5*	16	*p*	ns
	Distance moved (both mice, cm)	WT	5701.2	*236.8*	16	F	2.0
		KO	6225.9	*282.6*	16	*p*	ns
	Time in 5 cm Proximity (s)	WT	30.6	*2.8*	16	F	0.1
		KO	29.4	*2.1*	16	*p*	ns
	Time nose-nose interaction (both mice, s)	WT	29.8	*3.9*	16	F	0.1
		KO	28.6	*2.5*	16	*p*	ns
	Time nose-center interaction (both mice, s)	WT	26.3	*2.9*	16	F	1.3
		KO	21.8	*2.7*	16	*p*	ns
	Time nose-tail interaction (both mice, s)	WT	44.7	*4.3*	16	F	0.02
		KO	43.7	*5.3*	16	*p*	ns
	All nose interactions (both mice, s)	WT	100.8	*9.7*	16	F	0.3
		KO	94.2	*9.1*	16	*p*	ns
	Follow (s)	WT	82.1	*3.0*	16	F	2.3
		KO	89.1	*3.5*	16	*p*	ns
	Active Social (s)	WT	16.8	*2.5*	16	F	0.2
		KO	18.1	*1.2*	16	*p*	ns
	Passive Social (s)	WT	5.7	*0.8*	16	F	0.003
		KO	5.7	*0.9*	16	*p*	ns
	Reciprocal Social (s)	WT	8.1	*0.9*	16	F	0.6
		KO	7.1	*0.9*	16	*p*	ns
	Total Social (s)	WT	30.7	*3.2*	16	F	0.01
		KO	30.9	*1.7*	16	*p*	ns
	Active Social (#/min)	WT	7.4	*0.8*	16	F	2.8
		KO	9.0	*0.5*	16	*p*	ns
	Passive Social (#/min)	WT	3.7	*0.4*	16	F	1.3
		KO	4.3	*0.4*	16	*p*	ns
	Reciprocal Social (#/min)	WT	5.1	*0.4*	16	F	1.3
		KO	5.7	*0.4*	16	*p*	ns
	Total Social (#/min)	WT	16.2	*1.2*	16	F	4.0
		KO	19.0	*0.8*	16	*p*	ns
