# Supplementary material for: Comprehensive Analysis of the 16p11.2 Deletion and Null Cntnap2 Mouse Models of Autism Spectrum Disorder
Source: PLoS One. 2015 Aug 14;10(8):e0134572. doi: 10.1371/journal.pone.0134572 (PMC4537259; doi:10.1371/journal.pone.0134572)
Supplement: S21 Table — (PDF) [file pone.0134572.s036.pdf]

S21 Table. Urine exposure open field test for the 16p11.2 deletion model

16p11.2								
		Measure	Genotype	Mean	*SE*	n		
Urine Open Field	Baseline	Distance Traveled (cm)	WT	23895.8	*9588.4*	16	F	0.1
			HET	19803.4	*5104.6*	16	*p*	ns
		Center Time (%)	WT	5.9	*1.2*	16	F	8.2
			HET	16.7	*3.6*	16	*p*	0.008
		Center Distance (cm)	WT	2444.1	*870.3*	16	F	2.1
			HET	4716.7	*1300.6*	16	*p*	ns
		Scent Marking (#)	WT	21.8	*6.7*	16	F	0.5
			HET	28.9	*7.8*	16	*p*	ns
		Scent Marking (Pixel)	WT	194530.3	*66640.6*	16	F	0.003
			HET	199224.9	*47835.5*	16	*p*	ns
	Urine Exposure	Distance Traveled (cm)	WT	741.1	*58.7*	16	F	4.6
			HET	930.2	*66.2*	16	*p*	0.041
		Center Time (%)	WT	13.0	*1.2*	16	F	2.5
			HET	16.0	*1.4*	16	*p*	ns
		Center Distance (cm)	WT	131.2	*18.2*	16	F	3.3
			HET	203.1	*35.4*	16	*p*	ns
		Scent Marking (#)	WT	12.6	*4.5*	16	F	1.0
			HET	20.0	*5.8*	16	*p*	ns
		Scent Marking (Pixel)	WT	44881.6	*17432.0*	16	F	1.3
			HET	75448.9	*19715.8*	16	*p*	ns
		Ultrasonic Vocalization (#)	WT	74.0	*39.0*	16	F	1.2
			HET	150.2	*57.5*	16	*p*	ns
