# Supplementary material for: Comprehensive Analysis of the 16p11.2 Deletion and Null Cntnap2 Mouse Models of Autism Spectrum Disorder
Source: PLoS One. 2015 Aug 14;10(8):e0134572. doi: 10.1371/journal.pone.0134572 (PMC4537259; doi:10.1371/journal.pone.0134572)
Supplement: S22 Table — (PDF) [file pone.0134572.s037.pdf]

S22 Table. Urine exposure open field test for the Cntnap2 knockout model.

Cntnap2								
		Measure	Genotype	Mean	*SE*	n		
Urine Open Field	Baseline	Distance Traveled (cm)	WT	23022.0	*5107.7*	16	F	1.6
			KO	15247.2	*3279.0*	16	*p*	ns
		Center Time (%)	WT	17.4	*1.5*	16	F	0.03
			KO	17.0	*1.8*	16	*p*	ns
		Center Distance (cm)	WT	5520.6	*1115.4*	16	F	1.4
			KO	3946.8	*730.6*	16	*p*	ns
		Scent Marking (#)	WT	40.4	*9.3*	16	F	3.9
			KO	19.5	*5.2*	16	*p*	0.06
		Scent Marking (Pixel)	WT	303645.5	*67223.1*	16	F	5.3
			KO	130758.0	*32725.3*	16	*p*	0.03
	Urine Exposure	Distance Traveled (cm)	WT	974.5	*52.8*	16	F	21.2
			KO	1251.2	*25.2*	16	*p*	0.001
		Center Time (%)	WT	17.9	*1.6*	16	F	0.4
			KO	16.5	*1.5*	16	*p*	ns
		Center Distance (cm)	WT	181.6	*20.2*	16	F	2.1
			KO	218.0	*16.9*	16	*p*	ns
		Scent Marking (#)	WT	31.5	*9.0*	16	F	2.4
			KO	15.7	*5.0*	16	*p*	ns
		Scent Marking (Pixel)	WT	114182.8	*37762.3*	16	F	1.0
			KO	67047.9	*27579.9*	16	*p*	ns
		Ultrasonic Vocalization (#)	WT	454.1	*101.0*	16	F	6.9
			KO	152.3	*54.0*	16	*p*	0.013
