# Supplementary material for: Comprehensive Analysis of the 16p11.2 Deletion and Null Cntnap2 Mouse Models of Autism Spectrum Disorder
Source: PLoS One. 2015 Aug 14;10(8):e0134572. doi: 10.1371/journal.pone.0134572 (PMC4537259; doi:10.1371/journal.pone.0134572)
Supplement: S23 Table — (PDF) [file pone.0134572.s038.pdf]

S23 Table. T-maze test for the 16p11.2 deletion model.

16p11.2									
T-Maze		Measure	Genotype	Proportion		n			
	Acquisition	Proportion to Criterion	WT	0.9		16	Chi Square		3.7
			HET	1.0		16	*p*		ns
				Mean	*SE*				
		Days to Criterion	WT	3.3	*0.3*	16	t		-1.7
			HET	2.8	*0.3*	16	*p*		ns
	Reversal			Proportion					
		Proportion to Criterion	WT	0.9		15	Chi Square		0.02
			HET	0.9		16	*p*		ns
				Mean	*SE*				
		Days to Criterion	WT	3.4	*0.3*	15	t		-0.3
			HET	3.2	*0.1*	16	*p*		ns
				n		Genotype	Session	Genotype x Session	
		Percent Correct	WT	15	F	2.0	51.9	1.8	
			HET	16	*p*	ns	0.0001	ns	
