# Supplementary material for: Comprehensive Analysis of the 16p11.2 Deletion and Null Cntnap2 Mouse Models of Autism Spectrum Disorder
Source: PLoS One. 2015 Aug 14;10(8):e0134572. doi: 10.1371/journal.pone.0134572 (PMC4537259; doi:10.1371/journal.pone.0134572)
Supplement: S24 Table — (PDF) [file pone.0134572.s039.pdf]

S24 Table. T-maze test for the Cntnap2 knockout model.

Cntnap2								
T-Maze		Measure	Genotype	Proportion		n		
	Acquisition	Proportion to Criterion	WT	1.0		13	Chi Square	6.0
			KO	1.0		13	*p*	0.01
				Mean	*SE*			
		Days to Criterion	WT	3.6	*0.3*	13	t	-2.7
			KO	2.7	*0.1*	13	*p*	0.01
	Reversal			Proportion				
		Proportion to Criterion	WT	1.0		13	Chi Square	0.0001
			KO	1.0		13	*p*	-
				Mean	*SE*			
		Days to Criterion	WT	2.9	*0.1*	13	t	0.0001
			KO	2.9	*0.1*	13	*p*	-
				n		Genotype	Session	Genotype x Session
		Percent Correct	WT	13	F	0.2	89.7	0.6
			KO	13	*p*	ns	0.0001	ns
