# Supplementary material for: Comprehensive Analysis of the 16p11.2 Deletion and Null Cntnap2 Mouse Models of Autism Spectrum Disorder
Source: PLoS One. 2015 Aug 14;10(8):e0134572. doi: 10.1371/journal.pone.0134572 (PMC4537259; doi:10.1371/journal.pone.0134572)
Supplement: S25 Table — (PDF) [file pone.0134572.s040.pdf]

S25 Table. Startle and prepulse inhibition of startle for the 16p11.2 deletion model.

16p11.2

Measure	Genotype	Mean	*SE*	n		
Startle	WT	629.2	*100.6*	16	F	1.9
	HET	468.5	*60.7*	16	*p*	ns

Measure	Genotype	74		78		82		n		Genotype	Prepulse Level	Genotype x Level
		Mean	*SE*	Mean	*SE*	Mean	*SE*					
Prepulse Inhibition	WT	26.4	*4.7*	39.3	*3.8*	43.8	*6.6*	16	F	0.01	36.1	1.8
	HET	22.5	*3.5*	36.7	*4.9*	48.9	*3.9*	16	*p*	ns	0.0001	ns
