# Supplementary material for: Comprehensive Analysis of the 16p11.2 Deletion and Null Cntnap2 Mouse Models of Autism Spectrum Disorder
Source: PLoS One. 2015 Aug 14;10(8):e0134572. doi: 10.1371/journal.pone.0134572 (PMC4537259; doi:10.1371/journal.pone.0134572)
Supplement: S26 Table — (PDF) [file pone.0134572.s041.pdf]

S26 Table. Startle and prepulse inhibition of startle for the Cntnap2 knockout model.

Cntnap2

Measure	Genotype	Mean	*SE*	n		
Startle	WT	723.7	*56.5*	16	F	0.2
	KO	696.6	*33.0*	16	*p*	ns

Measure	Genotype	74		78		82		n		Genotype	Prepulse Level	Genotype x Level
		Mean	*SE*	Mean	*SE*	Mean	*SE*					
Prepulse Inhibition	WT	10.6	*3.9*	21.7	*4.1*	30.1	*5.0*	16	F	6.5	53.2	0.01
	KO	24.6	*4.6*	35.8	*4.2*	44.6	*3.5*	16	*p*	0.02	0.0001	ns
