# Supplementary material for: Comprehensive Analysis of the 16p11.2 Deletion and Null Cntnap2 Mouse Models of Autism Spectrum Disorder
Source: PLoS One. 2015 Aug 14;10(8):e0134572. doi: 10.1371/journal.pone.0134572 (PMC4537259; doi:10.1371/journal.pone.0134572)
Supplement: S27 Table — (PDF) [file pone.0134572.s042.pdf]

S27 Table. Marble burying test for the 16p11.2 deletion model.

16p11.2							
	**Measure**	**Genotype**	**Mean**	***SE***	**n**		
**Marble-Burying**	**Marbles Buried**	**WT**	14.1	*1.3*	16	**F**	0.01
		**HET**	14.3	*1.6*	16	***p***	ns
	**Total Distance**	**WT**	7201.3	*434.4*	16	**F**	5.0
		**HET**	8769.6	*542.7*	15	***p***	0.03
