# Supplementary material for: Comprehensive Analysis of the 16p11.2 Deletion and Null Cntnap2 Mouse Models of Autism Spectrum Disorder
Source: PLoS One. 2015 Aug 14;10(8):e0134572. doi: 10.1371/journal.pone.0134572 (PMC4537259; doi:10.1371/journal.pone.0134572)
Supplement: S28 Table — (PDF) [file pone.0134572.s043.pdf]

S28 Table. Marble burying test for the Cntnap2 knockout model.

Cntnap2							
	**Measure**	**Genotype**	**Mean**	***SE***	**n**		
**Marble-Burying**	**Marbles Buried**	**WT**	14.8	*1.1*	16	***F***	3.0
		**KO**	11.8	*1.3*	16	***p***	ns
	**Total Distance**	**WT**	7720.1	*361.7*	16	***F***	0.1
		**KO**	7833.0	*324.0*	15	***p***	ns
