# Supplementary material for: Comprehensive Analysis of the 16p11.2 Deletion and Null Cntnap2 Mouse Models of Autism Spectrum Disorder
Source: PLoS One. 2015 Aug 14;10(8):e0134572. doi: 10.1371/journal.pone.0134572 (PMC4537259; doi:10.1371/journal.pone.0134572)
Supplement: S30 Table — (PDF) [file pone.0134572.s045.pdf]

3-Chamber Test

Number of mice	Genotype	Habituation - Left Side Time (%)	Habituation - Center Time (%)	Habituation - Right Time (%)	Sociability - Mouse Time (%)	Sociability - Center Time (%)	Sociability - Object Time (%)	Sociability - Mouse Sniff Time	Sociability - Object Sniff Time	Recognition - Familiar Mouse Time (%)	Recognition - Center Time (%)	Recognition - Novel Mouse Time (%)	Recognition - Familiar Mouse Sniff Time	Recognition - Novel Mouse Sniff Time	Sociability - Crossings to Mouse Side (#)	Sociability - Crossings to Object Side (#)	Recognition - Crossings to Familiar Mouse (#)	Recognition - Crossings to Novel Mouse (#)
1	16p11.2 WT	33.135	37.528	29.337	76.3	9.693	14.0	188.9	28.7	30.3	3.788	65.9	71.0	179.8	39.0	9.0	14.0	11.0
2	16p11.2 WT	31.97	33.364	34.666	61.0	11.6	27.4	132.4	14.5	34.2	18.47	47.4	33.8	51.5	27.0	19.0	209.0	166.0
3	16p11.2 WT	27.61	31.17	41.22	63.3	7.382	29.3	170.9	42.4	28.1	7.39	64.5	44.7	186.7	13.0	14.0	26.0	11.0
4	16p11.2 WT	26.202	50.052	23.746	2.1	40.681	57.2	0.0	88.3	16.0	13.154	70.8	26.2	129.7	12.0	20.0	23.0	30.0
5	16p11.2 WT	20.014	39.211	40.775	78.9	7.339	13.7	208.3	18.6	36.0	13.506	50.5	38.6	62.3	10.0	8.0	11.0	18.0
6	16p11.2 WT	0	100	0	79.6	5.967	14.5	263.7	28.8	56.5	12.162	31.4	89.0	61.2	4.0	6.0	12.0	33.0
7	16p11.2 WT	10.71	44.538	44.752	67.0	10.332	22.7	168.3	53.5	42.5	17.398	40.1	73.9	83.2	9.0	4.0	12.0	9.0
8	16p11.2 WT	23.854	33.155	42.991	29.9	12.435	57.7	90.2	142.1	30.9	22.867	46.2	62.9	47.6	6.0	9.0	15.0	18.0
9	16p11.2 WT	0	93.702	6.298	42.3	17.511	40.2	120.1	78.0	53.1	9.642	37.3	109.2	71.5	4.0	9.0	10.0	9.0
10	16p11.2 WT	29.476	24.036	46.488	66.9	7.873	25.2	132.4	41.0	45.7	10.792	43.5	36.1	68.1	7.0	8.0	9.0	21.0
11	16p11.2 WT	31.055	24.618	44.327	48.6	6.424	45.0	104.9	27.2	27.5	9.775	62.8	35.4	80.1	12.0	9.0	14.0	14.0
12	16p11.2 WT	16.946	28.622	54.431	62.4	10.186	27.4	163.2	41.5	40.4	10.05	49.5	47.6	77.5	15.0	17.0	16.0	24.0
13	16p11.2 WT	9.65	75.996	14.355	52.2	17.24	30.6	113.7	44.5	29.5	12.108	58.3	18.7	140.5	8.0	8.0	7.0	15.0
14	16p11.2 WT	16.004	53.128	30.868	57.1	20.91	21.9	161.1	22.1	44.2	11.003	44.8	84.6	83.5	9.0	17.0	19.0	35.0
15	16p11.2 WT	44.102	30.984	24.915	41.7	18.331	40.0	97.8	56.1	35.4	13.15	51.5	48.8	88.8	126.0	13.0	28.0	37.0
16	16p11.2 WT	28.591	46.325	25.084	65.7	9.368	25.0	189.9	39.3	31.3	12.004	56.7	60.1	140.9	14.0	13.0	29.0	30.0
1	16p11.2 df/+	24.628	35.351	40.021	49.1	19.681	31.3	94.3	32.4	40.2	5.764	54.1	68.3	130.1	12.0	13.0	17.0	17.0
2	16p11.2 df/+	31.135	32.14	36.725	49.4	10.912	39.7	133.1	41.8	27.8	10.172	62.1	44.7	155.6	31.0	20.0	20.0	21.0
3	16p11.2 df/+	27.341	30.318	42.342	59.2	8.005	32.8	121.6	74.9	31.8	8.268	59.9	39.6	127.8	15.0	12.0	56.0	63.0
4	16p11.2 df/+	26.984	37.289	35.726	32.0	18.334	49.6	72.1	89.1	25.2	19.047	55.7	30.0	104.9	15.0	11.0	23.0	24.0
5	16p11.2 df/+	31.772	27.882	40.346	50.3	11.248	38.5	142.0	39.2	33.2	16.879	50.0	44.4	89.1	12.0	12.0	31.0	27.0
6	16p11.2 df/+	42.496	57.504	0	76.0	4.468	19.6	206.5	32.3	30.3	17.807	51.9	66.8	106.1	4.0	5.0	12.0	16.0
7	16p11.2 df/+	29.907	47.943	22.15	53.2	12.259	34.6	144.0	52.9	44.9	14.002	41.1	98.6	59.1	20.0	12.0	37.0	11.0
8	16p11.2 df/+	29.588	33.147	37.265	48.5	11.045	40.5	140.8	56.8	43.6	15.625	40.8	52.4	36.2	20.0	15.0	27.0	22.0
9	16p11.2 df/+	30.04	26.768	43.192	56.4	12.905	30.7	154.8	48.4	48.2	9.036	42.8	37.5	55.3	27.0	11.0	24.0	12.0
10	16p11.2 df/+	16.344	33.291	50.365	67.9	9.361	22.7	121.4	30.8	25.7	6.237	68.0	26.3	71.4	4.0	8.0	4.0	9.0
11	16p11.2 df/+	13.191	57.645	29.164	43.9	10.757	45.3	114.5	81.8	44.6	11.896	43.5	87.4	68.6	6.0	8.0	25.0	6.0
12	16p11.2 df/+	32.927	31.906	35.167	61.9	7.097	31.0	139.7	59.2	52.7	6.208	41.1	37.9	91.4	6.0	7.0	8.0	12.0
13	16p11.2 df/+	24.658	25.929	49.412	52.5	11.678	35.8	149.4	34.3	42.0	15.342	42.6	66.7	71.8	26.0	17.0	36.0	20.0
14	16p11.2 df/+	22.025	25.044	52.931	83.0	5.119	11.9	214.1	19.3	37.5	8.951	53.6	55.5	104.5	12.0	8.0	15.0	19.0
15	16p11.2 df/+	26.94	45.777	27.283	67.8	19.644	12.6	131.1	10.1	42.5	10.305	47.2	49.3	60.3	18.0	7.0	48.0	49.0
16	16p11.2 df/+	42.862	29.297	27.841	94.4	5.299	0.3	298.1	0.0	48.9	7.373	43.8	54.7	96.4	18.0	6.0	17.0	15.0
1	Cntnap2 WT	40.157	27.245	32.598	34.3	18.961	46.7	79.3	52.2	25.8	12.458	61.7	22.7	138.0	23.0	18.0	20.0	21.0
2	Cntnap2 WT	45.009	22.418	32.573	53.4	13.208	33.4	125.9	50.0	44.3	13.669	42.1	95.4	56.4	21.0	11.0	26.0	24.0
3	Cntnap2 WT	31.742	34.383	33.875	68.8	8.94	22.2	163.0	40.6	37.0	12.656	50.3	30.9	103.7	13.0	11.0	23.0	19.0
4	Cntnap2 WT	44.868	18.576	36.556	44.7	19.598	35.7	97.8	44.3	26.1	26.587	47.3	36.0	73.6	10.0	11.0	17.0	28.0
5	Cntnap2 WT	51.344	18.598	30.058	44.9	10.136	45.0	82.5	91.1	40.4	12.423	47.2	34.2	61.6	26.0	17.0	18.0	17.0
6	Cntnap2 WT	42.519	23.207	34.275	62.4	7.169	30.4	136.1	36.8	32.2	8.64	59.2	45.8	114.6	10.0	22.0	17.0	15.0
7	Cntnap2 WT	50.49	21.848	27.662	51.2	7.478	41.3	145.7	37.3	42.4	6.507	51.1	80.4	98.0	15.0	16.0	11.0	12.0
8	Cntnap2 WT	29.309	20.126	50.566	45.6	21.646	32.8	96.6	57.0	28.4	19.769	51.8	31.3	82.2	16.0	16.0	15.0	26.0
9	Cntnap2 WT	29.068	34.06	36.872	54.2	15.495	30.3	126.1	54.3	33.8	18.585	47.6	42.6	82.2	22.0	17.0	12.0	16.0
10	Cntnap2 WT	36.577	18.866	44.557	57.3	9.721	33.0	178.2	62.7	34.7	13.643	51.6	43.1	122.0	17.0	13.0	15.0	15.0
11	Cntnap2 WT	41.588	29.247	29.164	50.7	10.663	38.6	135.9	59.8	29.2	13.471	57.3	34.4	111.0	20.0	14.0	18.0	28.0
12	Cntnap2 WT	27.314	29.141	43.545	50.5	11.523	37.9	144.5	67.5	33.4	23.495	43.1	28.5	68.9	20.0	18.0	35.0	31.0
13	Cntnap2 WT	25.545	33.895	40.56	47.2	17.601	35.2	110.8	44.0	30.3	15.172	54.5	42.3	141.5	13.0	23.0	17.0	25.0
1	Cntnap2 -/-	39.49	25.22	35.291	39.8	17.375	42.8	77.4	19.7	38.5	17.473	44.1	24.8	35.7	13.0	12.0	18.0	18.0
2	Cntnap2 -/-	35.458	29.446	35.096	42.3	9.683	48.0	59.2	33.9	31.9	30.611	37.5	21.5	30.2	22.0	19.0	29.0	32.0
3	Cntnap2 -/-	37.932	23.109	38.959	31.3	23.91	44.8	66.9	27.4	18.6	26.368	55.0	13.9	61.8	23.0	24.0	21.0	30.0
4	Cntnap2 -/-	39.982	38.894	21.125	43.1	18.622	38.3	99.6	58.2	33.1	19.293	47.6	52.4	78.8	26.0	16.0	26.0	25.0
5	Cntnap2 -/-	38.066	25.468	36.466	48.0	15.085	36.9	103.7	47.4	42.2	21.009	36.8	47.4	54.7	19.0	15.0	17.0	17.0
6	Cntnap2 -/-	30.441	34.596	34.963	53.1	18.191	28.8	123.7	26.1	35.5	17.399	47.1	51.4	96.7	24.0	18.0	17.0	21.0
7	Cntnap2 -/-	28.866	34.509	36.625	46.5	22.504	31.0	68.5	28.5	23.9	15.318	60.8	23.9	103.7	12.0	15.0	14.0	18.0
8	Cntnap2 -/-	44.815	30.891	24.295	56.8	15.296	27.9	143.4	51.9	51.2	21.255	27.6	55.3	36.7	13.0	10.0	16.0	14.0
9	Cntnap2 -/-	38.199	32.756	29.045	51.3	17.831	30.9	97.2	41.9	41.3	15.621	43.1	34.6	63.1	28.0	26.0	18.0	20.0
10	Cntnap2 -/-	35.631	33.217	31.153	42.2	17.711	40.1	77.2	30.8	33.7	23.133	43.2	20.6	43.1	25.0	22.0	33.0	39.0
11	Cntnap2 -/-	36.841	31.041	32.118	43.0	26.753	30.3	50.2	16.2	35.5	36.949	27.5	12.5	22.0	49.0	37.0	38.0	30.0
12	Cntnap2 -/-	38.162	26.615	35.223	57.8	12.611	29.6	142.5	50.4	31.9	19.968	48.2	48.0	78.7	21.0	19.0	29.0	36.0
13	Cntnap2 -/-	31.041	28.816	40.143	44.6	21.518	33.9	118.1	36.6	30.1	16.897	53.0	46.5	106.9	16.0	17.0	23.0	23.0
