# Supplementary material for: Comprehensive Analysis of the 16p11.2 Deletion and Null Cntnap2 Mouse Models of Autism Spectrum Disorder
Source: PLoS One. 2015 Aug 14;10(8):e0134572. doi: 10.1371/journal.pone.0134572 (PMC4537259; doi:10.1371/journal.pone.0134572)
Supplement: S31 Table — (PDF) [file pone.0134572.s046.pdf]

Reciprocal Social Interaction

Number of mice	Genotype	Total USV #	SAME GENOTYPE STIMULUS: Distance between subjects	Distance moved (both mice)	Time in 5 cm Proximity	Time nose-nose interaction (the pair)	Time nose-center interaction (both mice)	Time nose-tail interaction (both mice)	Frequency nose-nose interaction (the pair)	Frequency nose-center interaction (both mice)	Frequency nose-tail interaction (both mice)	Total approach	Follow	Active Social (%)	Passive Social (%)	Reciprocal Social (%)	WILD TYPE STIMULUS: Distance between subjects	Distance moved (both mice)	Time in 5 cm Proximity	Time nose-nose interaction (the pair)	Time nose-center interaction (both mice)	Time nose-tail interaction (both mice)	Frequency nose-nose interaction (the pair)	Frequency nose-center interaction (both mice)	Frequency nose-tail interaction (both mice)	Total approach	Follow	Active Social (%)	Passive Social (%)	Reciprocal Social (%)
1	16p11.2 WT	0	13	3924	39	40	31	38	48	36	65	5370	62	11.72	6.03	4.84	15	5125	38	46	27	61	55	47	88	6148	78	15.63	7.77	16.03
2	16p11.2 WT	0	16	3523	23	16	25	45	38	50	71	4687	54	13.30	9.48	3.91	21	6107	18	18	23	27	37	54	63	6969	85	9.09	4.78	4.71
3	16p11.2 WT	0	15	3055	42	56	29	81	24	36	89	4120	48	24.42	1.21	6.72	16	3428	23	25	18	53	29	27	56	4510	55	12.29	7.58	11.18
4	16p11.2 WT	2	14	2736	40	38	89	78	42	68	76	4234	48	26.17	9.35	11.56	12	4159	47	54	34	73	77	75	117	5964	67	21.83	4.13	16.03
5	16p11.2 WT	0	20	3978	30	28	12	58	46	42	77	4959	60	17.33	6.46	9.93	16	3353	33	49	21	62	51	43	67	4800	55	13.37	11.93	18.59
6	16p11.2 WT	0	17	3371	24	17	19	62	34	35	84	4748	53	14.23	5.96	9.67	16	4636	31	39	32	55	35	54	97	5757	69	11.54	13.98	14.79
7	16p11.2 WT	0	16	4724	29	29	29	55	43	56	82	5716	70	13.17	5.35	5.43	18	4331	30	60	21	46	41	44	67	5558	63	22.13	3.61	4.97
8	16p11.2 WT	0	15	3822	25	15	25	41	27	31	74	5268	60	6.31	18.02	10.03	21	3879	15	7	16	51	14	17	59	4733	60	11.28	1.53	1.37
9	16p11.2 WT	0	13	3683	39	34	28	48	33	56	90	5183	59	10.13	14.84	11.23	17	4975	31	41	24	38	60	50	77	6302	74	15.75	3.25	6.93
10	16p11.2 WT	4	11	4379	54	22	14	56	60	55	115	5791	65	9.54	16.78	10.78	20	4218	24	23	22	39	34	30	60	5427	65	13.32	5.73	4.04
11	16p11.2 WT	0	14	2486	39	28	21	54	42	25	58	3821	42	15.77	12.85	13.55	14	3828	37	34	38	49	56	63	89	5327	61	25.74	12.58	8.20
12	16p11.2 WT	0												7.59	3.24	4.40	18	5496	20	12	8	18	31	36	46	5977	78	4.88	8.29	5.53
13	16p11.2 WT	0	15	3593	34	33	33	63	33	50	92	4908	57	7.51	15.60	7.37	16	4137	42	32	26	79	37	46	93	5552	65	4.84	12.77	11.51
14	16p11.2 WT	0	13	4439	47	94	41	46	66	70	85	5430	64	12.34	10.72	16.97	15	3957	40	35	41	49	49	65	81	5392	66	14.74	14.88	19.56
15	16p11.2 WT	0	20	2982	22	31	29	41	30	42	53	4008	48	3.26	10.89	8.43	16	4785	29	44	53	54	58	59	89	5593	70	10.14	12.64	14.30
16	16p11.2 WT	0	15	4964	35	31	9	53	63	30	98	5849	71	25.48	2.63	18.94	14	5605	39	37	32	79	57	71	121	6515	85	29.88	4.20	17.32
1	16p11.2 df/+	2	13	3883	37	23	56	58	35	67	87	5121	59	10.37	5.35	6.22	8	4946	60	78	65	93	82	108	133	6100	76	19.42	15.09	17.95
2	16p11.2 df/+	0	15	2955	31	28	34	40	41	50	68	4288	47	10.92	6.88	4.74	12	4970	40	38	39	48	75	62	79	5769	75	27.04	5.53	13.19
3	16p11.2 df/+	1	11	3700	32	30	19	56	42	41	85	4835	54	13.66	13.57	4.74	16	4331	18	8	13	37	19	42	81	5636	65	21.19	2.80	3.71
4	16p11.2 df/+	0	11	2409	57	55	51	107	42	57	127	3581	39	12.40	20.01	14.87	17	5499	26	28	21	43	49	47	67	6831	80	10.13	7.99	6.22
5	16p11.2 df/+	5	10	4126	46	41	47	88	52	70	123	5547	63	28.57	6.74	9.99	14	5605	32	31	25	57	45	64	115	7156	86	22.16	7.16	8.95
6	16p11.2 df/+	0	14	2897	42	42	56	122	45	73	81	4252	48	7.43	9.75	15.57	12	4660	53	36	10	35	44	28	77	5660	70	17.15	6.98	5.86
7	16p11.2 df/+	0	15	3277	39	42	38	64	58	57	83	4719	54	17.52	5.29	15.88	18	4011	27	49	35	53	52	61	88	5750	65	11.23	5.41	16.93
8	16p11.2 df/+	0	19	3640	24	14	12	31	23	26	47	5132	58	7.00	7.28	8.54	14	4451	26	17	7	39	22	26	76	5430	65	6.62	11.72	9.32
9	16p11.2 df/+	0	15	4607	38	36	29	68	44	41	117	5826	67	10.84	11.89	7.74	21	4407	23	30	20	44	34	36	55	5657	68	13.94	6.44	10.20
10	16p11.2 df/+	4	15	4023	32	29	23	41	39	51	83	5854	66	15.31	7.55	5.40	12	4410	50	64	56	102	99	113	131	5831	68	33.83	10.74	13.62
11	16p11.2 df/+	6	13	3754	37	25	41	71	57	76	92	4966	56	10.26	13.03	6.36	12	5154	35	25	22	59	49	68	127	6340	79	22.64	10.75	13.72
12	16p11.2 df/+	0	12	3347	41	37	19	85	45	41	94	4730	52	11.60	18.84	7.46	18	4202	29	14	19	55	22	46	82	5495	64	6.82	8.62	5.41
13	16p11.2 df/+	0	14	3957	35	46	17	52	46	53	93	5700	65	14.88	7.52	10.56	17	5534	30	15	42	50	41	70	105	6768	80	19.10	6.65	5.89
14	16p11.2 df/+	1	9	2276	58	31	46	60	43	35	76	3782	43	10.39	16.42	16.00	14	4409	40	36	12	39	57	37	67	5893	70	18.24	7.58	15.48
15	16p11.2 df/+	0	13	3187	44	51	80	100	49	84	116	4569	52	19.97	14.18	9.55	14	4605	45	46	62	80	63	113	160	5804	69	23.77	1.31	11.86
16	16p11.2 df/+	1	15	3865	42	47	46	88	55	75	121	5249	61	25.88	5.72	11.36	18	4345	26	46	29	41	54	50	83	5443	65	24.64	2.33	4.19
1	Cntnap2 WT	1	17	5625	30	22	36	38	46	79	83	6656	79	9.10	8.24	7.78	16	5202	30	26	19	53	41	35	88	6496	76	12.66	6.02	6.97
2	Cntnap2 WT	2	16	3955	30	39	26	34	49	51	66	5243	60	13.28	7.17	7.82	15	4070	41	47	48	67	46	50	84	5208	61	12.48	11.95	11.36
3	Cntnap2 WT	0	19	5129	16	11	14	25	22	39	53	6265	74	4.23	6.36	3.38	15	5792	31	30	37	47	61	83	108	7185	85	12.81	10.16	6.59
4	Cntnap2 WT	0	17	3945	28	14	40	40	24	52	76	5196	59	15.93	5.13	4.53	18	6565	25	21	11	35	50	38	60	7123	91	3.99	5.37	5.67
5	Cntnap2 WT	5	13	5199	48	59	14	42	80	51	106	6474	77	36.20	7.34	7.32	8	5353	61	56	39	69	106	104	161	6848	80	45.66	6.10	13.90
6	Cntnap2 WT	0	17	6255	17	14	20	25	32	56	77	7232	84	16.56	3.88	3.94	15	6790	31	32	15	49	61	48	108	7939	98	25.52	1.24	6.87
7	Cntnap2 WT	0	18	6559	19	21	26	42	59	62	88	7320	90	15.86	8.21	5.98	14	4825	45	69	30	80	75	70	110	6102	70	21.90	7.01	11.72
8	Cntnap2 WT	1	13	4746	33	25	32	54	47	56	102	5888	67	16.22	5.68	5.77	17	5795	32	28	27	41	65	71	107	6723	85	25.51	1.82	11.42
9	Cntnap2 WT	0	15	6235	30	24	43	56	52	85	119	7283	89	9.98	6.50	9.91	15	5859	33	23	48	57	60	66	113	6603	81	15.15	6.51	13.74
10	Cntnap2 WT	0	16	6095	30	23	33	42	47	57	101	6860	80	12.31	9.10	6.10	16	5963	30	20	27	40	54	68	86	7019	85	14.85	2.83	4.48
11	Cntnap2 WT	2	17	5332	25	16	37	48	35	58	99	6693	76	24.18	1.45	4.68	18	5038	24	19	20	32	48	50	75	6363	75	13.20	4.66	6.35
12	Cntnap2 WT	0	19	5924	23	13	26	38	33	51	94	6885	82	15.06	7.60	4.62	20	7158	21	19	23	27	45	65	88	7537	99	21.22	4.35	3.41
13	Cntnap2 WT	0	16	4541	32	24	32	67	36	62	128	5635	66	19.32	5.14	8.11	19	7274	29	38	13	31	73	53	90	7987	103	17.28	11.91	10.84
14	Cntnap2 WT	0	15	4748	36	21	49	61	49	76	97	5621	67	13.17	12.60	6.26	20	5852	16	16	25	22	34	49	63	6758	82	5.99	3.88	3.07
15	Cntnap2 WT																18	5578	16	17	19	19	40	51	59	6701	81	8.49	3.02	6.69
16	Cntnap2 WT																20	4106	25	17	18	44	25	36	57	5268	62	12.64	5.06	6.39
1	Cntnap2 -/-	0	16	5169	32	28	39	44	53	69	99	6269	71	26.18	1.65	6.74	18	8390	25	29	19	31	67	61	102	8907	116	27.65	1.07	2.73
2	Cntnap2 -/-	0	15	6240	32	24	25	31	54	65	97	7099	81	17.72	6.78	8.09	19	8363	19	19	17	29	59	61	95	8877	115	14.63	1.96	4.05
3	Cntnap2 -/-	1	16	5218	26	15	30	48	42	58	103	6131	72	13.20	7.02	3.86	18	6504	23	31	13	28	60	55	66	7042	91	16.82	4.22	5.17
4	Cntnap2 -/-	1	15	4908	35	25	64	42	52	88	88	5983	70	11.66	10.55	9.16	18	6679	17	11	5	22	28	26	66	7290	94	11.25	7.48	4.10
5	Cntnap2 -/-	0	18	5496	24	23	28	32	51	48	84	6535	77	6.34	9.46	9.72	14	5692	31	21	25	42	43	55	97	6930	85	19.67	6.62	5.37
6	Cntnap2 -/-	0	18	5305	23	24	25	42	46	57	99	6652	79	13.36	3.78	4.67	19	4679	28	21	9	48	42	30	94	5939	70	12.91	5.22	8.25
7	Cntnap2 -/-	0	12	4590	44	25	19	59	55	44	120	5622	67	12.61	10.39	7.19	14	5313	41	47	24	50	81	69	98	6149	75	22.10	4.68	14.78
8	Cntnap2 -/-	7	20	5892	26	16	32	48	32	67	88	6853	84	9.05	6.22	4.45	18	6676	29	33	14	36	57	49	80	7182	92	19.81	4.64	5.39
9	Cntnap2 -/-	0	18	6417	26	16	27	34	49	69	90	6991	89	8.46	3.94	3.52	18	7119	23	21	17	32	50	57	99	7418	99	12.65	5.25	8.25
10	Cntnap2 -/-	0	19	6878	19	15	29	40	42	67	99	7260	91	18.91	1.77	3.37	18	6163	29	29	37	39	56	90	101	6520	88	18.91	7.43	8.34
11	Cntnap2 -/-	0	14	5067	37	22	54	87	36	78	129	6462	75	12.66	5.98	9.50	15	6615	27	41	15	35	76	53	93	7667	99	21.32	2.66	6.84
12	Cntnap2 -/-	0	15	5336	32	35	14	30	65	43	90	6169	74	7.03	16.04	9.06	18	5124	26	22	22	47	37	49	91	6505	76	13.49	4.62	4.94
13	Cntnap2 -/-	0	19	6339	18	24	27	20	44	44	59	7101	86	10.08	3.25	1.64	12	4842	47	39	42	94	63	85	156	6192	72	12.75	15.71	11.40
14	Cntnap2 -/-	0	16	7656	27	23	25	36	58	71	107	8207	103	14.51	3.62	5.31	16	5818	33	35	22	37	69	61	99	6790	85	25.42	3.52	13.01
15	Cntnap2 -/-	3	16	6755	27	14	31	28	39	87	100	7407	92	13.82	8.17	3.60	18	6637	29	18	25	34	49	82	92	7147	93	20.80	4.49	4.64
16	Cntnap2 -/-	6	12	5699	40	27	41	70	59	79	168	6809	77	28.72	3.26	2.15	13	4999	42	39	42	95	78	80	123	6206	75	20.01	11.25	6.74
