# Supplementary material for: Comprehensive Analysis of the 16p11.2 Deletion and Null Cntnap2 Mouse Models of Autism Spectrum Disorder
Source: PLoS One. 2015 Aug 14;10(8):e0134572. doi: 10.1371/journal.pone.0134572 (PMC4537259; doi:10.1371/journal.pone.0134572)
Supplement: S32 Table — (PDF) [file pone.0134572.s047.pdf]

Urine Open Field Test

Number of mice	Genotype	Total Distance Traveled (cm) - Baseline	Total Distance Traveled (cm) - Exposure	Time in Center (%) - Baseline	Time in Center (%) - Exposure	Distance in Center (cm) - Baseline	Distance in Center (cm) - Exposure	Marking Baseline #	Marking Exposure Session #	Marking Exposure in Center #	Marking Baseline Area	Marking Exposure Session Area	Marking Exposure in Center Area	Total Exposure USV #
1	16p11.2 WT	2859.4	626.9	1.2	9.8	162.6	108.1	0	1	0	0	11097	0	5
2	16p11.2 WT	7310.4	908.4	12.2	16.8	1658.4	153.1	1	2	1	5978	46388	1214	25
3	16p11.2 WT	81234.4	580.9	15.3	5.0	5228.2	8.9	30	12	0	312113	32925	0	1
4	16p11.2 WT	4733.6	629.8	3.8	23.8	904.7	161.6	25	5	1	121398	18545	2857	28
5	16p11.2 WT	131527.5	599.3	1.8	11.9	2749.5	130.9	4	3	2	122947	9458	2719	0
6	16p11.2 WT	75785.3	538.4	13.9	15.9	14255.8	95.9	32	53	5	376465	278514	57664	0
7	16p11.2 WT	2905.0	583.4	1.1	10.0	292.3	88.0	23	9	0	385785	15241	0	288
8	16p11.2 WT	5116.5	1034.1	4.3	18.0	516.6	227.2	4	7	4	16795	21571	10581	4
9	16p11.2 WT	34767.4	821.4	5.1	6.6	4355.2	28.9	108	38	4	1074058	85948	5987	56
10	16p11.2 WT	2327.8	429.9	2.1	10.0	570.7	62.4	2	2	0	8701	9857	0	0
11	16p11.2 WT	2273.9	524.7	0.6	18.8	134.3	103.8	7	3	0	13902	18473	0	0
12	16p11.2 WT	7015.9	1158.7	8.5	13.7	1965.6	237.1	25	3	0	100212	9783	0	145
13	16p11.2 WT	3617.5	622.6	4.9	10.8	1005.0	101.2	8	2	0	149427	12905	0	13
14	16p11.2 WT	4165.3	678.8	3.1	12.1	713.9	144.9	9	6	3	108112	18814	8118	13
15	16p11.2 WT	7250.5	941.2	6.9	16.9	2480.5	272.7	26	3	0	107696	7766	0	20
16	16p11.2 WT	9443.0	1179.6	9.1	8.6	2112.0	175.0	45	53	6	208896	120821	13896	586
1	16p11.2 df/+	5546.8	1373.5	24.3	23.2	2253.0	520.1	4	11	1	64777	39517	1578	32
2	16p11.2 df/+	842.4	511.5	0.1	5.2	61.3	37.6	4	21	0	289592	222585	0	0
3	16p11.2 df/+	45127.5	1286.2	43.3	19.5	12664.1	376.0	37	79	4	251987	209537	6610	164
4	16p11.2 df/+	6143.2	730.1	7.6	12.7	1512.6	128.6	2	13	2	16561	45806	5494	51
5	16p11.2 df/+	49129.5	1180.2	29.1	20.1	16854.4	324.3	1	6	2	8925	17451	7022	32
6	16p11.2 df/+	2488.4	583.3	2.8	10.2	288.1	63.7	5	23	1	92926	112780	2839	246
7	16p11.2 df/+	59936.8	1103.4	15.3	15.9	5790.5	322.9	10	3	1	92565	19141	4506	0
8	16p11.2 df/+	4145.6	649.4	4.3	15.0	776.3	101.6	45	38	2	318064	168055	6942	0
9	16p11.2 df/+	46555.7	1132.8	27.9	11.8	11575.8	151.9	59	67	9	257712	200488	42417	300
10	16p11.2 df/+	30243.0	1018.7	2.5	8.8	3356.0	47.0	11	5	0	39848	29981	0	162
11	16p11.2 df/+	32541.3	755.4	40.0	20.8	10147.9	122.8	3	4	1	73848	11023	1631	713
12	16p11.2 df/+	3806.0	600.8	3.9	15.2	517.7	145.6	30	8	1	102823	13906	1229	12
13	16p11.2 df/+	7386.4	988.1	19.2	17.4	2654.2	103.8	101	15	1	673422	36190	1254	0
14	16p11.2 df/+	8787.3	932.8	10.2	19.5	2418.2	258.7	68	2	0	325620	16221	0	20
15	16p11.2 df/+	8750.5	1102.8	32.0	26.9	3702.6	371.4	69	24	1	516193	49085	1678	662
16	16p11.2 df/+	5424.0	934.4	4.3	14.2	893.7	173.2	14	1	0	62736	15416	0	9
1	Cntnap2 WT	8515.5	1215.6	15.0	11.1	2485.1	280.2	25	47	0	226109	171955	0	0
2	Cntnap2 WT	52851.7	982.3	15.6	13.5	12107.4	165.7	20	11	0	100682	67168	0	6
3	Cntnap2 WT	8253.5	836.4	16.0	26.6	2322.5	167.6	2	13	0	11831	34756	0	992
4	Cntnap2 WT	9446.4	1081.0	18.2	24.4	2735.2	218.2	3	10	1	41491	53239	1450	756
5	Cntnap2 WT	11398.0	961.9	14.2	22.0	3488.8	211.6	16	84	2	284207	291805	7787	1127
6	Cntnap2 WT	10359.6	954.8	17.3	15.8	2578.1	143.9	58	105	5	885833	571327	7417	59
7	Cntnap2 WT	13098.2	991.4	22.1	17.7	3885.4	138.0	47	54	6	344521	186756	16980	568
8	Cntnap2 WT	8394.3	1485.5	23.2	31.4	2935.4	378.7	74	103	11	437667	242529	18004	268
9	Cntnap2 WT	8300.4	873.1	8.7	13.3	1585.5	128.6	21	2	1	177508	4687	2529	429
10	Cntnap2 WT	56454.1	1146.7	26.3	25.3	10538.2	266.9	14	18	3	54606	54622	13022	775
11	Cntnap2 WT	8043.7	806.1	13.9	21.5	2090.0	251.3	101	12	1	643797	23141	1044	40
12	Cntnap2 WT	54701.0	557.3	18.7	16.3	13692.3	115.5	60	13	3	594134	29459	5246	549
13	Cntnap2 WT	13154.8	727.3	10.6	12.5	3054.3	127.2	131	16	0	658898	40345	0	9
14	Cntnap2 WT	50495.2	988.9	32.2	14.2	11338.8	119.6	50	8	3	253214	34604	6005	937
15	Cntnap2 WT	8773.5	1079.7	14.8	12.2	2071.0	60.9	25	4	0	143830	7950	0	747
16	Cntnap2 WT	46111.7	904.3	12.4	9.3	11421.7	131.8	0	4	0	0	12581	0	3
1	Cntnap2 -/-	7749.4	1182.2	13.4	15.2	2027.6	130.2	33	46	3	344997	174615	24122	203
2	Cntnap2 -/-	14318.2	1232.9	22.1	28.7	4759.6	331.1	4	16	3	73351	69515	9056	4
3	Cntnap2 -/-	9871.5	1239.3	35.6	15.1	3814.5	214.2	3	11	1	10788	43041	5187	92
4	Cntnap2 -/-	10949.1	1198.9	14.9	22.5	2980.7	217.3	2	1	0	19163	7067	0	26
5	Cntnap2 -/-	10762.2	1271.0	22.1	24.1	3641.6	280.4	3	5	0	6297	15605	0	1
6	Cntnap2 -/-	11674.2	1316.0	9.1	10.2	2356.1	145.9	47	23	3	303773	53495	4342	0
7	Cntnap2 -/-	8483.5	1406.8	14.0	18.3	2229.8	232.8	70	74	4	402126	437667	10572	562
8	Cntnap2 -/-	12618.7	1409.2	14.4	8.9	3045.6	179.5	7	2	1	105071	5589	1223	191
9	Cntnap2 -/-	12236.2	1332.7	15.4	7.7	3372.6	241.1	12	3	0	70741	12135	0	32
10	Cntnap2 -/-	9979.3	1201.8	10.4	22.6	2678.0	322.0	0	1	0	0	8148	0	538
11	Cntnap2 -/-	5837.2	1269.1	11.2	19.0	1514.6	295.8	10	2	0	44031	8157	0	0
12	Cntnap2 -/-	8807.6	1324.3	12.3	16.8	2265.2	200.1	29	31	3	201255	126231	5423	10
13	Cntnap2 -/-		1220.7		18.1		176.6	12	21	1	108099	77190	11783	593
14	Cntnap2 -/-	44513.6	1255.6	27.7	7.9	9369.9	237.2	17	6	1	111629	12984	1398	0
15	Cntnap2 -/-	47513.4	1169.4	16.8	15.8	11698.0	196.7	13	3	0	29726	10844	0	29
16	Cntnap2 -/-	13393.6	989.0	16.3	13.5	3448.0	86.4	50	6	1	261081	10483	1277	155
