# Supplementary material for: Comprehensive Analysis of the 16p11.2 Deletion and Null Cntnap2 Mouse Models of Autism Spectrum Disorder
Source: PLoS One. 2015 Aug 14;10(8):e0134572. doi: 10.1371/journal.pone.0134572 (PMC4537259; doi:10.1371/journal.pone.0134572)
Supplement: S33 Table — (PDF) [file pone.0134572.s048.pdf]

T-Maze

Number of mice	Genotype	Acquisition - Days to Criterion	Reversal - Days to Criterion	Reversal - % Correct Day 1	Reversal - % Correct Day 2	Reversal - % Correct Day 3	Reversal - % Correct Day 4	Reversal - % Correct Day 5	Reversal - % Correct Day 6
1	16p11.2 WT	3	3	75	75	100	100	100	100
2	16p11.2 WT	2	2	62.5	100	100	100	87.5	87.5
3	16p11.2 WT	2	3	37.5	37.5	50	87.5	62.5	100
4	16p11.2 WT	3	3	25	25	87.5	100	87.5	100
5	16p11.2 WT	3	4	12.5	87.5	100	100	100	100
6	16p11.2 WT	3	3	25	75	100	100	100	100
7	16p11.2 WT	5	6	25	87.5	100	100	100	100
8	16p11.2 WT	3	4	37.5	87.5	100	100	100	100
9	16p11.2 WT	4	3	50	100	100	100	100	100
10	16p11.2 WT	3	2	37.5	75	100	100	100	100
11	16p11.2 WT	3	3	87.5	62.5	100	100	100	100
12	16p11.2 WT	3	3	50	100	87.5	100	100	100
13	16p11.2 WT	3	5	37.5	87.5	100	100	100	100
14	16p11.2 WT			12.5	100	87.5	100	100	100
15	16p11.2 WT	3	3	25	100	87.5	100	100	100
16	16p11.2 WT	7		75	100	100	100	100	100
1	16p11.2 df/+	2	3	37.5	87.5	100	100	100	100
2	16p11.2 df/+	2	3	37.5	75	100	100	100	100
3	16p11.2 df/+	3	3	75	100	100	100	100	100
4	16p11.2 df/+	2	3	50	100	100	100	100	100
5	16p11.2 df/+	3	3	12.5	75	100	100	100	100
6	16p11.2 df/+	3	3	50	87.5	100	100	100	100
7	16p11.2 df/+	6	4	75	87.5	62.5	100	100	100
8	16p11.2 df/+	3	3	62.5	75	100	100	87.5	100
9	16p11.2 df/+	2	4	25	50	87.5	75	87.5	100
10	16p11.2 df/+	4	4	87.5	87.5	100	100	100	100
11	16p11.2 df/+	3	3	62.5	87.5	100	100	87.5	100
12	16p11.2 df/+	3	3	37.5	100	100	100	100	87.5
13	16p11.2 df/+	3	4	37.5	100	100	100	100	100
14	16p11.2 df/+	2	2	62.5	87.5	100	87.5	100	100
15	16p11.2 df/+	2	3	37.5	87.5	100	100	100	100
16	16p11.2 df/+	2		62.5	100	100	100	100	100
1	Cntnap2 WT	3	3	25	50	87.5	100	100	100
2	Cntnap2 WT	3	3	25	87.5	100	100	100	100
3	Cntnap2 WT	2	3	0	75	100	100	100	100
4	Cntnap2 WT	5	3	75	100	87.5	100	100	100
5	Cntnap2 WT	3	3	37.5	100	75	100	100	100
6	Cntnap2 WT	5	2	37.5	100	100	100	100	100
7	Cntnap2 WT	5	2	0	12.5	12.5	25	87.5	87.5
8	Cntnap2 WT	3	3	12.5	62.5	87.5	100	100	100
9	Cntnap2 WT	2	3	50	100	100	100	100	100
10	Cntnap2 WT	3	4	0	25	12.5	75	87.5	100
11	Cntnap2 WT	4	3						
12	Cntnap2 WT	5	3	75	87.5	100	100	100	100
13	Cntnap2 WT	4	3	25	75	87.5	100	100	100
1	Cntnap2 -/-	3	3	25	100	100	100	100	100
2	Cntnap2 -/-	3	3	0	0	12.5	0	12.5	
3	Cntnap2 -/-	3	3	62.5	100	87.5	100	100	100
4	Cntnap2 -/-	3	3	62.5	87.5	100	100	100	100
5	Cntnap2 -/-	3	3	50	100	100	100	100	100
6	Cntnap2 -/-	3	3	25	100	100	100	100	100
7	Cntnap2 -/-	2	3	62.5	87.5	100	100	100	100
8	Cntnap2 -/-	3	3	62.5	100	100	100	100	100
9	Cntnap2 -/-	2	3	12.5	100	100	100	100	100
10	Cntnap2 -/-	2	2	25	62.5	100	100	100	100
11	Cntnap2 -/-	3	4	25	87.5	100	100	100	100
12	Cntnap2 -/-	3	3	12.5	62.5	100	100	100	100
13	Cntnap2 -/-	2	2	25	62.5	100	87.5	100	100
