# Supplementary material for: Comprehensive Analysis of the 16p11.2 Deletion and Null Cntnap2 Mouse Models of Autism Spectrum Disorder
Source: PLoS One. 2015 Aug 14;10(8):e0134572. doi: 10.1371/journal.pone.0134572 (PMC4537259; doi:10.1371/journal.pone.0134572)
Supplement: S34 Table — (PDF) [file pone.0134572.s049.pdf]

Startle/PPI

Number of mice	Genotype	Startle	PP74 dB	PP78 dB	PP82 dB
1	16p11.2 WT	359.56	39.12	49.83	48.55
2	16p11.2 WT	788	59.58	62.87	69.15
3	16p11.2 WT	1291.9	39.61	51.66	67.07
4	16p11.2 WT	123.6	13.34	35.48	54.37
5	16p11.2 WT	89.3	17.25	32.06	44.01
6	16p11.2 WT	929.1	16.5	21.74	39.69
7	16p11.2 WT	871.4	6.53	14.82	32.76
8	16p11.2 WT	293.1	43.16	49.57	46.86
9	16p11.2 WT	631.6	37.02	47.37	50.24
10	16p11.2 WT	648.2	20.04	38.35	56.77
11	16p11.2 WT	916.5	61.88	68.01	73.17
12	16p11.2 WT	912.2	15.73	29.32	28.02
13	16p11.2 WT	1125.8	17.25	36.38	46.31
14	16p11.2 WT	76.11	-5.51	19.85	18.76
15	16p11.2 WT	916.9	27.72	43.51	63.08
16	16p11.2 WT	93.75	12.66	28.18	-38.67
1	16p11.2 df/+	420.7	20.85	45.85	44.24
2	16p11.2 df/+	517.9	37.38	56.34	65.38
3	16p11.2 df/+	426	-1.76	6.92	44.37
4	16p11.2 df/+	257.8	45.89	44.57	65.63
5	16p11.2 df/+	724.22	26.06	36.84	44.49
6	16p11.2 df/+	154.4	36.99	42.93	51.68
7	16p11.2 df/+	84.56	4.21	6.58	30.23
8	16p11.2 df/+	822.9	40.24	67.82	72.15
9	16p11.2 df/+	550.6	18.27	24.36	38.88
10	16p11.2 df/+	521.7	19.7	29.9	37.51
11	16p11.2 df/+	764.8	9.73	10.05	31.04
12	16p11.2 df/+	861.3	2.84	14.34	29.69
13	16p11.2 df/+	457.89	30.68	45.62	57.83
14	16p11.2 df/+	504.4	23.35	40.19	33.82
15	16p11.2 df/+	290	25.79	65.37	79.91
16	16p11.2 df/+	136.3	19.4	49.69	55
1	Cntnap2 WT	620	9.79	29.37	24.85
2	Cntnap2 WT	585.4	-22	6.95	-3.01
3	Cntnap2 WT	445.7	7.54	21.07	21.97
4	Cntnap2 WT	1226.1	30.23	32.8	35.97
5	Cntnap2 WT	458.9	26.65	41.47	47.85
6	Cntnap2 WT	550.8	-6.25	14.6	45.72
7	Cntnap2 WT	581	-2.48	9.4	29.68
8	Cntnap2 WT	863.5	26.5	25.33	32.74
9	Cntnap2 WT	945.8	16.67	26	20.01
10	Cntnap2 WT	909.6	-0.87	-3.16	13.87
11	Cntnap2 WT	984.1	18.68	20.42	29.26
12	Cntnap2 WT	767.3	-8.73	-9.46	-7.87
13	Cntnap2 WT	446.8	29.48	51.66	64.21
14	Cntnap2 WT	589.6	13.25	21.79	52.7
15	Cntnap2 WT	853.4	5.18	13.59	18.35
16	Cntnap2 WT	750.9	25.89	44.57	55.41
1	Cntnap2 -/-	749.7	29.89	49.54	55.37
2	Cntnap2 -/-	539.44	-23.28	-1.88	9.35
3	Cntnap2 -/-	692.2	17.06	27.3	39.25
4	Cntnap2 -/-	521.8	28.13	50.65	46.88
5	Cntnap2 -/-	779.7	7.13	35.13	34.64
6	Cntnap2 -/-	548.9	20.42	38.35	38.4
7	Cntnap2 -/-	642.2	55.62	65.06	67.94
8	Cntnap2 -/-	565.9	23.13	31.74	36.19
9	Cntnap2 -/-	728.4	44.84	47.78	47.02
10	Cntnap2 -/-	832.4	38.42	53.94	63.74
11	Cntnap2 -/-	616.8	8.51	21.92	38.94
12	Cntnap2 -/-	959.6	15.93	16.42	48.11
13	Cntnap2 -/-	628	37.42	36.86	53.03
14	Cntnap2 -/-	774.2	31.9	37.01	57.03
15	Cntnap2 -/-	914.2	18.92	18.96	37.17
16	Cntnap2 -/-	651.9	38.84	44.56	40.05
