# Supplementary material for: Comprehensive Analysis of the 16p11.2 Deletion and Null Cntnap2 Mouse Models of Autism Spectrum Disorder
Source: PLoS One. 2015 Aug 14;10(8):e0134572. doi: 10.1371/journal.pone.0134572 (PMC4537259; doi:10.1371/journal.pone.0134572)
Supplement: S35 Table — (PDF) [file pone.0134572.s050.pdf]

Marble Burying

Number of mice	Genotype	Marbles Buried	Total Distance Traveled
1	16p11.2 WT	16	10248.6
2	16p11.2 WT	12	9348.4
3	16p11.2 WT	19	6824.3
4	16p11.2 WT	20	8930.4
5	16p11.2 WT	17	5616.1
6	16p11.2 WT	17	4863.6
7	16p11.2 WT	15	7870.6
8	16p11.2 WT	15	8624.1
9	16p11.2 WT	18	8254.6
10	16p11.2 WT	4	4388.9
11	16p11.2 WT	2	5412.0
12	16p11.2 WT	17	6799.7
13	16p11.2 WT	9	5910.8
14	16p11.2 WT	13	6529.7
15	16p11.2 WT	14	6658.9
16	16p11.2 WT	18	8940.1
1	16p11.2 df/+	18	
2	16p11.2 df/+	1	5884.0
3	16p11.2 df/+	18	12426.2
4	16p11.2 df/+	17	7607.5
5	16p11.2 df/+	18	9940.1
6	16p11.2 df/+	17	8854.3
7	16p11.2 df/+	15	7777.0
8	16p11.2 df/+	20	8046.2
9	16p11.2 df/+	16	7558.0
10	16p11.2 df/+	15	9278.9
11	16p11.2 df/+	20	13913.7
12	16p11.2 df/+	18	9680.4
13	16p11.2 df/+	0	6867.2
14	16p11.2 df/+	12	7525.4
15	16p11.2 df/+	17	9662.6
16	16p11.2 df/+	7	6521.9
1	Cntnap2 WT	13	8892.4
2	Cntnap2 WT	10	5608.9
3	Cntnap2 WT	18	9197.2
4	Cntnap2 WT	15	7030.4
5	Cntnap2 WT	18	6048.6
6	Cntnap2 WT	16	6660.7
7	Cntnap2 WT	19	8292.3
8	Cntnap2 WT	17	6850.2
9	Cntnap2 WT	17	7817.3
10	Cntnap2 WT	16	7260.8
11	Cntnap2 WT	18	9575.5
12	Cntnap2 WT	19	7207.8
13	Cntnap2 WT	14	10876.4
14	Cntnap2 WT	14	7883.0
15	Cntnap2 WT	9	8458.1
16	Cntnap2 WT	3	5862.5
1	Cntnap2 -/-	15	5672.5
2	Cntnap2 -/-	10	8654.2
3	Cntnap2 -/-	16	7387.4
4	Cntnap2 -/-	15	8125.1
5	Cntnap2 -/-	13	8128.2
6	Cntnap2 -/-	15	8172.8
7	Cntnap2 -/-	7	6006.5
8	Cntnap2 -/-	16	5419.6
9	Cntnap2 -/-	11	9201.1
10	Cntnap2 -/-	3	
11	Cntnap2 -/-	16	7412.5
12	Cntnap2 -/-	15	8954.9
13	Cntnap2 -/-	15	7646.3
14	Cntnap2 -/-	17	9062.7
15	Cntnap2 -/-	1	9749.6
16	Cntnap2 -/-	4	7901.7
